# High genetic diversity and demographic history of captive Siamese and Saltwater crocodiles suggest the first step toward the establishment of a breeding and reintroduction program in Thailand

**DOI:** 10.1371/journal.pone.0184526

**Published:** 2017-09-27

**Authors:** Sorravis Lapbenjakul, Watcharaporn Thapana, Panupon Twilprawat, Narongrit Muangmai, Thiti Kanchanaketu, Yosapong Temsiripong, Sasimanas Unajak, Surin Peyachoknagul, Kornsorn Srikulnath

**Affiliations:** 1 Laboratory of Animal Cytogenetics and Comparative Genomics (ACCG), Department of Genetics, Faculty of Science, Kasetsart University, Chatuchak, Bangkok, Thailand; 2 Animal Breeding and Genetics Consortium of Kasetsart University (ABG-KU), Kasetsart University, Bangkok, Thailand; 3 Center for Advanced Studies in Tropical Natural Resources, National Research University-Kasetsart University (CASTNAR, NRU-KU), Kasetsart University, Bangkok, Thailand; 4 Department of Fishery Biology, Faculty of Fisheries, Kasetsart University, Bangkok, Thailand; 5 Division of Genetics, Department of Science, Faculty of Liberal Arts and Science, Kasetsart University (Kamphaeng Saen Campus), Kamphaeng Saen, Nakhon Pathom, Thailand; 6 R&D Center, Sriracha Moda Co., Ltd., Sriracha, Chonburi, Thailand; 7 Department of Biochemistry, Faculty of Science, Kasetsart University, Bangkok, Thailand; 8 Department of Biology, Faculty of Science, Naresuan University, Muang Phitsanulok, Phitsanulok, Thailand; 9 Center of Excellence on Agricultural Biotechnology: (AG-BIO/PERDO-CHE), Bangkok, Thailand; University of British Columbia Okanagan, CANADA

## Abstract

The Siamese crocodile (*Crocodylus siamensis*) and Saltwater crocodile (*C*. *porosus*) are two of the most endangered animals in Thailand. Their numbers have been reduced severely by hunting and habitat fragmentation. A reintroduction plan involving captive-bred populations that are used commercially is important and necessary as a conservation strategy to aid in the recovery of wild populations. Here, the genetic diversity and population structure of 69 individual crocodiles, mostly members of captive populations, were analyzed using both mitochondrial D-loop DNA and microsatellite markers. The overall haplotype diversity was 0.924–0.971 and the mean expected heterozygosity across 22 microsatellite loci was 0.578–0.701 for the two species. This agreed with the star-like shaped topology of the haplotype network, which suggests a high level of genetic diversity. The mean ratio of the number of alleles to the allelic range (*M* ratio) for the populations of both species was considerably lower than the threshold of 0.68, which was interpreted as indicative of a historical genetic bottleneck. Microsatellite markers provided evidence of introgression for three individual crocodiles, which suggest that hybridization might have occurred between *C*. *siamensis* and *C*. *porosus*. D-loop sequence analysis detected bi-directional hybridization between male and female individuals of the parent species. Therefore, identification of genetically non-hybrid and hybrid individuals is important for long-term conservation management. Relatedness values were low within the captive populations, which supported their genetic integrity and the viability of a breeding and reintroduction management plan. This work constitutes the first step in establishing an appropriate source population from a scientifically managed perspective for an *in situ*/*ex situ* conservation program and reintroduction of crocodile individuals to the wild in Thailand.

## Introduction

The Siamese crocodile (*Crocodylus siamensis*) is a freshwater species that is commonly found in swamps and sheltered portions of rivers and streams in Thailand [1], whereas the Saltwater crocodile (*C*. *porosus*) inhabits more marine environments and typically resides in saline and brackish mangrove swamps, estuaries, deltas, lagoons, and lower stretches of rivers [2]. However, habitat destruction and illegal hunting have resulted in fragmented crocodile populations in the wild [1,3]. The severe decline of populations to fewer than 200 individuals of *C*. *siamensis* and a report from a preliminary survey of only three *C*. *porosus* observed in the wild Thai populations [4,5] led to both species being listed as Critically Endangered by the Crocodile Specialist Group of the Species Survival Commission of the International Union for Conservation of Nature (IUCN) and in Appendix I of the Convention on International Trade in Endangered Species of Wild Fauna and Flora (CITES) [2,6]. Reintroductions of captive-bred individuals and *in situ*/*ex situ* management are necessary for the recovery of wild populations of *C*. *siamensis* and *C*. *porosus*, with decisions made at the national policy level. A large number of *C*. *siamensis* individuals are maintained in 12 commercial captive facilities under the auspices of the Crocodile Co-operatives of Thailand and CITES regulations for the leather and food industries, whereas *C*. *porosus* has been managed as a captive population without commercial use. However, no new individuals have been introduced into these captive populations subsequent to their establishment and no historical information on the captive populations is available, which might lead to the loss of genetic variation. This might consequently hamper the adaptability of a captive-bred population to a new environment, the long-term sustainability of the population, and increase the probability of species extinction [7]. A captive population should be fundamentally managed to retain maximum genetic variation by avoiding inbreeding and outbreeding depression [8]. Evaluation of the genetic diversity in captive populations thus provides important information for prospective breeding and reintroduction programs, and *in situ*/*ex situ* management.

Conservation management of animal wildlife is impacted by hybridization, which can result from the possible invasion of one species into the habitat of another or involuntary mixing of species in the same farm. Such hybridization might lead to the genetic extinction of the species [9]. Hybridization has been widely reported in wild *Crocodylus* populations between Morelet’s crocodile (*C*. *moreletti*) and the American crocodile (*C*. *acutus*) and between the Cuban crocodile (*C*. *rhombifer*) and American crocodile [10–15]. Hybrids between Siamese and Cuban crocodiles or between Siamese and Saltwater crocodiles as a result of anthropogenic impacts have been observed, despite the different chromosome constitutions of these species [16–20]. Most *Crocodylus* anthropogenic hybrids are a serious problem in the context of conservation management because the hybrids are highly similar in morphology to the parental species and might lead to introgression if included in a reintroduction program. Consequently, a genetic tool must be developed to identify and exclude hybrids before reintroduction.

Assessment of genetic diversity in natural and captive populations is an important step to understand population structure, history, and hybrid status better when developing breeding programs for conservation management of threatened species [21–23]. Molecular genetic markers such as mitochondrial DNA (mtDNA) and biparentally inherited nuclear DNA microsatellites can facilitate the ability to characterize population diversity, assign possible origins of individuals, and identify hybrids and their parents [14,15,24,25]. Although the 12 captive crocodile populations in Thailand contain both *C*. *siamensis* and *C*. *porosus*, the *C*. *siamensis* captive-bred population is currently considered the most important source of individuals for a reintroduction program. The captive crocodile populations were derived from twelve major crocodile farms. These distribute crocodiles to sub-farms throughout the country to increase the Thai captive crocodile population. However, the present lack of genetic information impedes and undermines establishment of an effective long-term conservation management plan. In the study described herein, we examined genetic diversity and relatedness between captive-bred populations of *C*. *siamensis* and *C*. *porosus*, as well as wild *C*. *siamensis* individuals in the Boraphet Wetland Wildlife Reserve. We used mtDNA D-loop and microsatellite genotyping to assess the diversity of the gene pool to guide reintroductions to the wild and the formulation of *in situ*/*ex situ* management recommendations. The admixture between *C*. *siamensis* and *C*. *porosus* in captive populations is also discussed.

## Materials and methods

### Animal material and DNA extraction

Samples were collected from 47 adult *C*. *siamensis* and 17 adult *C*. *porosus* from 12 captive populations in nine provinces of Thailand (Nakhon Ratchasima: 14°58′50″N, 102°06′00″E; Lopburi: 14°48′00″N, 100°37′37″E; Bangkok: 13°45′14″N, 100°30′05″E; Ayutthaya: 14°20′52″N, 100°33′38″E; Chonburi: 13°21′40.11″N, 100°59′04.82″E; Chainat: 15°11′10″N, 100°07′24″E; Chachoengsao: 13°41′25″N, 101°04′13″E; Saraburi: 14°31′59″N, 100°55′00″E; Ratchaburi: 13°32′08″N, 99°48′48″E). Permission was granted by the farm owners and the Crocodile Co-operatives of Thailand. Five *C*. *siamensis* were captured from the Boraphet Wetland Wildlife Reserve (Nakhon Sawan: 15°42′48″N, 100°08′07″E), and all crocodiles were released immediately in the same area after sample collection. This research was conducted under the authority of the Department of Fisheries, Ministry of Agriculture and Cooperatives, Thailand. Detailed information on the sampled individuals is presented in S1 Table and S1 Fig. Individuals were classified as *C*. *siamensis* or *C*. *porosus* on the basis of morphology [26,27] and molecular mtDNA markers [28]. A piece of scale clipped from the tail of each specimen was collected as a source of DNA [29]. Whole genomic DNA was extracted in accordance with the standard salting-out protocol as described previously [30]. DNA quality and quantity were determined by electrophoresis on 1% agarose gels and spectrophotometric analysis. Animal care and all experimental procedures were approved by the Animal Experiment Committee, Kasetsart University, Thailand (approval no. ACKU04959), and conducted in accordance with the Regulations on Animal Experiments at Kasetsart University.

### D-loop sequencing

Mitochondrial D-loop DNA fragments were amplified using the primers mtCytbf2 (5′-TGCCATGTTCGCATCCATCC-3′) and mt12srRNAr2 (5′-CCAGAGGCTAGGCGTCGTGG-3′), which were designed based on five crocodilian mtDNA sequences: *C*. *siamensis* (GenBank accession number: EF581857), *C*. *porosus* (GenBank accession number: AJ810453), *C*. *niloticus* (GenBank accession number: AJ810452), *Alligator mississippiensis* (GenBank accession number: Y13113), and *Gavialis gangeticus* (GenBank accession number: AB079596). PCR amplification was performed using 15 μl of 1× ThermoPol buffer that contained 1.5 mM MgCl_2_, 0.2 mM dNTPs, 5.0 μM primers, 0.5 U of *Taq* polymerase (Apsalagen Co. Ltd., Bangkok, Thailand), and 25 ng of genomic DNA. The PCR conditions were as follows: initial denaturation at 94°C for 3 min, followed by 40 cycles of 94°C for 30 s, 55°C for 30 s, and 72°C for 1 min 30 s, and then a final extension at 72°C for 5 min. The PCR products were detected by electrophoresis on 1% agarose gels. The PCR products were cloned using the pTG19-T vector (Vivantis Technologies Sdn Bhd, Selangor Darul Ehsan, Malaysia). The nucleotide sequences of the DNA fragments were determined by the DNA sequencing service of First Base Laboratories Sdn Bhd (Seri Kembangan, Selangor, Malaysia). The BLASTn and BLASTx programs (http://blast.ncbi.nlm.nih.gov/Blast.cgi) were used to search nucleotide sequences in the National Center for Biotechnology Information (NCBI) database to confirm the identity of the DNA fragments amplified in the present study. The sequences generated in this study were deposited in the DNA Data Bank of Japan (DDBJ) (S1 Table).

### D-loop sequence analysis

Multiple sequence alignment was performed for 69 sequences generated in this study (52 for *C*. *siamensis* and 17 for *C*. *porosus* individuals), 20 sequences from other crocodile species available in the GenBank database, and one sequence from a turtle *Pelodiscus sinensis* (GenBank accession number: AY687385) as the outgroup, using the default parameters of the Molecular Evolutionary Genetics Analysis 6 (MEGA6) software (Center for Evolutionary Functional Genomics, The Biodesign Institute, Tempe, AZ, USA [31]). All unalignable and gap-containing sites were carefully removed and trimmed from the data sets. Estimates of haplotype (*h*) and nucleotide (π) diversity [32] were calculated based on mtDNA D-loop sequences as implemented in DnaSP version 5 [33]. Tests of neutral sequence evolution, namely Tajima’s *D* [34] and Fu and Li’s D* and F* tests [35], were performed using DnaSP version 5, and Fu’s *F*_s_ [36] was calculated using Arlequin version 3.5.2.2 [37]. Significance values for differences among these test results were determined using 10,000 coalescent simulations in accordance with the recommended parameters for the software. Phylogenetic analysis was performed using Bayesian inference (BI) with MrBayes version 3.2.6 [38]. The best-fit model of DNA substitution was determined for each genetic region using Kakusan4 [39]. The Markov chain Monte Carlo process was used to run four chains simultaneously for one million generations. After the log-likelihood value stabilized, a sampling procedure was performed every 100 generations to obtain 10,000 trees, from which a majority-rule consensus tree with average branch lengths was generated. All sample points prior to attaining convergence were discarded as burn-in, and the Bayesian posterior probability in the sampled tree population was calculated as a percentage. A statistical parsimony network of the consensus sequences was constructed using the Templeton, Crandall and Sing (TCS) algorithm implemented in PopART version 1.7. [40].

### Microsatellite genotyping

All 22 microsatellite primer sets that were developed originally from the Saltwater crocodile (S2 Table) [41,42] were used for genotype determination in *C*. *siamensis* and *C*. *porosus*. Most of the microsatellite loci were located in different linkage groups of the Saltwater crocodile genome. PCR amplification was performed using 15 μl of 1× ThermoPol buffer that contained 1.5 mM MgCl_2_, 0.2 mM dNTPs, 5.0 μM primers, 0.5 U of *Taq* polymerase (Apsalagen Co. Ltd., Bangkok, Thailand), and 25 ng of genomic DNA. The PCR conditions were as follows: initial denaturation at 95°C for 3 min; followed by four cycles of 95°C for 20 s, 65°C for 20 s, and 72°C for 30 s; followed by four cycles of 95°C for 20 s, 62°C for 20 s, and 72°C for 30 s; followed by eight cycles of 95°C for 20 s, 60°C for 20 s, and 72°C for 30 s; followed by 24 cycles of 95°C for 20 s, 55°C for 20 s, and 72°C for 30 s; and a final extension at 72°C for 7 min. The PCR products were separated on a 6% denaturing polyacrylamide gel (w/v) and visualized by silver staining as described previously [43]. Allele sizes were measured using the GelAnalyzer software (http://www.gelanalyzer.com/index.html). DNA fragments were extracted from the silver-stained gels and cloned into the pTG19-T vector (Vivantis Technologies). Nucleotide sequencing was carried out to confirm the identity of the DNA fragments amplified in the present study. Nucleotide sequences of all microsatellite loci for *C*. *siamensis* and *C*. *porosus* were deposited in DDBJ (S3 Table).

### Microsatellite data analysis

Allelic frequency, number of specific alleles, observed heterozygosity (*H*_o_), expected heterozygosity (*H*_e_), Hardy–Weinberg equilibrium, and linkage disequilibrium were calculated for each locus and for each captive population using Arlequin version 3.5.2.2. Shannon’s information index (*I*) was calculated for each locus for each species using GENALEX version 6.5 [44]. Polymorphic information content was estimated using the Excel Microsatellite Toolkit [45]. Reduction in heterozygosity due to non-random mating (*F*_ST_) was estimated to determine pairwise population differentiation with corrected *P* values using Arlequin version 3.5.2.2. The state of heterozygosity excess and shift in allelic frequency distributions in genetically bottlenecked populations was tested using BOTTLENECK version 1.2.02 [46]. The Wilcoxon signed rank test with a two-phased model of mutation (TPM) and stepwise mutation model (SMM) was used to obtain probabilities for excess levels of heterozygosity due to the small sample sizes of loci and small sample size. The TPM was carried out with 95% single-step mutations and 5% multistep mutations, and the variance among multiple steps was set at 12 [47]. This test detects relatively short-term bottleneck events. To test for relatively long-term bottleneck events, the *M* ratio test [48] was performed using Arlequin version 3.5.2.2. The *M* ratio is the mean number of alleles in a population divided by the allelic size range, and can indicate reductions in both recent and historical population sizes. Phylogenetic analysis of all microsatellite loci was performed using the unweighted pair group with arithmetic mean (UPGMA) clustering method using NTSYSpc version 2.1 (Exeter Software, New York, USA). Jaccard’s coefficient was used to estimate genetic similarity [49]. Bootstrap analysis was performed using FreeTree software with 500 replicates [50]. Mantel’s test was used to determine the goodness of fit for a cluster analysis. The degree of fit was interpreted as follows: 0.9 ≤ *r* (very good fit); 0.8 ≤ *r* < 0.9 (good fit); 0.7 ≤ *r* < 0.8 (poor fit); *r* < 0.7 (very poor fit). Principal component analysis (PCA) was performed to visualize the overall relationship across individuals in the populations using GENALEX version 6.5.

The model-based clustering method implemented in STRUCTURE version 2.3.3 [51] was used to determine population structure. Run length was set to 100,000 Markov chain Monte Carlo replicates after a burn-in period of 100,000 generations using correlated allelic frequencies under a straight admixture model. The number of clusters (*K*) was varied from 1 to 28, with 25 replicates for each value of *K*. The most likely number of clusters was determined by plotting the log probability of the data (ln Pr(*X*|*K*) [51] across the range of *K* values tested and selecting *K* at which the value of ln Pr(*X*|*K*) stabilized. The Δ*K* method [52] was applied with STRUCTURE HARVESTER [53]. In addition, relatedness values (*r*) [54] were estimated among individual crocodiles using GENALEX version 6.5. The individual and overall inbreeding coefficients with 95% confidence intervals were calculated using Ritland’s estimator [54] as implemented in COANCESTRY [55].

## Results

### D-loop haplotype variation

After editing, sequences for the 1,110 to 1,528 bp fragment of both *C*. *siamensis* and *C*. *porosus* D-loop into 263 bp in length, which corresponded to the fragment at positions 16,525 to 16,807 bp of the *C*. *siamensis* mitochondrial genome (GenBank accession number: EF581859), were compiled into data sets for *C*. *siamensis* and *C*. *porosus*. This sequence region was located in D-loop domain III, which is commonly used for the analysis of genetic diversity and population structure [56]. We identified 152 polymorphic sites (comprising 84 transitions and 68 transversions) in the *C*. *siamensis* data set and 41 polymorphic sites (comprising 22 transitions and 19 transversions) in the *C*. *porosus* data set. The number of haplotypes was 35 for *C*. *siamensis* and 14 for *C*. *porosus*. The overall haplotype and nucleotide diversities were 0.924 ± 0.031 and 0.031 ± 0.009, respectively, for *C*. *siamensis* and 0.971 ± 0.032 and 0.021 ± 0.006, respectively, for *C*. *porosus* (S4 Table).

**Fig 1:**
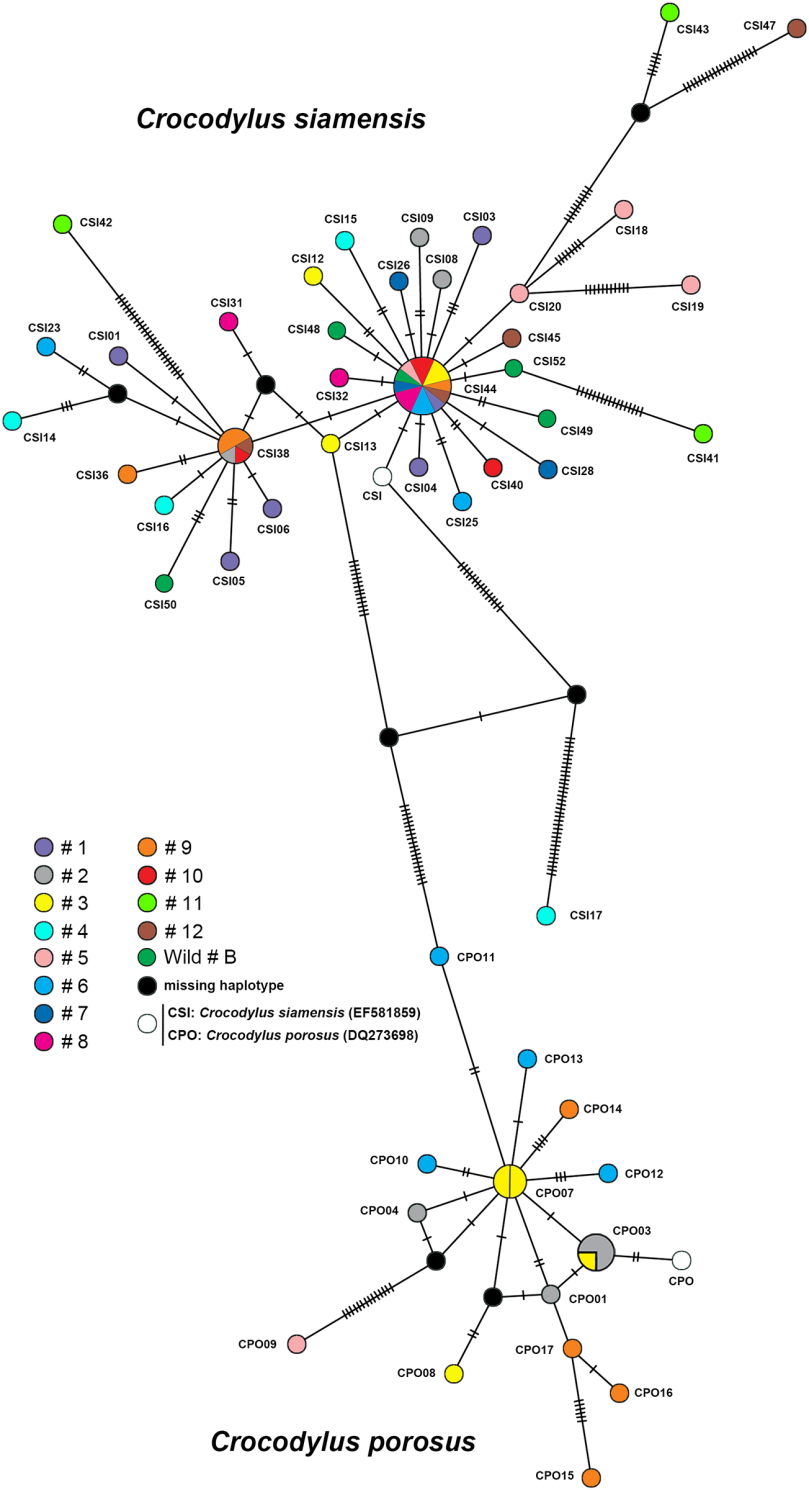
Haplotype network based on sequence data for the mitochondrial DNA D-loop region of Siamese and Saltwater crocodiles constructed using statistical parsimony with the Templeton, Crandall, and Sing (TCS) algorithm. The numbers of individuals that possessed a haplotype is indicated by the different colors inside the circles. Inferred but unsampled haplotypes are indicated by slashes. Missing haplotypes are indicated by a black circle. Detailed information for all crocodile individuals is presented in S1 Table.

**Fig 2:**
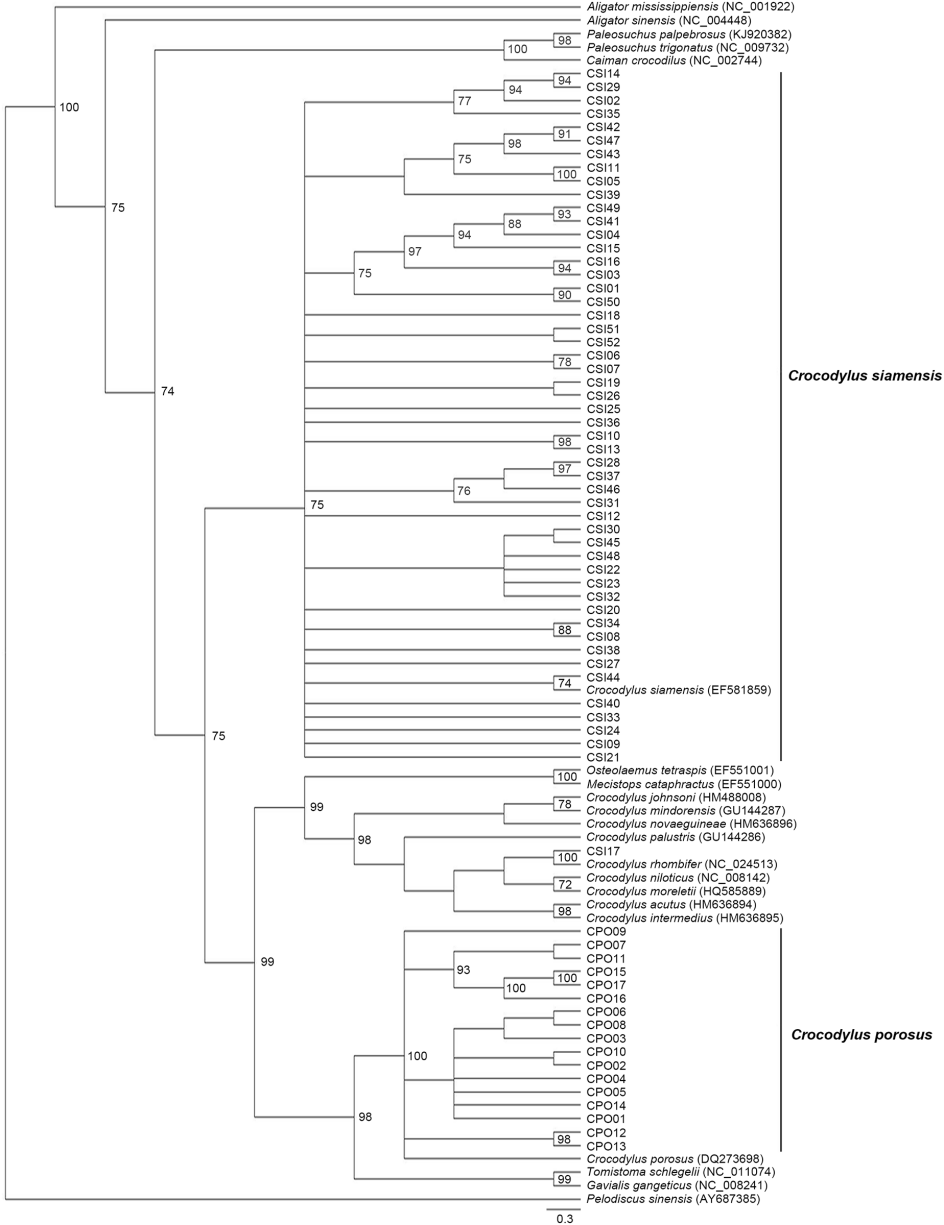
Phylogenetic relationships among mitochondrial DNA D-loop region sequences inferred using Bayesian inference analysis. Support values at each node are the Bayesian posterior probability. Detailed information for all crocodile individuals is presented in S1 Table.

The haplotype networks for both *C*. *siamensis* and *C*. *porosus* showed a star-like shaped topology (Fig 1), which indicates a high level of haplotype diversity. All *C*. *siamensis* haplotypes clustered in a group that was distinct from the *C*. *porosus* haplotypes by at least 34 mutational steps with a missing haplotype (Fig 1). The most common haplotype of *C*. *siamensis* in the population sample (CSI44) differed from shared nucleotide sequences of the haplotype of the mitochondrial genome of *C*. *siamensis* (GenBank accession number: EF581859) that had been described previously [28] by only one mutational step. An additional common haplotype of *C*. *siamensis* (CSI38) was distinguished from CSI44 by one mutational step. However, one haplotype (CSI17) was distinguished from CSI44 by at least 50 mutational steps with a missing haplotype. The most common haplotype of *C*. *porosus* differed from the haplotype of the mitochondrial genome of *C*. *porosus* (GenBank accession number: DQ273698) that had been described previously [57] by two mutational steps. Phylogenetic analysis of a combined data set for the D-loop sequences from both *C*. *siamensis* and *C*. *porosus*, together with those for 20 crocodile species obtained from the GenBank database, indicated that most sequences of *C*. *siamensis* and all sequences of *C*. *porosus* each formed a monophyletic clade. However, CSI17 was placed as a sister clade to *C*. *rhombifer* (GenBank accession number: NC_024513) (Fig 2).

Tajima’s *D* (−2.711, *P* < 0.001), Fu’s *F*_s_ (−18.013, *P* < 0.001), Fu and Li’s F* (−5.099, *P* < 0.02), and Fu and Li’s D* (−5.209, *P* < 0.02) were all negative and significant for the D-loop sequences of *C*. *siamensis*. Similarly, Tajima’s *D* (−2.240, *P* < 0.01), Fu’s *F*_s_ (−6.188, *P* < 0.02), Fu and Li’s F* (−3.322, *P* < 0.02), and Fu and Li’s D* (−3.123, *P* < 0.02) were all negative and significant for the mtDNA D-loop sequences of *C*. *porosus*.

### Genotypic variation, hybridization, and relatedness

Twenty-two microsatellite primer pairs were applied to genotype the crocodile individuals. A total of 299 alleles, which comprised 138 private alleles in *C*. *siamensis* and 82 private alleles in *C*. *porosus*, were detected among all loci, with a mean number of alleles per locus of 13.59 (S5 Table). The two crocodile species shared 79 alleles. Allelic frequencies showed significant departures from Hardy–Weinberg expectations at five loci for the *C*. *siamensis* population and eight loci for the *C*. *porosus* population with multiple lines of evidence for linkage disequilibrium (S5–S7 Tables). However, the ability to detect significant departures from Hardy–Weinberg equilibrium was low because of the small sample sizes. No consistent patterns of deviation from Hardy–Weinberg equilibrium or linkage equilibrium were detected across sites. Consequently, genetic analyses were then performed, based on all the microsatellite loci. The polymorphic information content of both crocodile species ranged from 0.038 to 0.937, and Shannon’s information index ranged from 0.108 to 3.148 (S5 Table). The *H*_o_ values of *C*. *siamensis* ranged from 0.000 (CpP3001) to 1.000 (CpP4501) (mean 0.486 ± 0.306) and *H*_e_ values ranged from 0.038 (CpP3008) to 0.949 (CpP501) (mean 0.578 ± 0.323). The *H*_o_ values of *C*. *porosus* ranged from 0.059 (CpP3001) to 0.941 (CpP1409) (mean 0.591 ± 0.253) and *H*_e_ values ranged from 0.059 (CpP3001) to 0.939 (CpP501) (mean 0.701 ± 0.221) (S5 and S8 Tables). After 110 permutations, estimates of *F*_ST_ showed significant differences between captive and wild populations of crocodiles, which indicated substantial genetic subdivision (S9 and S10 Tables). In the test for population bottlenecks, SMM and TPM were 0.997 and 0.982, respectively, in *C*. *siamensis* (normal L-shaped mode shift), and 0.999 and 0.996, respectively, in *C*. *porosus* (normal L-shaped mode shift) as determined by Wilcoxon sign-rank tests (S11 Table). Although some populations showed evidence of reduction in population size, overall results did not detect a significant reduction in *C*. *siamensis* or *C*. *porosus*. However, the *M* ratio across all populations averaged 0.264 ± 0.191 for *C*. *siamensis* and 0.252 ± 0.155 for *C*. *porosus* (S5 Table). These *M* ratio values were lower than the 0.68 threshold identified by Garza and Williamson [48], which indicated a historical population reduction.

**Fig 3:**
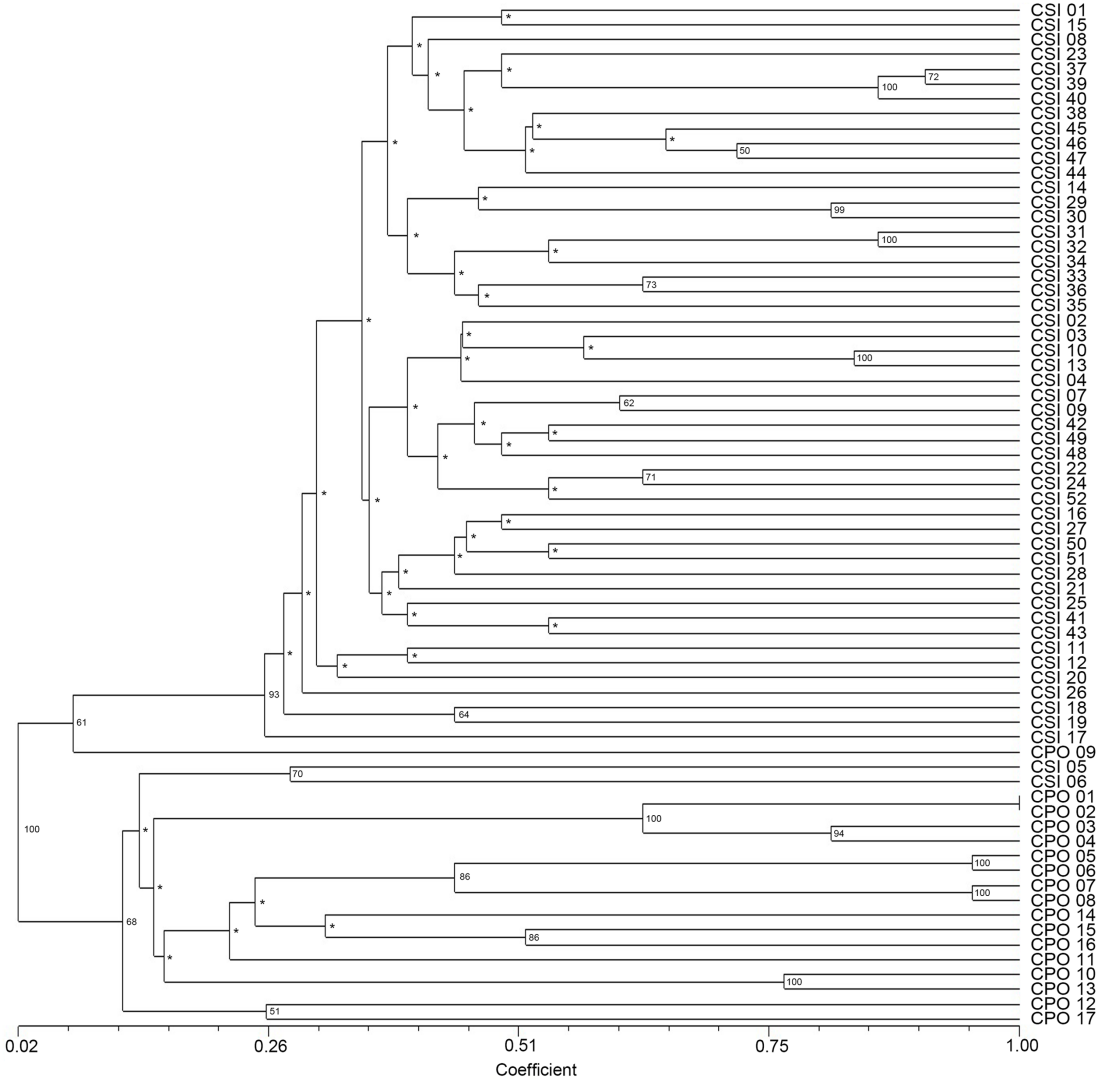
Microsatellite-based phylogenetic relationships for 52 Siamese crocodiles and 17 Saltwater crocodiles generated by the unweighted pair group with arithmetic mean (UPGMA) clustering method. Support values at each node are bootstrap values. “*” indicates a bootstrap value < 50%. The genetic similarity matrices are shown by Jaccard's coefficient. Detailed information for all crocodile individuals is presented in S1 Table.

**Fig 4:**
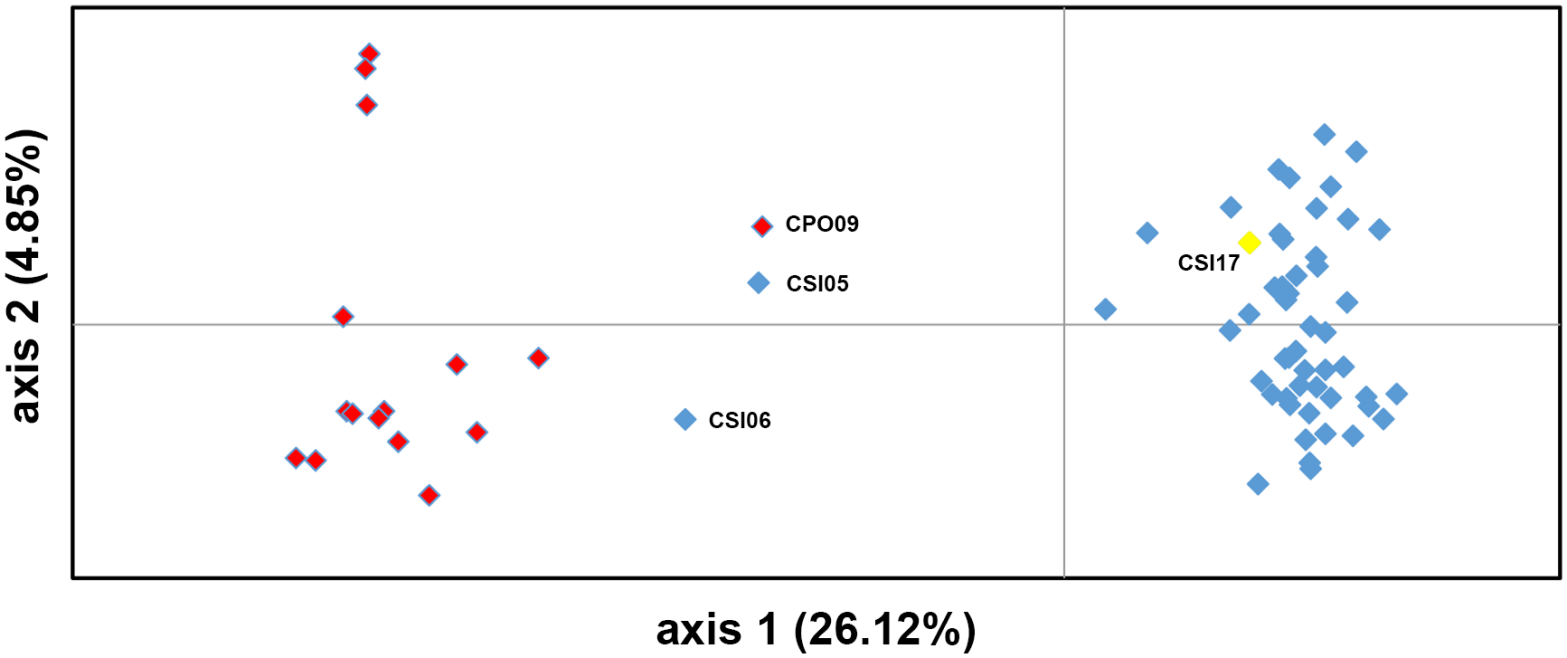
Principal component analysis of 69 crocodiles (52 Siamese crocodiles and 17 Saltwater crocodiles) using microsatellite data across 22 loci. Blue diamonds indicate Siamese crocodile samples. Red diamonds indicate Saltwater crocodile samples. The yellow diamond indicates CSI17, which might be a Cuban crocodile or a hybrid between Siamese and Cuban crocodiles.

Phylogenetic analysis of the 69 samples divided the crocodile individuals into two major groups (*C*. *siamensis* and *C*. *porosus*) (Fig 3). The cophenetic coefficient of Mantel’s test indicated a very good fit for the cluster analysis (*r* = 0.959). The *C*. *porosus* group comprised 16 *C*. *porosus* individuals and two *C*. *siamensis* individuals (CSI05 and CSI06), whereas the *C*. *siamensis* group contained 50 *C*. *siamensis* individuals and one *C*. *porosus* individual (CPO09) (Fig 3). PCA revealed that the first, second, and third principal components accounted for 26.12%, 4.85%, and 4.47% of the total variation, respectively, and provided support for the distinction between the two crocodile groups and admixture of the aforementioned three individuals (CSI05, CSI06, and CPO09) (Fig 4).

**Fig 5:**
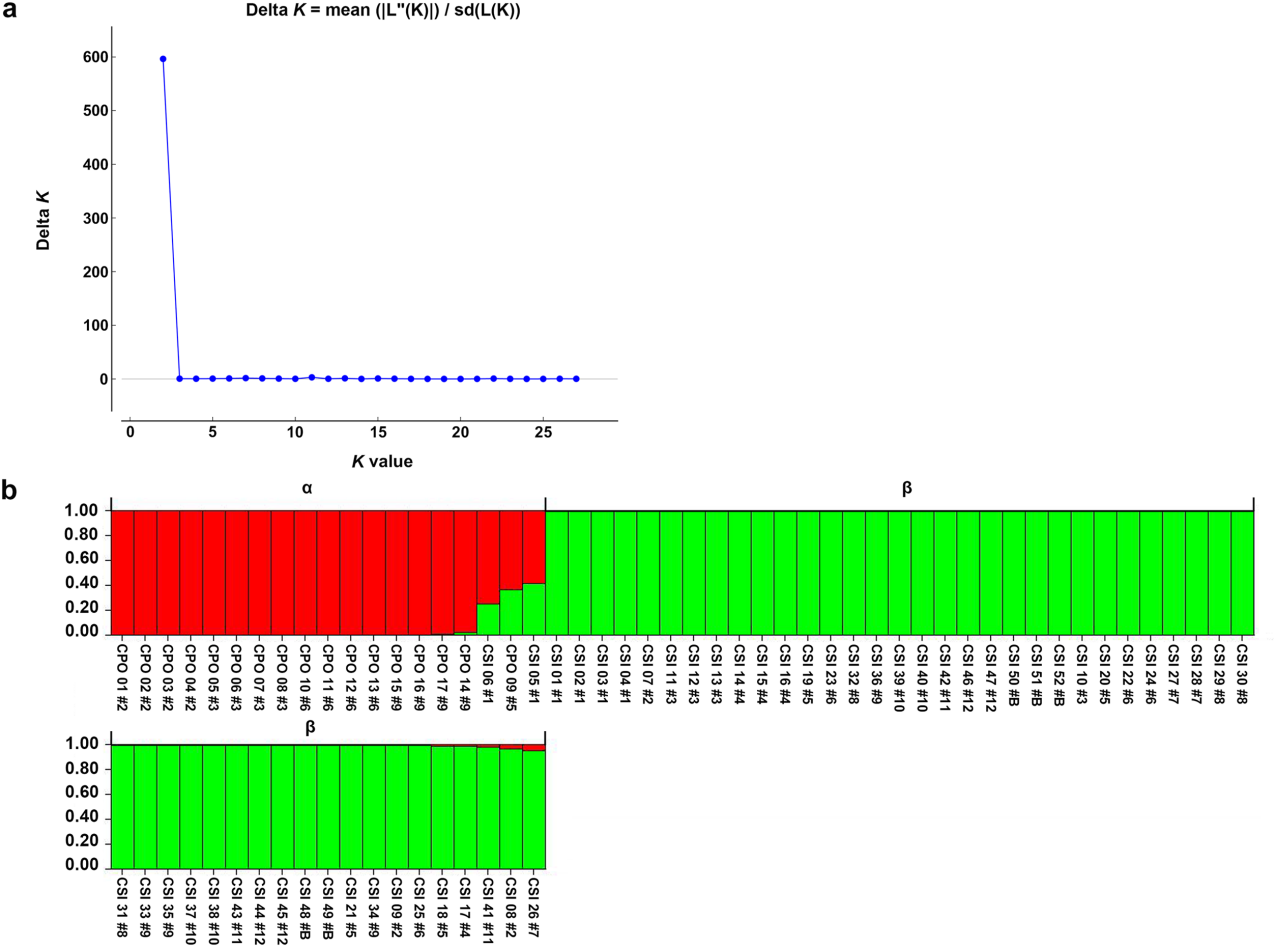
Population structure of Siamese and Saltwater crocodiles from 69 crocodile individuals. (a) Evanno's Δ*K* graph. (b) STRUCTURE bar plots depicting the model-based clustering results for inferred *K* = 2. Inferred genetic clusters are displayed as different colors. Each vertical bar on the *x*-axis represents an individual, and the *y*-axis presents the proportion of membership (posterior probability) in each genetic cluster. Recovered crocodile species, including clusters α and β, are superimposed on the plot, with black vertical lines indicating the boundaries. Detailed information for all crocodile individuals is presented in S1 Table.

**Fig 6:**
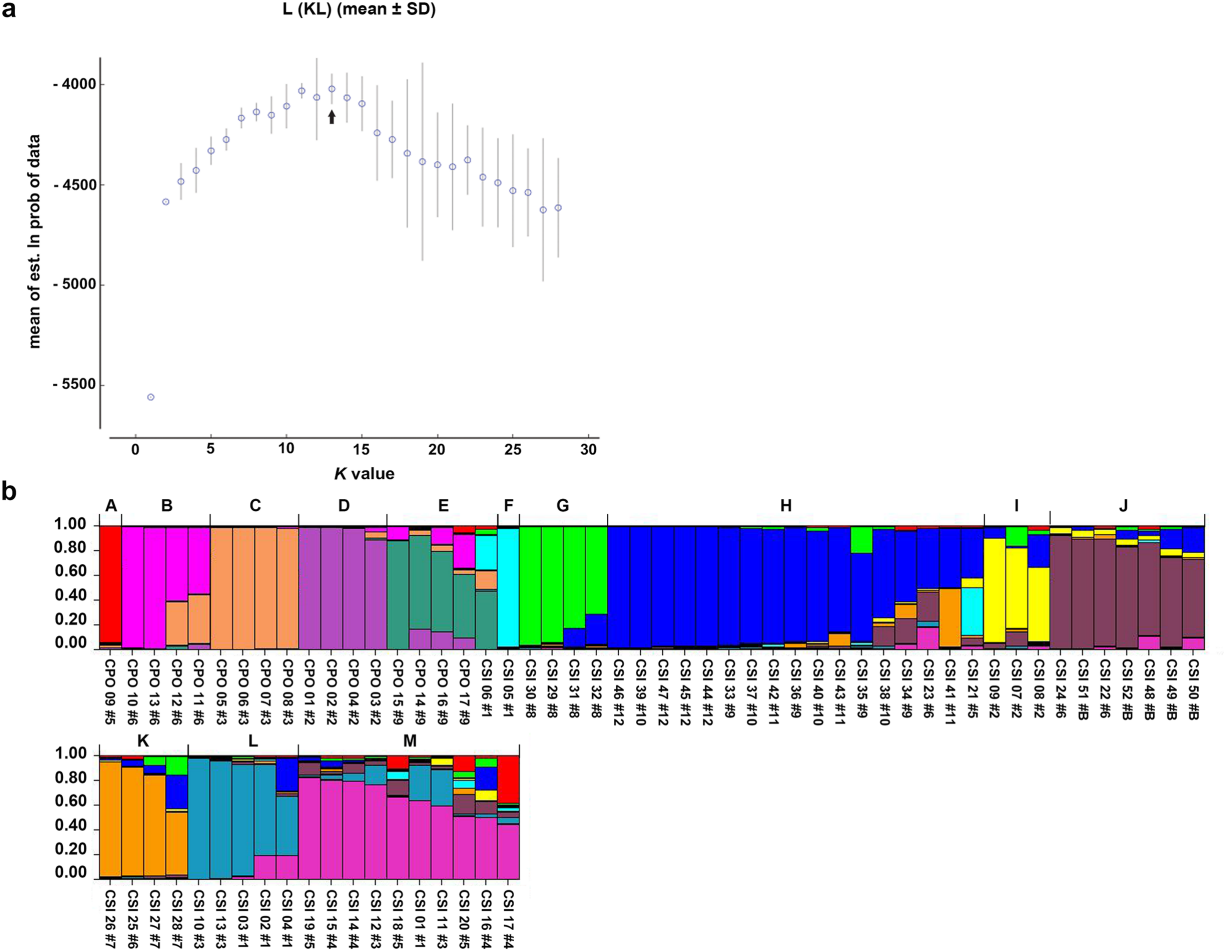
Population structure of Siamese and Saltwater crocodiles from 69 crocodile individuals. (a) Mean Ln P(*K*) graph. (b) STRUCTURE bar plots depict the model-based clustering results for inferred *K* = 13. Inferred genetic clusters are displayed as different colors. Each vertical bar on the *x*-axis represents an individual, and the *y*-axis presents the proportion of membership (posterior probability) in each genetic cluster. Recovered crocodile species, including clusters A–M, are superimposed on the plot, with black vertical lines indicating the boundaries. Detailed information for all crocodile individuals is presented in S1 Table.

**Fig 7:**
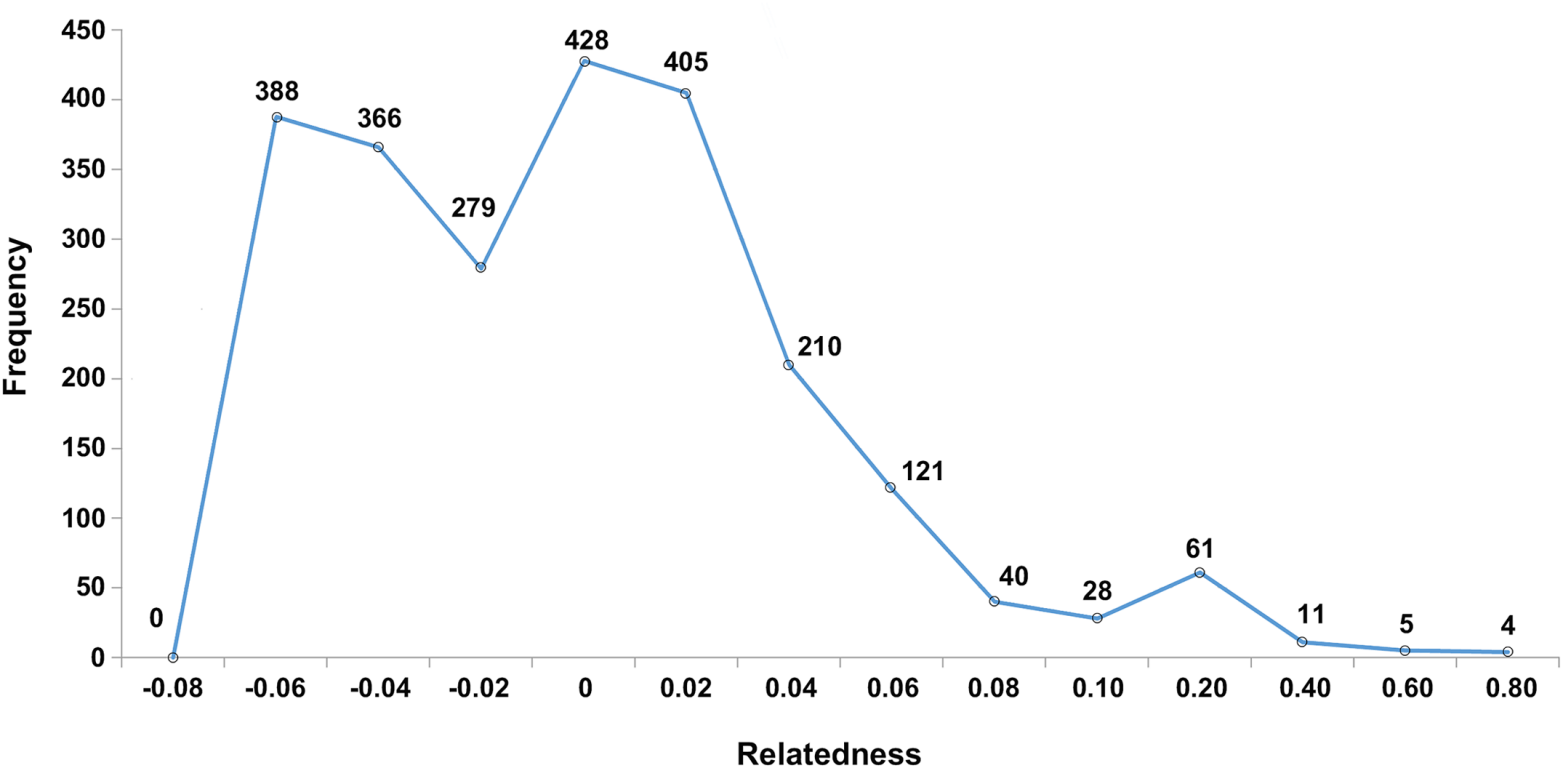
Observed distribution of pairwise relatedness for 52 Siamese crocodiles and 17 Saltwater crocodiles plotted against expected distributions.

Structure analysis revealed the highest posterior probability with one peak (*K* = 2) on the basis of Evanno's Δ*K* (Fig 5a), with all crocodiles grouped into two clusters, α and β, which corresponded to *C*. *porosus* and *C*. *siamensis*, respectively (Fig 5b). Two *C*. *siamensis* individuals (CSI05 and CSI06) and one *C*. *porosus* individual (CPO09) showed high estimated levels of admixture, whereas low levels of admixture were estimated in CPO14, CSI08, CSI17, CSI18, CSI26, and CSI41. By contrast, STRUCTURE analysis based on the mean ln P(*K*) revealed one peak (*K* = 13) (Fig 6a), which provided evidence for 13 clusters (Fig 6b). Clusters A–E represented *C*. *porosus*, whereas clusters F–M comprised *C*. *siamensis*. A pairwise relatedness test was performed to determine the level of relatedness between individuals in the study population (Fig 7). The relatedness values (*r*) of 2,346 pairs of crocodiles among the 69 sampled crocodiles were examined, and the mean pairwise value was −0.009. A total of 1,449 pairs showed *r* < 0; there were 892 pairs with 0.5 < *r* < 0; and five pairs with 1< *r* < 0.5 (S12 Table). The mean inbreeding coefficient was 0.186, with individual inbreeding coefficients ranging from −0.016 to 0.824 (S13 Table).

## Discussion

The meat, clothing, and medicinal products that are derived from *C*. *siamensis* constitute 1% of the agricultural income in Thailand [58]. Crocodile numbers are increasing in all Thai crocodile farms, which is contrary to the decreasing number of individuals observed in the wild. Consequently, CITES regulations permit only limited exportation of captive-bred crocodile products until the reintroduction program and *in situ*/*ex situ* management are sustainable. The first step toward establishment of a captive breeding and reintroduction program is the evaluation of genetic diversity based on analysis of mtDNA D-loop sequences and microsatellite markers as an indicator of the genetic status of captive crocodile populations in the main commercial farms.

### Diversity and population structure

Although nucleotide diversity was low, analysis of mtDNA D-loop sequences indicated that haplotype diversity was relatively high in the sampled individuals of *C*. *porosus* and *C*. *siamensis*. This finding was probably attributable to the high haplotype heterogeneity in the study population. Concomitantly, microsatellite analysis showed that the mean *H*_o_ and *H*_e_ were 0.486 and 0.578, respectively, for *C*. *siamensis* and 0.591 and 0.701, respectively, for *C*. *porosus*. These values are similar to those of the Siamese crocodile population in Vietnam (*H*_o_ = 0.416 and *H*_e_ = 0.420) [19] and the Saltwater crocodile population in Palau (*H*_o_ = 0.570 and *H*_e_ = 0.575) [59], which suggests that the populations of both species show a state of high genetic diversity. Differentiation among populations was also detected in both captive and wild populations of *C*. *siamensis*. *F*_ST_ values were 0.073–0.240 (*P* < 0.05) from 50 pairwise captive/wild population comparisons in *C*. *siamensis* and 0.200–0.356 (*P* < 0.05) from five pairwise captive population comparisons in *C*. *porosus*, which might indicate high heterozygosity. This suggests that captive-bred Thai populations of both *C*. *siamensis* and *C*. *porosus* can be recommended for use in breeding programs and reintroduction plans.

### Population history of captive *C*. *siamensis* and *C*. *porosus*

Network analyses revealed the existence of four different haplotype groups, of which two were in the *C*. *siamensis* haplotype group. The most common haplotype (CSI44) was closely related to the haplotype of three of the five individuals (CSI48, CSI49, and CSI52) that were sampled from the Boraphet Wetland Wildlife Reserve. Many unique haplotypes were also detected, which suggests that populations of *C*. *siamensis* had been isolated from each other. The star-like shaped topology of the haplotype network of *C*. *siamensis* suggests a high level of genetic heterogeneity. A similar pattern was observed for the *C*. *porosus* haplotype network, although wild individuals were not available for study. This star-like network might also imply the presence of a population bottleneck followed by a population expansion. Significant negative values for neutrality statistics also indicate population expansion [60]. This finding suggests that the captive crocodile populations originated from wild-caught crocodiles and were subsequently bred within each captive breeding program to produce a large number of individuals. Unfortunately, the original source of most captive-bred populations is unknown. Although a demographic bottleneck was not supported by the bottleneck test, the *M* ratio showed a signal for historical population reduction for both *C*. *siamensis* and *C*. *porosus*. Collectively, these results suggest that these captive crocodile populations underwent a recent population expansion. Crocodile hunting to collect individuals has occurred over the last 50 years [6,61], with subsequent successful breeding and an increase in the size of captive populations. Low haplotype heterogeneity is usually observed with rapid population expansion [62]. However, in the present study, captive populations of both *C*. *siamensis* and *C*. *porosus* showed high haplotype heterogeneity. This suggests that individuals from various historically distinct lineages in the wild might have contributed to the established populations, leading to the presence of individuals with high levels of admixture. Range expansion and high haplotype diversity were aided by intentional human introduction in each captive population.

A missing haplotype was detected between most *C*. *siamensis* and *C*. *porosus* haplotype groups, which determined that the two crocodile species were genetically distinct [28,63]. However, an additional missing haplotype was detected between CSI17 and all the remaining *C*. *siamensis* haplotypes. This finding corresponded with the results of the microsatellite phylogenetic analysis in which CSI17 was not placed in the cluster that contained most *C*. *siamensis* individuals. Cuban crocodiles have also been identified in captive breeding programs in Cambodia and Vietnam [19,64], and the admixture of crocodile individuals in Cambodia, Vietnam, and Thailand might result from partial introgression in captive breeding programs over a long period [17,18,65]. The present results suggest that CSI17 is at least the result of hybridization with a *C*. *rhombifer*, if not a pure Cuban crocodile.

### Hybridization between *C*. *siamensis* and *C*. *porosus*

The present microsatellite phylogenetic analyses and PCA indicated that three individuals (CPO09, CSI05, and CSI06) might be the result of interspecific hybridization between *C*. *siamensis* and *C*. *porosus*. Cluster analysis using the STRUCTURE software can help to determine the degree of hybridization by aggregating individuals into a single cluster relative to additional highly differentiated populations/species [15,51]. Using a posterior probability of 0.95 as a criterion for assignment to a pure species as estimated with STRUCTURE [12], the three individuals (CPO09, CSI05, and CSI06) showed evidence of admixture. Both CPO09 and CSI05 were placed in strongly differentiated clusters (A and F) that were distinct from most *C*. *siamensis* and *C*. *porosus* individuals, respectively, and possessed a high proportion of private alleles. By contrast, CSI06 clustered with *C*. *porosus*. Observations on captive crocodiles suggest that bi-directional hybridization occurs between *C*. *siamensis* and *C*. *porosus* [18]. This conclusion is in agreement with the results of the D-loop sequence analysis. CPO09 shared a mitochondrial haplotype with *C*. *porosus*, which suggests that hybridization probably occurred between a female *C*. *porosus* and a male *C*. *siamensis*. By contrast, CSI05 and CSI06 shared haplotypes with *C*. *siamensis*, which suggest that they are descended from a female *C*. *siamensis* and a male *C*. *porosus*.

Natural hybridization often occurs in the genus *Crocodylus* [13,19]. However, hybrids between *C*. *siamensis* and *C*. *porosus* are always observed in the presence of anthropogenic impacts. This might reflect behavioral and geographic differences between the two species [11,12,15,19]. Moreover, the occurrence of fertile hybrids might be rare as a consequence of the different chromosome constitutions of the two species [18,19]. Hybridization is a serious conservation concern when anthropogenic factors cause misclassification of individuals on the basis of morphology, such as between Siamese and Saltwater crocodiles [18,19]. This might lead to backcrossing, resulting in localized hybrids, widespread introgression, or complete admixture [9]. The removal of hybrids is beneficial to sustainable use programs because local commercial captive operations can utilize the genetic material of hybrid crocodiles for industry. Alternatively, we strongly suggest that efforts to avoid hybridization are taken into account in conservation management and reintroduction programs because hybrids often show superior survival and adaptive mechanisms under competition with non-hybrid individuals [66]. This scenario also elicits the likelihood of genetic pollution of the species by more abundant hybrids.

The present D-loop and microsatellite data indicate that the captive populations of *C*. *siamensis* and *C*. *porosus* are genetically divergent, with partial introgression and hybrids between the two species. An alternative approach is required to identify hybrid or non-hybrid individuals in addition to karyotyping [20]. A larger population sample and more detailed analysis are required to estimate the degree of hybridization, such as analysis of F_1_, F_2_, or backcross generations. The costs of molecular analytic methods have decreased greatly and the use of such procedures should be a prerequisite for breeding management and the establishment of a reintroduction program, rather than a karyological analysis approach. It is strongly recommended that researchers conduct routine molecular analyses to identify individuals of hybrid origin.

### Implications for conservation and management

Inbreeding has negative impacts on both reproduction and survival [67,68]. The mean inbreeding coefficient was 0.137 for *C*. *siamensis* and 0.334 for *C*. *porosus*, and a high level of mean relatedness was indicated (0.008 and 0.109 for both species, respectively), implying that the samples were not closely related [54]. These results suggest that the populations of *C*. *porosus* and *C*. *siamensis* were founded initially by individuals of unknown ancestry. Mate pairings within representative captive crocodile populations might be determined to aid breeding and reintroduction programs. In general, breeding that is based on the minimization of relatedness generates maximum genetic diversity [67]. Prior to the current study, captive breeding and reintroduction programs for *C*. *siamensis* and *C*. *porosus* in Thailand have proceeded in the absence of genetic diversity data, and have relied solely on morphological, ethological, demographic, and logistic information for the implementation of short-term management strategies [69,70]. The present results provide an important genetic baseline for *in situ*/*ex situ* management decisions. The populations of both species exhibited high heterozygosity, which could be an indication of the sound establishment of the captive species populations. Specifically, 10 individuals of *C*. *siamensis* (19.231%) comprising six males and four females, and seven individuals of *C*. *porosus* (43.750%) consisting of four males and three females, were highlighted as genetically important for conservation and management, because they showed values of relatedness that were lower than that of the overall captive population. This provides flexibility in the implementation of breeding and reintroduction programs. However, one possible concern for reintroduction is the likelihood of disrupting locally adapted genetic materials through outbreeding among different source populations [71]. This remains an inherent problem because captive crocodile populations typically contain individuals that originate from widely scattered populations. Consideration of the minimization of relatedness and relationship of haplotype lineage with the source population are necessary to propose management action. Finding suitable habitats for the introduction of the two species is an additional problem in Thailand [69]. Although the present study is preliminary, the results are important for ongoing conservation and genetic management programs both locally and throughout the distribution ranges of the species. We believe the substantial captive crocodile populations that were sampled in this study to be broadly representative of captive-bred populations in Thailand as a whole. This work constitutes the first step in establishing an appropriate source population from a scientifically managed perspective for *in situ*/*ex situ* conservation and reintroduction programs in Thailand. In addition, the present results provide reference data for further characterization of cryptic diversity, which directly impacts on the conservation prioritization of *C*. *siamensis* and *C*. *porosus* outside Thailand, such as in Southeast Asia.

The results of the present study indicate the status of genetic diversity in the extant Thai captive crocodile population. Accurate information on captive populations for breeding programs, reintroduction, or *in situ*/*ex situ* management will aid the management of subsequent generations and maintain sustainable genetic diversity for long-term survival of the population. Therefore, it is highly recommended that the genetic status of *C*. *siamensis* and *C*. *porosus* populations are closely monitored using molecular genetic methods to improve *in situ*/*ex situ* management. We are also convinced that the future conservation management of *C*. *siamensis* will require the genetic identification of non-hybrid wild populations. Additional studies involving genome-wide scans will be required for an improved understanding of overall gene functions in different populations.

## Supporting information

S1 Fig Map showing the collection sites for the *Crocodylus siamensis* and *C*. *porosus* specimens. Numbers indicate sample locality. Detailed information for all crocodile individuals is presented in S1 Table.
(JPG)

S1 Table Summary of crocodile specimens. (DOCX)

S2 Table List of microsatellite primers and sequences used in the study. (DOCX)

S3 Table Sequence accession numbers of 22 microsatellite loci from one representative individual of the Siamese crocodile (*Crocodylus siamensis*) and one representative individual of the Saltwater crocodile (*C*. *porosus*). (DOCX)

S4 Table Mitochondrial DNA D-loop diversity based on a 263-bp fragment for the Siamese crocodile (*Crocodylus siamensis*) and Saltwater crocodile (*C*. *porosus*). *** *P* < 0.001, ** *P* < 0.02, * *P* < 0.01, and ns = not significant.
(DOCX)

S5 Table Genetic diversity of 52 individuals of the Siamese crocodile (*Crocodylus siamensis*) and 17 individuals of the Saltwater crocodile (*C*. *porosus*) based on 22 microsatellite loci. (DOCX)

S6 Table Pairwise differentiation of linkage disequilibrium among Siamese crocodile (*Crocodylus siamensis*) individuals based on 22 microsatellite loci. The number indicates *P* values, with 110 permutations.
(DOCX)

S7 Table Pairwise differentiation of linkage disequilibrium among Saltwater crocodile (*Crocodylus porosus*) individuals based on 22 microsatellite loci. The number indicates *P* values, with 110 permutations.
(DOCX)

S8 Table Comparison of observed and expected heterozygosity of the Siamese crocodile (*Crocodylus siamensis*) and Saltwater crocodile (*C*. *porosus*) based on 22 microsatellite loci in each captive/wild population. Detailed information for all crocodile individuals is presented in S1 Table.
(DOCX)

S9 Table Pairwise genetic differentiation (*F*_ST_) between Siamese crocodile (*Crocodylus siamensis*) captive/wild populations based on 22 microsatellite loci. The number indicates *P* values, with 110 permutations. Detailed information for all crocodile individuals is presented in S1 Table.
(DOCX)

S10 Table Pairwise genetic differentiation (*F*_ST_) between Saltwater crocodile (*Crocodylus porosus*) captive populations based on 22 microsatellite loci. The number indicates *P* values, with 110 permutations. Detailed information for all crocodile individuals is presented in S1 Table.
(DOCX)

S11 Table Test for genetic bottlenecks in the Siamese crocodile (*Crocodylus siamensis*) and Saltwater crocodile (*C*. *porosus*) using BOTTLENECK version 1.2.02 and calculation of the *M* ratio using Arlequin 3.5.2.2 for all populations. Detailed information for all crocodile individuals is presented in S1 Table.
(DOCX)

S12 Table Pairwise genetic relatedness (*r*) for all 69 crocodile individuals. Detailed information for all crocodile individuals is presented in S1 Table.
(DOCX)

S13 Table Pairwise inbreeding coefficients for all 69 crocodile individuals. Detailed information for all crocodile individuals is presented in S1 Table.
(DOCX)
