# Supplementary figures and images for: High genetic diversity and demographic history of captive Siamese and Saltwater crocodiles suggest the first step toward the establishment of a breeding and reintroduction program in Thailand
Source: PLoS One. 2017 Sep 27;12(9):e0184526. doi: 10.1371/journal.pone.0184526 (PMC5617146; doi:10.1371/journal.pone.0184526)

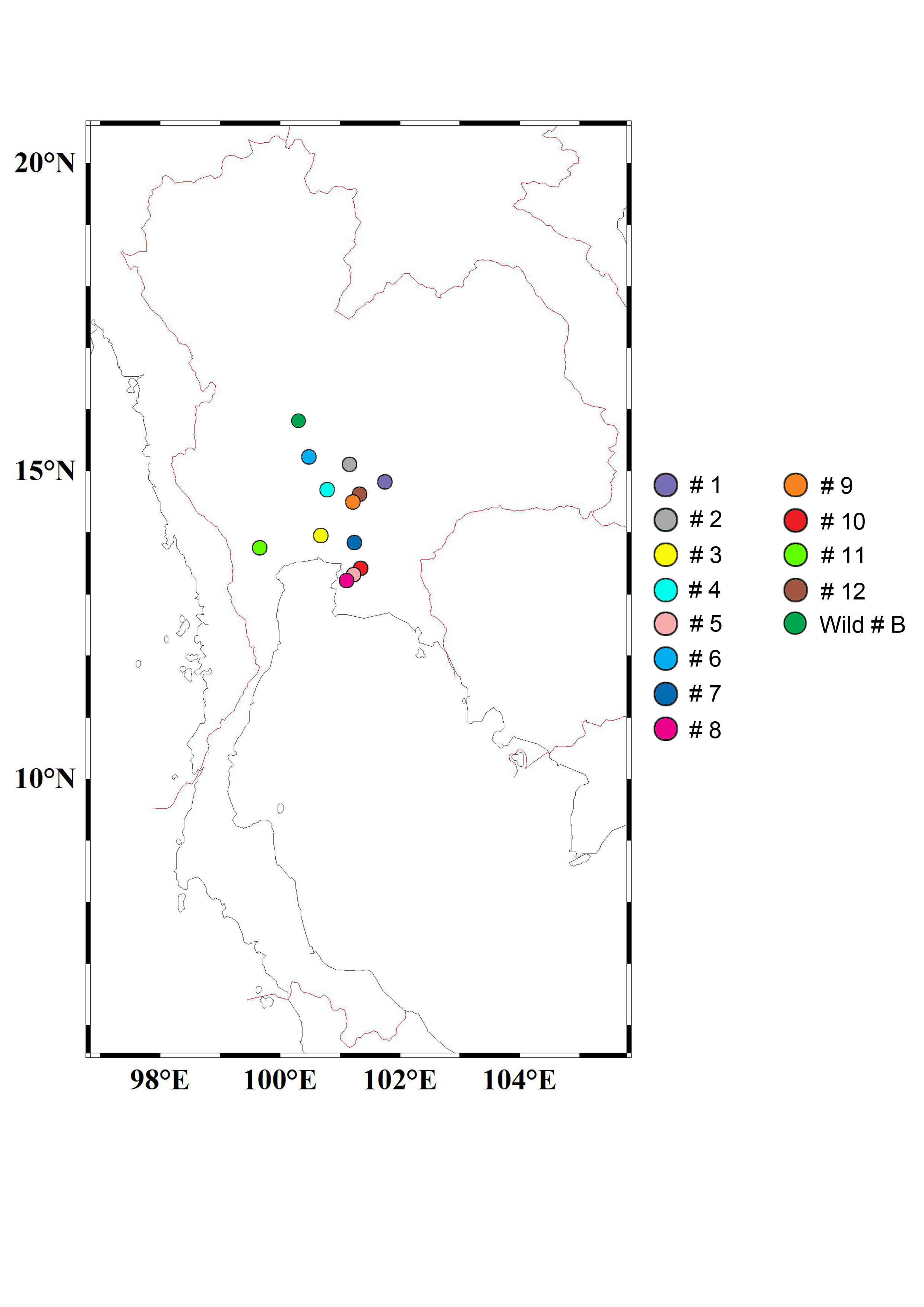

Supplement: S1 Fig — Numbers indicate sample locality. Detailed information for all crocodile individuals is presented in S1 Table. (JPG) [file pone.0184526.s001.jpg]
