# Supplementary material for: High genetic diversity and demographic history of captive Siamese and Saltwater crocodiles suggest the first step toward the establishment of a breeding and reintroduction program in Thailand
Source: PLoS One. 2017 Sep 27;12(9):e0184526. doi: 10.1371/journal.pone.0184526 (PMC5617146; doi:10.1371/journal.pone.0184526)
Supplement: S1 Table — (DOCX) [file pone.0184526.s002.docx]

**S1 Table.** **Summary of crocodile specimens.**

| Number | Captive/wild population | Locality | Geographic coordinates | Number of Siamese crocodile sample | Sex | *Crocodylus siamensis* Code | Mitochondrial DNA D-loop GenBank accession number | Number of Saltwater crocodile sample | Sex | *Crocodylus porosus* Code | Mitochondrial DNA D-loop GenBank accession number |
| --- | --- | --- | --- | --- | --- | --- | --- | --- | --- | --- | --- |
| # 1 | Captivity # 1 | Nakhon  Ratchasima | 14°58′50″N,  102°06′00″E | 6 | 3M, 3F | CSI01 – 06 | LC191661 – LC191666 | - | - | - | - |
| # 2 | Captivity # 2 | Lopburi | 14°48′00″N,  100°37′37″E | 3 | 2M, 1F | CSI07 – 09 | LC191666 – LC191669 | 4 | 4F | CPO01 – 04 | LC191712 – LC191715 |
| # 3 | Captivity # 3 | Bangkok | 13°45′14″N,  100°30′05″E | 4 | 2M, 2F | CSI10 – 13 | LC191670 – LC191673 | 4 | 2M, 2F | CPO05 – 08 | LC191716 – LC191719 |
| # 4 | Captivity # 4 | Ayutthaya | 14°20′52″N,  100°33′38″E | 4 | 2M, 2F | CSI14 – 17 | LC191674 – LC191677 | - | - | - | - |
| # 5 | Captivity # 5 | Chonburi | 13°21′40.11″N, 100°59′04.82″E | 4 | 2M, 2F | CSI18 – 21 | LC191215, LC191678 – LC191680 | 1 | 1M | CPO09 | LC191216 |
| # 6 | Captivity # 6 | Chainat | 15°11′10″N,  100°07′24″E | 4 | 2M, 2F | CSI22 – 25 | LC191681 – LC191684 | 4 | 2M, 2F | CPO10 – 13 | LC191720 – LC191723 |
| # 7 | Captivity # 7 | Chachoeng  sao | 13°41′25″N,  101°04′13″E | 3 | 1M, 2F | CSI26 – 28 | LC191685 – LC191687 | - | - | - | - |
| # 8 | Captivity # 8 | Chonburi | 13°21′40.11″N, 100°59′04.82″E | 4 | 2M, 2F | CSI29 – 32 | LC191688 – LC191691 | - | - | - | - |
| # 9 | Captivity # 9 | Saraburi | 14°31′59″N,  100°55′00″E | 4 | 2M, 2F | CSI33 – 36 | LC191692 – LC191695 | 4 | 2M, 2F | CPO14 – 17 | LC191724 – LC191727 |
| # 10 | Captivity # 10 | Chonburi | 13°21′40.11″N, 100°59′04.82″E | 4 | 2M, 2F | CSI37 – 40 | LC191696 – LC191699 | - | - | - | - |
| # 11 | Captivity # 11 | Ratchaburi | 13°32′08″N,  99°48′48″E | 3 | 2M, 1F | CSI41 – 43 | LC191700 – LC191702 | - | - | - | - |
| # 12 | Captivity # 12 | Saraburi | 14°31′59″N,  100°55′00″E | 4 | 2M, 2F | CSI44 – 47 | LC191703 – LC191706 | - | - | - | - |
| # 13 | Wild # B (Boraphet Wetland Wild Reserve) | Nakhon Sawan | 15°42′48″N,  100°08′07″E | 5 | 3M, 2F | CSI48 – 52 | LC191707 – LC191711 | - | - | - | - |

M; Male, F: Female
