# Supplementary material for: High genetic diversity and demographic history of captive Siamese and Saltwater crocodiles suggest the first step toward the establishment of a breeding and reintroduction program in Thailand
Source: PLoS One. 2017 Sep 27;12(9):e0184526. doi: 10.1371/journal.pone.0184526 (PMC5617146; doi:10.1371/journal.pone.0184526)
Supplement: S2 Table — (DOCX) [file pone.0184526.s003.docx]

**S2 Table. List of microsatellite primers and sequences used in the study.**

| Primers | | Primer sequences 5′ to 3′ | |
| --- | --- | --- | --- |
| CpP 208 | | CAGTCGGGCGTCATCACACATGGCTTTTGTTCTGAG | |
|  | | GTTTCCTGCAAAATGTTCTCCTA | |
| CpP 501 | | CAGTCGGGCGTCATCACCTGATAGACTGCCTACAA | |
|  | | GTTTGTTAGTTCCCACTGAAGAAG | |
| CpP 1002 | | CAGTCGGGCGTCATCATTTGGGCTCCACAATTC | |
|  | | GTTTCATCCCTTGGAGCTCTT | |
| CpP 209 | | GTTTACTTAGGGTTTGCTAGTCAC | |
|  | | CAGTCGGGCGTCATCATACTGCTAATGCGAGACA | |
| CpP 214 | | CAGTCGGGCGTCATCAGTCTGTCTTTGCAGTCTTG | |
|  | | GTTTTCCTGGAATAAACTGACTA | |
| CpP 1308 | | CAGTCGGGCGTCATCAACCTGAAAATGGATACTG | |
|  | | GTTTACGCTTGTTAACTTCACT | |
| CpP 203 | | CAGTCGGGCGTCATCAGTCCATTGCCAGTTGTAA | |
|  | | GTTTCTGAGGGTGAACTTTAGAA | |
| CpP 2206 | | GTTTAGGCCAGTTCTTATCTACAT | |
|  | | CAGTCGGGCGTCATCAAAGTTCTCCCCACTAAAG | |
| CpP 4004 | | CAGTCGGGCGTCATCACTGAATTGGGTGGAATAG | |
|  | | GTTTATCCACATTTTTCCATGAC | |
| CpP 3303 | | CAGTCGGGCGTCATCATGTCTATTTTTGCCTTGTTT | |
|  | | GTTTGCCTAATTGCCTGAATAA | |
| CpF 509 | | CAGTCGGGCGTCATCAACACAAAGGAGCATACAC | |
|  | | GTTTAGCCAATTCCCATATCT | |
| CpP 3008 | | CAGTCGGGCGTCATCAACAACTGGCACATCTCA | |
|  | | GTTTCCCGTAGCCTCCTACTG | |
| CpP 2904 | | CAGTCGGGCGTCATCAGGTTTCCTCCACACAA | |
|  | | GTTTCTATGCCACTAACATTAACAG | |
| CpP 4501 | | CAGTCGGGCGTCATCATATACACACATGCACCTCTT | |
|  | | GTTTGCACAGCCCTAATAGA | |
| CpP 1201 | | CAGTCGGGCGTCATCAGCAGATGTGGCAAATAGTTC | |
|  | | GTTTAAACTCGCCTCACTACAGA | |
| CpP 3004 | | CAGTCGGGCGTCATCAGGAGTGAATCTATGCCAGC | |
|  | | GTTTAAAATGTTTTCATATGGTCG | |
| CpP 3313 | | CAGTCGGGCGTCATCACTTCTGTTACTTAGGGACTG | |
|  | | GTTTAAAAACCCAGGCAAATA | |
| CpP 3001 | | CAGTCGGGCGTCATCAGTAGCATGGCATAAGTGT | |
|  | | GTTTAGAATGCCATAAATCACAT | |
| CpP 1409 | | GTTTATGCCCTACTGGTTATCTATC | |
|  | | CAGTCGGGCGTCATCAGGGAAGGGGATTTAATAAT | |
| CpP 3508 | | CAGTCGGGCGTCATCAAGGGGATGTAAACACT | |
|  | | GTTTATAACTATCATTGCCATTGT | |
| CpP 2504 | | CAGTCGGGCGTCATCACTCATATTTCCCAACTATCAC | |
|  | | GTTTCATTCCCACAATACACATAA | |
| CpP 3219 | CAGTCGGGCGTCATCAGCAAGGGCATATAATAGTTC  GTTTAAGTTGCCTTAAAATTGTAAA | |  |
|  | |  | |
|  | |  | |
|  | |  | |
|  | |  | |
|  | |  | |
|  | |  | |
|  | |  | |
|  | |  | |
|  | |  | |
|  | |  | |
|  | |  | |
|  | |  | |
|  | |  | |
|  | |  | |
|  | |  | |
|  | |  | |
|  | |  | |
|  | |  | |
|  | |  | |
|  | |  | |
|  | |  | |
|  | |  | |
|  | |  | |
|  | |  | |
|  | |  | |
|  | |  | |
|  | |  | |
|  | |  | |
|  | |  | |
|  | |  | |
|  | |  | |
|  | |  | |
|  | |  | |
|  | |  | |
|  | |  | |
|  | |  | |
|  | |  | |
|  | |  | |
|  | |  | |
|  | |  | |
|  | |  | |
|  |  | |  |
|  |  | |  |
|  |  | |  |
