# Supplementary material for: High genetic diversity and demographic history of captive Siamese and Saltwater crocodiles suggest the first step toward the establishment of a breeding and reintroduction program in Thailand
Source: PLoS One. 2017 Sep 27;12(9):e0184526. doi: 10.1371/journal.pone.0184526 (PMC5617146; doi:10.1371/journal.pone.0184526)
Supplement: S3 Table — (DOCX) [file pone.0184526.s004.docx]

**S3 Table.** **Sequence accession numbers of 22 microsatellite loci from one representative individual of the Siamese crocodile (*Crocodylus siamensis*) and one representative individual of the Saltwater crocodile (*C. porosus*).**

| Locus | Accession number | |
| --- | --- | --- |
|  | Siamese crocodile | Saltwater crocodile |
| CpP208 | LC260540 | LC260541 |
| CpP501 | LC260536 | LC260537 |
| CpP1002 | LC260538 | LC260539 |
| CpP209 | LC260542 | LC260543 |
| CpP214 | LC260546 | LC260547 |
| CpP1308 | LC260544 | LC260545 |
| CpP203 | LC269373 | LC260548 |
| CpP2206 | LC260549 | LC269096 |
| CpP4004 | LC260550 | LC269099 |
| CpP3303 | LC269376 | LC260551 |
| CpF509 | LC269375 | LC269374 |
| CpP4501 | LC260555 | LC260556 |
| CpP1201 | LC269378 | LC269377 |
| CpP3004 | LC260557 | LC260558 |
| CpP3313 | LC260559 | LC260560 |
| CpP3508 | LC269379 | LC260564 |
| CpP1409 | LC260562 | LC260563 |
| CpP3008 | LC260552 | LC260186 |
| CpP2904 | LC260553 | LC260554 |
| CpP2504 | LC260565 | LC260566 |
| CpP3219 | LC269098 | LC269097 |
| CpP3001 | LC269100 | LC260561 |
