# Supplementary material for: High genetic diversity and demographic history of captive Siamese and Saltwater crocodiles suggest the first step toward the establishment of a breeding and reintroduction program in Thailand
Source: PLoS One. 2017 Sep 27;12(9):e0184526. doi: 10.1371/journal.pone.0184526 (PMC5617146; doi:10.1371/journal.pone.0184526)
Supplement: S4 Table — *** P < 0.001, ** P < 0.02, * P < 0.01, and ns = not significant. (DOCX) [file pone.0184526.s005.docx]

**S4 Table.** **Mitochondrial DNA D-loop diversity based on a 263-bp fragment for the Siamese crocodile (*Crocodylus siamensis*) and Saltwater crocodile (*C. porosus*).** *** *P* < 0.001, ** *P* < 0.02, * *P* < 0.01, and ns = not significant.

| Species | Captivity/Wild | Sample size | Number of haplotype | Total number of mutation | Haplotype diversity (*h*) | % Nucleotide diversity (π) | Tajima's *D* | Fu and Li *D** | Fu and Li *F** | Fu's *Fs* |
| --- | --- | --- | --- | --- | --- | --- | --- | --- | --- | --- |
| *Crocodylus siamensis* | # 1 | 6 | 6 | 9 | 1.000±0.096 | 0.012±0.002 | -1.020^ns^ | -1.117^ns^ | -1.181^ns^ | -3.079^**^ |
|  | # 2 | 3 | 3 | 4 | 1.000±0.272 | 0.010±0.003 | - | - | - | -0.341^ns^ |
|  | # 3 | 4 | 3 | 3 | 0.833±0.222 | 0.006±0.002 | -0.754^ns^ | -0.754^ns^ | -0.675^ns^ | -0.288^ns^ |
|  | # 4 | 4 | 4 | 57 | 1.000±0.177 | 0.108±0.048 | -0.868^ns^ | -0.757^ns^ | -0.827^ns^ | 1.495^ns^ |
|  | # 5 | 5 | 4 | 20 | 1.000±0.177 | 0.037±0.012 | -1.011^***^ | -0.854^ns^ | -0.915^ns^ | 0.314^ns^ |
|  | # 6 | 4 | 3 | 6 | 0.833±0.222 | 0.011±0.004 | -0.809^ns^ | -0.809^ns^ | -0.777^ns^ | 0.731^ns^ |
|  | # 7 | 3 | 3 | 2 | 1.000±0.272 | 0.005±0.002 | - | - | - | -1.216^ns^ |
|  | # 8 | 4 | 3 | 4 | 0.833±0.222 | 0.007±0.003 | -0.780^ns^ | -0.780^ns^ | -0.721^ns^ | 0.134^ns^ |
|  | # 9 | 4 | 3 | 3 | 0.833±0.222 | 0.006±0.002 | -0.754^ns^ | -0.754^ns^ | -0.675^ns^ | -0.288^ns^ |
|  | # 10 | 4 | 3 | 3 | 0.833±0.222 | 0.006±0.002 | -0.754^ns^ | -0.754^ns^ | -0.675^ns^ | -0.288^ns^ |
|  | # 11 | 3 | 3 | 46 | 1.000±0.272 | 0.115±0.032 | - | - | - | 2.292^ns^ |
|  | # 12 | 4 | 4 | 31 | 1.000±0.177 | 0.060±0.029 | -0.760^ns^ | -0.760^ns^ | -0.799^ns^ | 0.845^ns^ |
|  | Wild # B | 5 | 5 | 8 | 1.000±0.126 | 0.012±0.003 | -1.174^ns^ | -1.174^ns^ | -1.230^ns^ | -2.116^ns^ |
| *Crocodylus porosus* | # 2 | 4 | 3 | 3 | 0.833±0.222 | 0.006±0.002 | -0.754^ns^ | -0.754^ns^ | -0.675^ns^ | -0.288^ns^ |
|  | # 3 | 4 | 4 | 4 | 0.833±0.222 | 0.008±0.003 | -0.780^ns^ | -0.780^ns^ | -0.720^ns^ | 0.134^ns^ |
|  | # 5 | 1 | - | - | - | - | - | - | - | - |
|  | # 6 | 4 | 4 | 8 | 1.000±0.177 | 0.015±0.003 | -0.824^ns^ | -0.824^ns^ | -0.811^ns^ | -0.825^ns^ |
|  | # 9 | 4 | 4 | 13 | 1.000±0.177 | 0.025±0.007 | -0.843^ns^ | -0.843^ns^ | -0.856^ns^ | -0.187^ns^ |
