# Supplementary material for: High genetic diversity and demographic history of captive Siamese and Saltwater crocodiles suggest the first step toward the establishment of a breeding and reintroduction program in Thailand
Source: PLoS One. 2017 Sep 27;12(9):e0184526. doi: 10.1371/journal.pone.0184526 (PMC5617146; doi:10.1371/journal.pone.0184526)
Supplement: S5 Table — (DOCX) [file pone.0184526.s006.docx]

**S5 Table.** **Genetic diversity of 52 individuals of the Siamese crocodile (*Crocodylus siamensis*) and 17 individuals of the Saltwater crocodile (*C. porosus*) based on 22 microsatellite loci.**

| Species | Locus | N | Na | I | *H_o_* | *H_e_* | PIC | *M* ratio | *P* |
| --- | --- | --- | --- | --- | --- | --- | --- | --- | --- |
| *Crocodylus siamensis* | CpP208 | 52 | 6 | 0.311 | 0.058 | 0.112 | 0.110 | 0.051 | 0.000 |
|  | CpP501 | 52 | 35 | 3.148 | 0.865 | 0.949 | 0.937 | 0.188 | 0.000 |
|  | CpP1002 | 52 | 3 | 0.149 | 0.019 | 0.057 | 0.056 | 0.023 | 0.009 |
|  | CpP209 | 52 | 13 | 2.018 | 0.538 | 0.822 | 0.795 | 0.289 | 0.000 |
|  | CpP214 | 52 | 13 | 2.236 | 0.481 | 0.884 | 0.863 | 0.245 | 0.000 |
|  | CpP1308 | 52 | 6 | 0.795 | 0.462 | 0.460 | 0.383 | 0.107 | 0.807 |
|  | CpP203 | 52 | 23 | 2.687 | 0.923 | 0.915 | 0.900 | 0.147 | 0.000 |
|  | CpP2206 | 52 | 6 | 1.034 | 0.750 | 0.552 | 0.482 | 0.261 | 0.000 |
|  | CpP4004 | 52 | 10 | 1.976 | 0.538 | 0.840 | 0.813 | 0.313 | 0.000 |
|  | CpP3303 | 52 | 5 | 0.771 | 0.404 | 0.433 | 0.370 | 0.192 | 0.880 |
|  | CpF509 | 52 | 13 | 2.323 | 0.692 | 0.889 | 0.869 | 0.325 | 0.000 |
|  | CpP4501 | 52 | 4 | 1.211 | 1.000 | 0.674 | 0.606 | 0.800 | 0.000 |
|  | CpP1201 | 52 | 26 | 2.739 | 0.558 | 0.912 | 0.896 | 0.133 | 0.000 |
|  | CpP3004 | 52 | 4 | 0.718 | 0.404 | 0.457 | 0.366 | 0.333 | 0.013 |
|  | CpP3313 | 52 | 7 | 1.703 | 0.481 | 0.805 | 0.768 | 0.233 | 0.000 |
|  | CpP3508 | 52 | 2 | 0.193 | 0.019 | 0.092 | 0.087 | 0.333 | 0.001 |
|  | CpP1409 | 52 | 16 | 2.382 | 0.788 | 0.900 | 0.881 | 0.410 | 0.000 |
|  | CpP3008 | 52 | 3 | 0.108 | 0.038 | 0.038 | 0.038 | 0.019 | 1.000 |
|  | CpP2904 | 52 | 4 | 0.786 | 0.481 | 0.522 | 0.402 | 0.364 | 0.626 |
|  | CpP2504 | 52 | 8 | 1.362 | 0.423 | 0.638 | 0.596 | 0.082 | 0.000 |
|  | CpP3219 | 52 | 8 | 1.440 | 0.769 | 0.689 | 0.640 | 0.296 | 0.165 |
|  | CpP3001 | 52 | 2 | 0.163 | 0.000 | 0.075 | 0.071 | 0.667 | 0.000 |
| Mean |  |  | 9.864 | 1.375 | 0.486 | 0.578 | 0.542 | 0.264 | - |
| S.D. |  |  | 8.554 | 0.951 | 0.306 | 0.323 | 0.320 | 0.191 | - |
| *Crocodylus porosus* | CpP208 | 17 | 7 | 1.422 | 0.647 | 0.701 | 0.634 | 0.056 | 0.003 |
|  | CpP501 | 17 | 14 | 2.525 | 0.882 | 0.939 | 0.905 | 0.177 | 0.000 |
|  | CpP1002 | 17 | 9 | 1.828 | 0.471 | 0.800 | 0.756 | 0.079 | 0.000 |
|  | CpP209 | 17 | 7 | 1.707 | 0.235 | 0.815 | 0.760 | 0.219 | 0.000 |
|  | CpP214 | 17 | 8 | 1.351 | 0.353 | 0.643 | 0.584 | 0.381 | 0.000 |
|  | CpP1308 | 17 | 7 | 0.996 | 0.471 | 0.455 | 0.421 | 0.082 | 0.410 |
|  | CpP203 | 17 | 13 | 2.372 | 0.882 | 0.920 | 0.883 | 0.178 | 0.017 |
|  | CpP2206 | 17 | 6 | 1.205 | 0.706 | 0.595 | 0.546 | 0.182 | 0.294 |
|  | CpP4004 | 17 | 6 | 1.360 | 0.412 | 0.692 | 0.629 | 0.261 | 0.003 |
|  | CpP3303 | 17 | 8 | 1.709 | 0.765 | 0.795 | 0.743 | 0.205 | 0.084 |
|  | CpF509 | 17 | 5 | 1.430 | 0.353 | 0.763 | 0.696 | 0.227 | 0.000 |
|  | CpP4501 | 17 | 5 | 1.196 | 0.647 | 0.652 | 0.572 | 0.500 | 0.110 |
|  | CpP1201 | 17 | 12 | 2.364 | 0.824 | 0.923 | 0.887 | 0.115 | 0.000 |
|  | CpP3004 | 17 | 6 | 1.692 | 0.765 | 0.825 | 0.772 | 0.429 | 0.313 |
|  | CpP3313 | 17 | 7 | 1.707 | 0.882 | 0.815 | 0.760 | 0.219 | 0.008 |
|  | CpP3508 | 17 | 2 | 0.362 | 0.118 | 0.214 | 0.186 | 0.333 | 0.178 |
|  | CpP1409 | 17 | 9 | 1.814 | 0.941 | 0.807 | 0.760 | 0.310 | 0.005 |
|  | CpP3008 | 17 | 9 | 1.930 | 0.588 | 0.845 | 0.800 | 0.056 | 0.000 |
|  | CpP2904 | 17 | 3 | 0.846 | 0.706 | 0.542 | 0.436 | 0.600 | 0.054 |
|  | CpP2504 | 17 | 8 | 1.886 | 0.588 | 0.852 | 0.805 | 0.111 | 0.000 |
|  | CpP3219 | 17 | 8 | 1.648 | 0.706 | 0.763 | 0.712 | 0.320 | 0.064 |
|  | CpP3001 | 17 | 2 | 0.133 | 0.059 | 0.059 | 0.055 | 0.500 | 1.000 |
| Mean |  |  | 7.318 | 1.522 | 0.591 | 0.701 | 0.650 | 0.252 | - |
| S.D. |  |  | 3.092 | 0.592 | 0.253 | 0.221 | 0.217 | 0.155 | - |
| All | CpP208 | 69 | 11 | 1.082 | 0.203 | 0.469 | 0.444 | 0.088 | 0.000 |
|  | CpP501 | 69 | 44 | 3.435 | 0.870 | 0.964 | 0.955 | 0.237 | 0.000 |
|  | CpP1002 | 69 | 11 | 1.050 | 0.130 | 0.432 | 0.414 | 0.084 | 0.000 |
|  | CpP209 | 69 | 17 | 2.412 | 0.464 | 0.884 | 0.867 | 0.378 | 0.000 |
|  | CpP214 | 69 | 15 | 2.210 | 0.449 | 0.864 | 0.843 | 0.263 | 0.000 |
|  | CpP1308 | 69 | 11 | 1.341 | 0.464 | 0.656 | 0.603 | 0.125 | 0.000 |
|  | CpP203 | 69 | 27 | 2.840 | 0.913 | 0.927 | 0.915 | 0.172 | 0.000 |
|  | CpP2206 | 69 | 8 | 1.505 | 0.739 | 0.709 | 0.665 | 0.242 | 0.000 |
|  | CpP4004 | 69 | 11 | 1.971 | 0.507 | 0.835 | 0.808 | 0.344 | 0.000 |
|  | CpP3303 | 69 | 11 | 1.409 | 0.493 | 0.647 | 0.604 | 0.205 | 0.000 |
|  | CpF509 | 69 | 14 | 2.349 | 0.609 | 0.886 | 0.869 | 0.333 | 0.000 |
|  | CpP4501 | 69 | 5 | 1.243 | 0.913 | 0.672 | 0.606 | 0.500 | 0.000 |
|  | CpP1201 | 69 | 32 | 3.021 | 0.623 | 0.936 | 0.925 | 0.164 | 0.000 |
|  | CpP3004 | 69 | 8 | 1.470 | 0.493 | 0.680 | 0.638 | 0.381 | 0.000 |
|  | CpP3313 | 69 | 10 | 1.854 | 0.580 | 0.814 | 0.783 | 0.313 | 0.000 |
|  | CpP3508 | 69 | 2 | 0.566 | 0.043 | 0.381 | 0.307 | 0.333 | 0.000 |
|  | CpP1409 | 69 | 20 | 2.670 | 0.826 | 0.925 | 0.913 | 0.435 | 0.000 |
|  | CpP3008 | 69 | 10 | 0.971 | 0.174 | 0.402 | 0.386 | 0.062 | 0.000 |
|  | CpP2904 | 69 | 5 | 1.035 | 0.536 | 0.599 | 0.513 | 0.385 | 0.000 |
|  | CpP2504 | 69 | 14 | 1.996 | 0.464 | 0.784 | 0.762 | 0.121 | 0.000 |
|  | CpP3219 | 69 | 10 | 1.823 | 0.754 | 0.788 | 0.757 | 0.370 | 0.000 |
|  | CpP3001 | 69 | 3 | 0.622 | 0.014 | 0.406 | 0.327 | 0.500 | 0.000 |
| Mean |  |  | 13.591 | 1.767 | 0.512 | 0.712 | 0.678 | 0.278 | - |
| S.D. |  |  | 9.825 | 0.788 | 0.269 | 0.193 | 0.208 | 0.133 | - |

Column headings are: sample size (N); number of alleles (Na); shannon's information index (I); observed heterozygosity (*H_o_*); expected heterozygosity (*H_e_*); polymorphic information content values (PIC); *M* ratio test (*M* ratio); *P* value against Hardy–Weinberg equilibrium (*P* < 0.05).
