# Supplementary material for: High genetic diversity and demographic history of captive Siamese and Saltwater crocodiles suggest the first step toward the establishment of a breeding and reintroduction program in Thailand
Source: PLoS One. 2017 Sep 27;12(9):e0184526. doi: 10.1371/journal.pone.0184526 (PMC5617146; doi:10.1371/journal.pone.0184526)
Supplement: S6 Table — The number indicates P values, with 110 permutations. (DOCX) [file pone.0184526.s007.docx]

| Locus | CpP208 | CpP501 | CpP1002 | CpP209 | CpP214 | CpP1308 | CpP203 | CpP2206 | CpP4004 | CpP3303 | CpF509 | CpP4501 | CpP1201 | CpP3004 | CpP3313 | CpP3508 | CpP1409 | CpP3008 | CpP2904 | CpP2504 | CpP3219 | CpP3001 |
| --- | --- | --- | --- | --- | --- | --- | --- | --- | --- | --- | --- | --- | --- | --- | --- | --- | --- | --- | --- | --- | --- | --- |
| CpP208 | 0.000 |  |  |  |  |  |  |  |  |  |  |  |  |  |  |  |  |  |  |  |  |  |
| CpP501 | 0.134 | 0.000 |  |  |  |  |  |  |  |  |  |  |  |  |  |  |  |  |  |  |  |  |
| CpP1002 | 0.000 | 0.002 | 0.000 |  |  |  |  |  |  |  |  |  |  |  |  |  |  |  |  |  |  |  |
| CpP209 | 0.000 | 0.000 | 0.000 | 0.000 |  |  |  |  |  |  |  |  |  |  |  |  |  |  |  |  |  |  |
| CpP214 | 0.055 | 0.003 | 0.001 | 0.000 | 0.000 |  |  |  |  |  |  |  |  |  |  |  |  |  |  |  |  |  |
| CpP1308 | 0.000 | 0.354 | 0.002 | 0.000 | 0.617 | 0.000 |  |  |  |  |  |  |  |  |  |  |  |  |  |  |  |  |
| CpP203 | 0.029 | 0.207 | 0.258 | 0.005 | 0.010 | 0.013 | 0.000 |  |  |  |  |  |  |  |  |  |  |  |  |  |  |  |
| CpP2206 | 0.001 | 0.563 | 0.002 | 0.032 | 0.413 | 0.031 | 0.281 | 0.000 |  |  |  |  |  |  |  |  |  |  |  |  |  |  |
| CpP4004 | 0.295 | 0.261 | 0.255 | 0.000 | 0.045 | 0.365 | 0.069 | 0.918 | 0.000 |  |  |  |  |  |  |  |  |  |  |  |  |  |
| CpP3303 | 0.021 | 0.674 | 0.002 | 0.229 | 0.008 | 0.002 | 0.031 | 0.064 | 0.257 | 0.000 |  |  |  |  |  |  |  |  |  |  |  |  |
| CpF509 | 0.963 | 0.000 | 0.700 | 0.000 | 0.000 | 0.606 | 0.075 | 0.132 | 0.000 | 0.698 | 0.000 |  |  |  |  |  |  |  |  |  |  |  |
| CpP4501 | 0.022 | 0.089 | 0.013 | 0.137 | 0.001 | 0.186 | 0.002 | 0.000 | 0.982 | 0.043 | 0.147 | 0.000 |  |  |  |  |  |  |  |  |  |  |
| CpP1201 | 0.091 | 0.198 | 0.393 | 0.014 | 0.001 | 0.059 | 0.032 | 0.137 | 0.001 | 0.016 | 0.058 | 0.821 | 0.000 |  |  |  |  |  |  |  |  |  |
| CpP3004 | 0.001 | 0.305 | 0.017 | 0.020 | 0.816 | 0.051 | 0.001 | 0.005 | 0.606 | 0.047 | 0.413 | 0.389 | 0.116 | 0.000 |  |  |  |  |  |  |  |  |
| CpP3313 | 0.517 | 0.006 | 0.500 | 0.000 | 0.003 | 0.830 | 0.668 | 0.121 | 0.016 | 0.200 | 0.014 | 0.247 | 0.005 | 0.001 | 0.000 |  |  |  |  |  |  |  |
| CpP3508 | 0.000 | 0.002 | 0.000 | 0.006 | 0.005 | 0.010 | 0.033 | 0.000 | 0.182 | 0.014 | 0.822 | 0.011 | 0.147 | 0.001 | 0.015 | 0.000 |  |  |  |  |  |  |
| CpP1409 | 0.218 | 0.011 | 0.121 | 0.002 | 0.000 | 0.737 | 0.253 | 0.041 | 0.006 | 0.029 | 0.000 | 0.304 | 0.065 | 0.002 | 0.003 | 0.140 | 0.000 |  |  |  |  |  |
| CpP3008 | 1.000 | 0.517 | 1.000 | 0.131 | 0.516 | 0.141 | 0.189 | 0.737 | 0.048 | 0.588 | 0.625 | 0.925 | 0.875 | 0.246 | 0.612 | 1.000 | 0.652 | 0.000 |  |  |  |  |
| CpP2904 | 0.221 | 0.049 | 0.239 | 0.093 | 0.160 | 0.096 | 0.438 | 0.960 | 0.442 | 0.376 | 0.577 | 0.626 | 0.491 | 0.496 | 0.226 | 0.459 | 0.442 | 0.049 | 0.000 |  |  |  |
| CpP2504 | 0.011 | 0.226 | 0.003 | 0.001 | 0.059 | 0.005 | 0.477 | 0.007 | 0.222 | 0.565 | 0.340 | 0.137 | 0.357 | 0.093 | 0.001 | 0.000 | 0.441 | 0.019 | 0.020 | 0.000 |  |  |
| CpP3219 | 0.051 | 0.067 | 0.009 | 0.450 | 0.675 | 0.010 | 0.299 | 0.020 | 0.159 | 0.010 | 0.527 | 0.030 | 0.009 | 0.043 | 0.308 | 0.006 | 0.242 | 0.299 | 0.586 | 0.054 | 0.000 |  |
| CpP3001 | 0.000 | 0.004 | 0.000 | 0.002 | 0.002 | 0.004 | 0.045 | 0.000 | 0.096 | 0.008 | 0.861 | 0.051 | 0.146 | 0.002 | 0.138 | 0.000 | 0.110 | 1.000 | 0.396 | 0.000 | 0.007 | 0.000 |

**S6 Table.** **Pairwise differentiation of linkage disequilibrium among Siamese crocodile (*Crocodylus siamensis*) individuals based on 22 microsatellite loci.** The number indicates *P* values, with 110 permutations.
