# Supplementary material for: High genetic diversity and demographic history of captive Siamese and Saltwater crocodiles suggest the first step toward the establishment of a breeding and reintroduction program in Thailand
Source: PLoS One. 2017 Sep 27;12(9):e0184526. doi: 10.1371/journal.pone.0184526 (PMC5617146; doi:10.1371/journal.pone.0184526)
Supplement: S7 Table — The number indicates P values, with 110 permutations. (DOCX) [file pone.0184526.s008.docx]

**S7 Table.** **Pairwise differentiation of linkage disequilibrium among Saltwater crocodile (*Crocodylus porosus*) individuals based on 22 microsatellite loci.** The number indicates *P* values, with 110 permutations.

| Locus | CpP208 | CpP501 | CpP1002 | CpP209 | CpP214 | CpP1308 | CpP203 | CpP2206 | CpP4004 | CpP3303 | CpF509 | CpP4501 | CpP1201 | CpP3004 | CpP3313 | CpP3508 | CpP1409 | CpP3008 | CpP2904 | CpP2504 | CpP3219 | CpP3001 |
| --- | --- | --- | --- | --- | --- | --- | --- | --- | --- | --- | --- | --- | --- | --- | --- | --- | --- | --- | --- | --- | --- | --- |
| CpP208 | 0.000 |  |  |  |  |  |  |  |  |  |  |  |  |  |  |  |  |  |  |  |  |  |
| CpP501 | 0.000 | 0.000 |  |  |  |  |  |  |  |  |  |  |  |  |  |  |  |  |  |  |  |  |
| CpP1002 | 0.016 | 0.000 | 0.000 |  |  |  |  |  |  |  |  |  |  |  |  |  |  |  |  |  |  |  |
| CpP209 | 0.008 | 0.000 | 0.000 | 0.000 |  |  |  |  |  |  |  |  |  |  |  |  |  |  |  |  |  |  |
| CpP214 | 0.144 | 0.137 | 0.000 | 0.000 | 0.000 |  |  |  |  |  |  |  |  |  |  |  |  |  |  |  |  |  |
| CpP1308 | 0.242 | 0.002 | 0.015 | 0.002 | 0.366 | 0.000 |  |  |  |  |  |  |  |  |  |  |  |  |  |  |  |  |
| CpP203 | 0.039 | 0.000 | 0.002 | 0.001 | 0.002 | 0.174 | 0.000 |  |  |  |  |  |  |  |  |  |  |  |  |  |  |  |
| CpP2206 | 0.006 | 0.001 | 0.000 | 0.001 | 0.015 | 0.252 | 0.024 | 0.000 |  |  |  |  |  |  |  |  |  |  |  |  |  |  |
| CpP4004 | 0.032 | 0.000 | 0.000 | 0.000 | 0.007 | 0.031 | 0.000 | 0.072 | 0.000 |  |  |  |  |  |  |  |  |  |  |  |  |  |
| CpP3303 | 0.923 | 0.022 | 0.000 | 0.000 | 0.054 | 0.209 | 0.021 | 0.001 | 0.226 | 0.000 |  |  |  |  |  |  |  |  |  |  |  |  |
| CpF509 | 0.004 | 0.003 | 0.000 | 0.000 | 0.000 | 0.179 | 0.236 | 0.001 | 0.211 | 0.000 | 0.000 |  |  |  |  |  |  |  |  |  |  |  |
| CpP4501 | 0.084 | 0.083 | 0.040 | 0.189 | 0.155 | 0.836 | 0.100 | 0.003 | 0.374 | 0.820 | 0.083 | 0.000 |  |  |  |  |  |  |  |  |  |  |
| CpP1201 | 0.000 | 0.000 | 0.000 | 0.000 | 0.006 | 0.014 | 0.017 | 0.009 | 0.005 | 0.000 | 0.000 | 0.225 | 0.000 |  |  |  |  |  |  |  |  |  |
| CpP3004 | 0.231 | 0.001 | 0.011 | 0.001 | 0.004 | 0.234 | 0.046 | 0.005 | 0.034 | 0.041 | 0.006 | 0.414 | 0.004 | 0.000 |  |  |  |  |  |  |  |  |
| CpP3313 | 0.057 | 0.230 | 0.045 | 0.939 | 0.019 | 0.849 | 0.314 | 0.011 | 0.406 | 0.058 | 0.064 | 0.118 | 0.481 | 0.001 | 0.000 |  |  |  |  |  |  |  |
| CpP3508 | 0.037 | 0.136 | 0.002 | 0.159 | 0.002 | 0.111 | 0.022 | 0.006 | 0.007 | 0.459 | 0.358 | 0.618 | 0.560 | 0.701 | 0.010 | 0.000 |  |  |  |  |  |  |
| CpP1409 | 0.019 | 0.007 | 0.019 | 0.000 | 0.060 | 0.000 | 0.012 | 0.003 | 0.090 | 0.004 | 0.001 | 0.120 | 0.000 | 0.006 | 0.420 | 0.101 | 0.000 |  |  |  |  |  |
| CpP3008 | 0.020 | 0.014 | 0.000 | 0.000 | 0.000 | 0.056 | 0.000 | 0.000 | 0.001 | 0.000 | 0.000 | 0.052 | 0.000 | 0.048 | 0.184 | 0.004 | 0.000 | 0.000 |  |  |  |  |
| CpP2904 | 0.017 | 0.236 | 0.137 | 0.003 | 0.095 | 0.350 | 0.329 | 0.001 | 0.966 | 0.111 | 0.002 | 0.007 | 0.014 | 0.132 | 0.063 | 0.481 | 0.000 | 0.008 | 0.000 |  |  |  |
| CpP2504 | 0.000 | 0.000 | 0.001 | 0.000 | 0.077 | 0.029 | 0.103 | 0.002 | 0.046 | 0.017 | 0.008 | 0.156 | 0.000 | 0.098 | 0.169 | 0.009 | 0.000 | 0.008 | 0.096 | 0.000 |  |  |
| CpP3219 | 0.002 | 0.011 | 0.008 | 0.010 | 0.006 | 0.048 | 0.038 | 0.000 | 0.082 | 0.055 | 0.004 | 0.101 | 0.002 | 0.004 | 0.103 | 0.028 | 0.000 | 0.002 | 0.005 | 0.000 | 0.000 |  |
| CpP3001 | 0.096 | 0.070 | 0.125 | 0.632 | 0.136 | 0.141 | 0.120 | 0.100 | 0.085 | 1.000 | 0.047 | 0.306 | 0.865 | 0.584 | 0.061 | 0.012 | 0.089 | 0.095 | 0.571 | 0.061 | 0.100 | 0.000 |
