# Supplementary material for: High genetic diversity and demographic history of captive Siamese and Saltwater crocodiles suggest the first step toward the establishment of a breeding and reintroduction program in Thailand
Source: PLoS One. 2017 Sep 27;12(9):e0184526. doi: 10.1371/journal.pone.0184526 (PMC5617146; doi:10.1371/journal.pone.0184526)
Supplement: S8 Table — Detailed information for all crocodile individuals is presented in S1 Table. (DOCX) [file pone.0184526.s009.docx]

**S8 Table.** **Comparison of observed and expected heterozygosity of the Siamese crocodile (*Crocodylus siamensis*) and Saltwater crocodile (*C. porosus*) based on 22 microsatellite loci in each captive/wild population.** Detailed information for all crocodile individuals is presented in S1 Table.

| Species | Captivity/Wild | H_o_ | H_e_ |
| --- | --- | --- | --- |
|  |  |  |  |
| *Crocodylus siamensis* | # 1 | 0.508±0.335 | 0.692±0.199 |
|  | # 2 | 0.627±0.331 | 0.627±0.206 |
|  | # 3 | 0.618±0.332 | 0.641±0.203 |
|  | # 4 | 0.611±0.287 | 0.639±0.232 |
|  | # 5 | 0.597±0.229 | 0.708±0.180 |
|  | # 6 | 0.688±0.335 | 0.708±0.196 |
|  | # 7 | 0.574±0.358 | 0.630±0.177 |
|  | # 8 | 0.683±0.359 | 0.648±0.162 |
|  | # 9 | 0.703±0.306 | 0.670±0.141 |
|  | # 10 | 0.734±0.347 | 0.621±0.173 |
|  | # 11 | 0.563±0.338 | 0.604±0.203 |
|  | # 12 | 0.717±0.281 | 0.610±0.140 |
|  | Wild # B | 0.663±0.280 | 0.611±0.212 |
| *Crocodylus porosus* | # 2 | 0.867±0.248 | 0.607±0.170 |
|  | # 3 | 0.797±0.245 | 0.665±0.137 |
|  | # 5 | 1.000±0.000 | 1.000±0.000 |
|  | # 6 | 0.650±0.249 | 0.702±0.157 |
|  | # 9 | 0.638±0.263 | 0.713±0.168 |

Column headings are: observed heterozygosity (H_o_); expected heterozygosity (H_e_).
