# Supplementary material for: High genetic diversity and demographic history of captive Siamese and Saltwater crocodiles suggest the first step toward the establishment of a breeding and reintroduction program in Thailand
Source: PLoS One. 2017 Sep 27;12(9):e0184526. doi: 10.1371/journal.pone.0184526 (PMC5617146; doi:10.1371/journal.pone.0184526)
Supplement: S9 Table — The number indicates P values, with 110 permutations. Detailed information for all crocodile individuals is presented in S1 Table. (DOCX) [file pone.0184526.s010.docx]

**S9 Table.** **Pairwise genetic differentiation (*F*_ST_) between Siamese crocodile (*Crocodylus siamensis*) captive/wild populations based on 22 microsatellite loci.** The number indicates *P* values, with 110 permutations. Detailed information for all crocodile individuals is presented in S1 Table.

| F_ST_ | # 1 | # 2 | # 3 | # 4 | # 5 | # 6 | # 7 | # 8 | # 9 | # 10 | # 11 | # 12 | Wild # B |
| --- | --- | --- | --- | --- | --- | --- | --- | --- | --- | --- | --- | --- | --- |
| # 1 | 0.000 |  |  |  |  |  |  |  |  |  |  |  |  |
| # 2 | 0.108 | 0.000 |  |  |  |  |  |  |  |  |  |  |  |
| # 3 | 0.051 | 0.144 | 0.000 |  |  |  |  |  |  |  |  |  |  |
| # 4 | 0.097 | 0.136 | 0.109 | 0.000 |  |  |  |  |  |  |  |  |  |
| # 5 | 0.077 | 0.129 | 0.115 | 0.073 | 0.000 |  |  |  |  |  |  |  |  |
| # 6 | 0.089 | 0.115 | 0.156 | 0.129 | 0.112 | 0.000 |  |  |  |  |  |  |  |
| # 7 | 0.102 | 0.130 | 0.166 | 0.150 | 0.119 | 0.100 | 0.000 |  |  |  |  |  |  |
| # 8 | 0.149 | 0.155 | 0.123 | 0.148 | 0.164 | 0.197 | 0.126 | 0.000 |  |  |  |  |  |
| # 9 | 0.141 | 0.130 | 0.158 | 0.119 | 0.137 | 0.091 | 0.105 | 0.105 | 0.000 |  |  |  |  |
| # 10 | 0.140 | 0.129 | 0.209 | 0.157 | 0.145 | 0.101 | 0.127 | 0.167 | 0.076 | 0.000 |  |  |  |
| # 11 | 0.141 | 0.187 | 0.240 | 0.188 | 0.147 | 0.130 | 0.106 | 0.210 | 0.142 | 0.122 | 0.000 |  |  |
| # 12 | 0.152 | 0.154 | 0.199 | 0.191 | 0.148 | 0.158 | 0.172 | 0.221 | 0.140 | 0.137 | 0.137 | 0.000 |  |
| Wild # B | 0.120 | 0.123 | 0.183 | 0.121 | 0.125 | 0.087 | 0.150 | 0.222 | 0.131 | 0.131 | 0.194 | 0.154 | 0.000 |
