# Supplementary material for: High genetic diversity and demographic history of captive Siamese and Saltwater crocodiles suggest the first step toward the establishment of a breeding and reintroduction program in Thailand
Source: PLoS One. 2017 Sep 27;12(9):e0184526. doi: 10.1371/journal.pone.0184526 (PMC5617146; doi:10.1371/journal.pone.0184526)
Supplement: S10 Table — The number indicates P values, with 110 permutations. Detailed information for all crocodile individuals is presented in S1 Table. (DOCX) [file pone.0184526.s011.docx]

**S10 Table.** **Pairwise genetic differentiation (*F*_ST_) between Saltwater crocodile (*Crocodylus porosus*) captive populations based on 22 microsatellite loci.** The number indicates *P* values, with 110 permutations. Detailed information for all crocodile individuals is presented in S1 Table.

| F_ST_ | # 2 | # 3 | # 5 | # 6 | # 9 |
| --- | --- | --- | --- | --- | --- |
| # 2 | 0.000 |  |  |  |  |
| # 3 | 0.356 | 0.000 |  |  |  |
| # 5 | 0.435 | 0.362 | 0.000 |  |  |
| # 6 | 0.297 | 0.202 | 0.270 | 0.000 |  |
| # 9 | 0.271 | 0.200 | 0.269 | 0.119 | 0.000 |
