# Supplementary material for: High genetic diversity and demographic history of captive Siamese and Saltwater crocodiles suggest the first step toward the establishment of a breeding and reintroduction program in Thailand
Source: PLoS One. 2017 Sep 27;12(9):e0184526. doi: 10.1371/journal.pone.0184526 (PMC5617146; doi:10.1371/journal.pone.0184526)
Supplement: S11 Table — Detailed information for all crocodile individuals is presented in S1 Table. (DOCX) [file pone.0184526.s012.docx]

**S11 Table.** **Test for genetic bottlenecks in the Siamese crocodile (*Crocodylus siamensis*) and Saltwater crocodile (*C. porosus*) using BOTTLENECK version 1.2.02 and calculation of the *M* ratio using Arlequin 3.5.2.2 for all populations.** Detailed information for all crocodile individuals is presented in S1 Table.

| Species | Captivity/Wild | Wilcoxon's sign-rank test | | Mode-shift test | *M* ratio |
| --- | --- | --- | --- | --- | --- |
|  |  | SMM | TPM |  |  |
| *Crocodylus siamensis* | # 1 | 0.446 | 0.354 | L-shaped mode | 0.215 |
|  | # 2 | 0.339 | 0.339 | shifted mode | 0.218 |
|  | # 3 | 0.463 | 0.463 | shifted mode | 0.281 |
|  | # 4 | N/A | N/A | N/A | 0.235 |
|  | # 5 | 0.383 | 0.351 | shifted mode | 0.252 |
|  | # 6 | 0.019 | 0.014 | shifted mode | 0.265 |
|  | # 7 | 0.517 | 0.500 | shifted mode | 0.238 |
|  | # 8 | 0.000 | 0.000 | shifted mode | 0.324 |
|  | # 9 | 0.106 | 0.106 | shifted mode | 0.266 |
|  | # 10 | 0.106 | 0.072 | shifted mode | 0.279 |
|  | # 11 | 0.371 | 0.334 | shifted mode | 0.291 |
|  | # 12 | 0.015 | 0.011 | shifted mode | 0.275 |
|  | Wild # B | 0.490 | 0.510 | shifted mode | 0.247 |
| *Crocodylus porosus* | # 2 | 0.001 | 0.001 | shifted mode | 0.250 |
|  | # 3 | 0.000 | 0.000 | shifted mode | 0.268 |
|  | # 5 | N/A | N/A | N/A | 0.209 |
|  | # 6 | 0.002 | 0.001 | shifted mode | 0.306 |
|  | # 9 | 0.522 | 0.522 | shifted mode | 0.301 |

“N/A”: Not Available.
