# Supplementary material for: High genetic diversity and demographic history of captive Siamese and Saltwater crocodiles suggest the first step toward the establishment of a breeding and reintroduction program in Thailand
Source: PLoS One. 2017 Sep 27;12(9):e0184526. doi: 10.1371/journal.pone.0184526 (PMC5617146; doi:10.1371/journal.pone.0184526)
Supplement: S12 Table — Detailed information for all crocodile individuals is presented in S1 Table. (DOCX) [file pone.0184526.s013.docx]

**S12 Table.** **Pairwise genetic relatedness (*r*) for all 69 crocodile individuals.** Detailed information for all crocodile individuals is presented in S1 Table.

| Individuals pairwise | Pairwise genetic relatedness (*r*) |
| --- | --- |
| CSI 01-CSI 01 | 0.000 |
| CSI 01-CSI 02 | 0.091 |
| CSI 01-CSI 03 | 0.054 |
| CSI 01-CSI 04 | 0.024 |
| CSI 01-CSI 05 | -0.007 |
| CSI 01-CSI 06 | -0.021 |
| CSI 01-CSI 07 | 0.005 |
| CSI 01-CSI 08 | -0.025 |
| CSI 01-CSI 09 | -0.012 |
| CSI 01-CSI 10 | 0.070 |
| CSI 01-CSI 11 | 0.007 |
| CSI 01-CSI 12 | 0.123 |
| CSI 01-CSI 13 | 0.064 |
| CSI 01-CSI 14 | 0.042 |
| CSI 01-CSI 15 | 0.053 |
| CSI 01-CSI 16 | 0.030 |
| CSI 01-CSI 17 | 0.017 |
| CSI 01-CSI 18 | 0.007 |
| CSI 01-CSI 19 | 0.037 |
| CSI 01-CSI 20 | 0.009 |
| CSI 01-CSI 21 | 0.037 |
| CSI 01-CSI 22 | 0.015 |
| CSI 01-CSI 23 | -0.001 |
| CSI 01-CSI 24 | 0.016 |
| CSI 01-CSI 25 | -0.030 |
| CSI 01-CSI 26 | -0.020 |
| CSI 01-CSI 27 | 0.008 |
| CSI 01-CSI 28 | -0.002 |
| CSI 01-CSI 29 | 0.019 |
| CSI 01-CSI 30 | 0.019 |
| CSI 01-CSI 31 | 0.011 |
| CSI 01-CSI 32 | 0.019 |
| CSI 01-CSI 33 | -0.025 |
| CSI 01-CSI 34 | -0.013 |
| CSI 01-CSI 35 | 0.023 |
| CSI 01-CSI 36 | 0.002 |
| CSI 01-CSI 37 | -0.015 |
| CSI 01-CSI 38 | -0.024 |
| CSI 01-CSI 39 | -0.015 |
| CSI 01-CSI 40 | -0.015 |
| CSI 01-CSI 41 | -0.019 |
| CSI 01-CSI 42 | -0.004 |
| CSI 01-CSI 43 | -0.007 |
| CSI 01-CSI 44 | -0.018 |
| CSI 01-CSI 45 | 0.034 |
| CSI 01-CSI 46 | 0.027 |
| CSI 01-CSI 47 | -0.007 |
| CSI 01-CSI 48 | 0.026 |
| CSI 01-CSI 49 | -0.005 |
| CSI 01-CSI 50 | -0.005 |
| CSI 01-CSI 51 | -0.008 |
| CSI 01-CSI 52 | -0.011 |
| CSI 01-CPO 01 | -0.075 |
| CSI 01-CPO 02 | -0.075 |
| CSI 01-CPO 03 | -0.072 |
| CSI 01-CPO 04 | -0.072 |
| CSI 01-CPO 05 | -0.067 |
| CSI 01-CPO 06 | -0.075 |
| CSI 01-CPO 07 | -0.067 |
| CSI 01-CPO 08 | -0.067 |
| CSI 01-CPO 09 | -0.036 |
| CSI 01-CPO 10 | -0.079 |
| CSI 01-CPO 11 | -0.075 |
| CSI 01-CPO 12 | -0.073 |
| CSI 01-CPO 13 | -0.077 |
| CSI 01-CPO 14 | -0.072 |
| CSI 01-CPO 15 | -0.075 |
| CSI 01-CPO 16 | -0.067 |
| CSI 01-CPO 17 | -0.070 |
| CSI 02-CSI 02 | 0.000 |
| CSI 02-CSI 03 | 0.070 |
| CSI 02-CSI 04 | 0.173 |
| CSI 02-CSI 05 | -0.020 |
| CSI 02-CSI 06 | -0.012 |
| CSI 02-CSI 07 | 0.022 |
| CSI 02-CSI 08 | -0.031 |
| CSI 02-CSI 09 | -0.015 |
| CSI 02-CSI 10 | 0.085 |
| CSI 02-CSI 11 | 0.059 |
| CSI 02-CSI 12 | 0.019 |
| CSI 02-CSI 13 | 0.061 |
| CSI 02-CSI 14 | 0.039 |
| CSI 02-CSI 15 | 0.032 |
| CSI 02-CSI 16 | 0.000 |
| CSI 02-CSI 17 | -0.020 |
| CSI 02-CSI 18 | 0.000 |
| CSI 02-CSI 19 | 0.014 |
| CSI 02-CSI 20 | 0.023 |
| CSI 02-CSI 21 | -0.011 |
| CSI 02-CSI 22 | 0.001 |
| CSI 02-CSI 23 | 0.033 |
| CSI 02-CSI 24 | -0.019 |
| CSI 02-CSI 25 | 0.007 |
| CSI 02-CSI 26 | -0.003 |
| CSI 02-CSI 27 | 0.033 |
| CSI 02-CSI 28 | -0.009 |
| CSI 02-CSI 29 | -0.016 |
| CSI 02-CSI 30 | -0.016 |
| CSI 02-CSI 31 | -0.006 |
| CSI 02-CSI 32 | 0.035 |
| CSI 02-CSI 33 | -0.008 |
| CSI 02-CSI 34 | -0.018 |
| CSI 02-CSI 35 | -0.028 |
| CSI 02-CSI 36 | -0.034 |
| CSI 02-CSI 37 | 0.097 |
| CSI 02-CSI 38 | -0.013 |
| CSI 02-CSI 39 | -0.028 |
| CSI 02-CSI 40 | -0.028 |
| CSI 02-CSI 41 | -0.014 |
| CSI 02-CSI 42 | -0.007 |
| CSI 02-CSI 43 | -0.018 |
| CSI 02-CSI 44 | -0.025 |
| CSI 02-CSI 45 | 0.005 |
| CSI 02-CSI 46 | -0.017 |
| CSI 02-CSI 47 | -0.020 |
| CSI 02-CSI 48 | -0.023 |
| CSI 02-CSI 49 | 0.008 |
| CSI 02-CSI 50 | -0.004 |
| CSI 02-CSI 51 | -0.007 |
| CSI 02-CSI 52 | 0.001 |
| CSI 02-CPO 01 | -0.049 |
| CSI 02-CPO 02 | -0.049 |
| CSI 02-CPO 03 | -0.048 |
| CSI 02-CPO 04 | -0.057 |
| CSI 02-CPO 05 | -0.063 |
| CSI 02-CPO 06 | -0.071 |
| CSI 02-CPO 07 | -0.067 |
| CSI 02-CPO 08 | -0.067 |
| CSI 02-CPO 09 | -0.012 |
| CSI 02-CPO 10 | -0.079 |
| CSI 02-CPO 11 | -0.075 |
| CSI 02-CPO 12 | -0.064 |
| CSI 02-CPO 13 | -0.079 |
| CSI 02-CPO 14 | -0.073 |
| CSI 02-CPO 15 | -0.075 |
| CSI 02-CPO 16 | -0.061 |
| CSI 02-CPO 17 | -0.062 |
| CSI 03-CSI 03 | 0.000 |
| CSI 03-CSI 04 | 0.085 |
| CSI 03-CSI 05 | -0.049 |
| CSI 03-CSI 06 | -0.031 |
| CSI 03-CSI 07 | 0.016 |
| CSI 03-CSI 08 | -0.021 |
| CSI 03-CSI 09 | 0.001 |
| CSI 03-CSI 10 | 0.126 |
| CSI 03-CSI 11 | 0.045 |
| CSI 03-CSI 12 | 0.070 |
| CSI 03-CSI 13 | 0.102 |
| CSI 03-CSI 14 | -0.010 |
| CSI 03-CSI 15 | -0.014 |
| CSI 03-CSI 16 | -0.004 |
| CSI 03-CSI 17 | 0.015 |
| CSI 03-CSI 18 | -0.022 |
| CSI 03-CSI 19 | 0.042 |
| CSI 03-CSI 20 | -0.016 |
| CSI 03-CSI 21 | -0.019 |
| CSI 03-CSI 22 | -0.002 |
| CSI 03-CSI 23 | 0.006 |
| CSI 03-CSI 24 | 0.007 |
| CSI 03-CSI 25 | 0.017 |
| CSI 03-CSI 26 | -0.012 |
| CSI 03-CSI 27 | -0.008 |
| CSI 03-CSI 28 | -0.023 |
| CSI 03-CSI 29 | 0.008 |
| CSI 03-CSI 30 | 0.047 |
| CSI 03-CSI 31 | 0.013 |
| CSI 03-CSI 32 | 0.013 |
| CSI 03-CSI 33 | 0.004 |
| CSI 03-CSI 34 | 0.001 |
| CSI 03-CSI 35 | -0.014 |
| CSI 03-CSI 36 | -0.026 |
| CSI 03-CSI 37 | -0.014 |
| CSI 03-CSI 38 | 0.029 |
| CSI 03-CSI 39 | 0.001 |
| CSI 03-CSI 40 | 0.017 |
| CSI 03-CSI 41 | -0.007 |
| CSI 03-CSI 42 | -0.014 |
| CSI 03-CSI 43 | -0.017 |
| CSI 03-CSI 44 | -0.026 |
| CSI 03-CSI 45 | -0.013 |
| CSI 03-CSI 46 | -0.004 |
| CSI 03-CSI 47 | -0.003 |
| CSI 03-CSI 48 | -0.006 |
| CSI 03-CSI 49 | 0.019 |
| CSI 03-CSI 50 | 0.003 |
| CSI 03-CSI 51 | 0.007 |
| CSI 03-CSI 52 | 0.022 |
| CSI 03-CPO 01 | -0.066 |
| CSI 03-CPO 02 | -0.066 |
| CSI 03-CPO 03 | -0.063 |
| CSI 03-CPO 04 | -0.069 |
| CSI 03-CPO 05 | -0.072 |
| CSI 03-CPO 06 | -0.076 |
| CSI 03-CPO 07 | -0.069 |
| CSI 03-CPO 08 | -0.069 |
| CSI 03-CPO 09 | -0.042 |
| CSI 03-CPO 10 | -0.072 |
| CSI 03-CPO 11 | -0.077 |
| CSI 03-CPO 12 | -0.069 |
| CSI 03-CPO 13 | -0.064 |
| CSI 03-CPO 14 | -0.050 |
| CSI 03-CPO 15 | -0.066 |
| CSI 03-CPO 16 | -0.059 |
| CSI 03-CPO 17 | -0.066 |
| CSI 04-CSI 04 | 0.000 |
| CSI 04-CSI 05 | -0.053 |
| CSI 04-CSI 06 | -0.054 |
| CSI 04-CSI 07 | -0.022 |
| CSI 04-CSI 08 | -0.008 |
| CSI 04-CSI 09 | 0.002 |
| CSI 04-CSI 10 | 0.054 |
| CSI 04-CSI 11 | 0.169 |
| CSI 04-CSI 12 | 0.051 |
| CSI 04-CSI 13 | 0.035 |
| CSI 04-CSI 14 | -0.016 |
| CSI 04-CSI 15 | 0.050 |
| CSI 04-CSI 16 | -0.016 |
| CSI 04-CSI 17 | 0.020 |
| CSI 04-CSI 18 | -0.007 |
| CSI 04-CSI 19 | 0.002 |
| CSI 04-CSI 20 | -0.030 |
| CSI 04-CSI 21 | -0.015 |
| CSI 04-CSI 22 | 0.021 |
| CSI 04-CSI 23 | 0.057 |
| CSI 04-CSI 24 | 0.009 |
| CSI 04-CSI 25 | 0.021 |
| CSI 04-CSI 26 | -0.019 |
| CSI 04-CSI 27 | -0.011 |
| CSI 04-CSI 28 | -0.030 |
| CSI 04-CSI 29 | -0.037 |
| CSI 04-CSI 30 | -0.033 |
| CSI 04-CSI 31 | -0.012 |
| CSI 04-CSI 32 | -0.012 |
| CSI 04-CSI 33 | 0.022 |
| CSI 04-CSI 34 | 0.009 |
| CSI 04-CSI 35 | 0.005 |
| CSI 04-CSI 36 | -0.014 |
| CSI 04-CSI 37 | -0.016 |
| CSI 04-CSI 38 | 0.011 |
| CSI 04-CSI 39 | 0.032 |
| CSI 04-CSI 40 | -0.016 |
| CSI 04-CSI 41 | 0.051 |
| CSI 04-CSI 42 | 0.002 |
| CSI 04-CSI 43 | 0.033 |
| CSI 04-CSI 44 | 0.017 |
| CSI 04-CSI 45 | -0.019 |
| CSI 04-CSI 46 | 0.018 |
| CSI 04-CSI 47 | 0.019 |
| CSI 04-CSI 48 | -0.010 |
| CSI 04-CSI 49 | 0.006 |
| CSI 04-CSI 50 | -0.018 |
| CSI 04-CSI 51 | -0.011 |
| CSI 04-CSI 52 | 0.001 |
| CSI 04-CPO 01 | -0.050 |
| CSI 04-CPO 02 | -0.050 |
| CSI 04-CPO 03 | -0.058 |
| CSI 04-CPO 04 | -0.057 |
| CSI 04-CPO 05 | -0.079 |
| CSI 04-CPO 06 | -0.079 |
| CSI 04-CPO 07 | -0.074 |
| CSI 04-CPO 08 | -0.074 |
| CSI 04-CPO 09 | 0.014 |
| CSI 04-CPO 10 | -0.060 |
| CSI 04-CPO 11 | -0.074 |
| CSI 04-CPO 12 | -0.057 |
| CSI 04-CPO 13 | -0.047 |
| CSI 04-CPO 14 | -0.056 |
| CSI 04-CPO 15 | -0.056 |
| CSI 04-CPO 16 | -0.077 |
| CSI 04-CPO 17 | -0.050 |
| CSI 05-CSI 05 | 0.000 |
| CSI 05-CSI 06 | 0.119 |
| CSI 05-CSI 07 | -0.044 |
| CSI 05-CSI 08 | -0.039 |
| CSI 05-CSI 09 | -0.030 |
| CSI 05-CSI 10 | -0.039 |
| CSI 05-CSI 11 | -0.061 |
| CSI 05-CSI 12 | -0.016 |
| CSI 05-CSI 13 | -0.033 |
| CSI 05-CSI 14 | -0.045 |
| CSI 05-CSI 15 | -0.031 |
| CSI 05-CSI 16 | -0.045 |
| CSI 05-CSI 17 | -0.055 |
| CSI 05-CSI 18 | 0.001 |
| CSI 05-CSI 19 | -0.027 |
| CSI 05-CSI 20 | 0.003 |
| CSI 05-CSI 21 | 0.074 |
| CSI 05-CSI 22 | -0.043 |
| CSI 05-CSI 23 | -0.040 |
| CSI 05-CSI 24 | -0.042 |
| CSI 05-CSI 25 | -0.046 |
| CSI 05-CSI 26 | -0.052 |
| CSI 05-CSI 27 | -0.021 |
| CSI 05-CSI 28 | -0.012 |
| CSI 05-CSI 29 | -0.040 |
| CSI 05-CSI 30 | -0.037 |
| CSI 05-CSI 31 | -0.054 |
| CSI 05-CSI 32 | 0.000 |
| CSI 05-CSI 33 | -0.051 |
| CSI 05-CSI 34 | -0.045 |
| CSI 05-CSI 35 | 0.011 |
| CSI 05-CSI 36 | -0.020 |
| CSI 05-CSI 37 | -0.029 |
| CSI 05-CSI 38 | -0.038 |
| CSI 05-CSI 39 | -0.034 |
| CSI 05-CSI 40 | -0.037 |
| CSI 05-CSI 41 | -0.052 |
| CSI 05-CSI 42 | 0.012 |
| CSI 05-CSI 43 | -0.042 |
| CSI 05-CSI 44 | -0.018 |
| CSI 05-CSI 45 | -0.019 |
| CSI 05-CSI 46 | -0.013 |
| CSI 05-CSI 47 | -0.039 |
| CSI 05-CSI 48 | 0.002 |
| CSI 05-CSI 49 | -0.031 |
| CSI 05-CSI 50 | -0.049 |
| CSI 05-CSI 51 | -0.041 |
| CSI 05-CSI 52 | -0.025 |
| CSI 05-CPO 01 | -0.006 |
| CSI 05-CPO 02 | -0.006 |
| CSI 05-CPO 03 | -0.014 |
| CSI 05-CPO 04 | -0.024 |
| CSI 05-CPO 05 | -0.020 |
| CSI 05-CPO 06 | -0.012 |
| CSI 05-CPO 07 | 0.011 |
| CSI 05-CPO 08 | 0.011 |
| CSI 05-CPO 09 | -0.052 |
| CSI 05-CPO 10 | -0.029 |
| CSI 05-CPO 11 | -0.022 |
| CSI 05-CPO 12 | -0.023 |
| CSI 05-CPO 13 | -0.005 |
| CSI 05-CPO 14 | -0.016 |
| CSI 05-CPO 15 | -0.034 |
| CSI 05-CPO 16 | 0.006 |
| CSI 05-CPO 17 | -0.036 |
| CSI 06-CSI 06 | 0.000 |
| CSI 06-CSI 07 | -0.048 |
| CSI 06-CSI 08 | -0.059 |
| CSI 06-CSI 09 | -0.021 |
| CSI 06-CSI 10 | -0.038 |
| CSI 06-CSI 11 | -0.057 |
| CSI 06-CSI 12 | -0.051 |
| CSI 06-CSI 13 | -0.036 |
| CSI 06-CSI 14 | -0.052 |
| CSI 06-CSI 15 | -0.054 |
| CSI 06-CSI 16 | -0.050 |
| CSI 06-CSI 17 | -0.051 |
| CSI 06-CSI 18 | -0.044 |
| CSI 06-CSI 19 | -0.039 |
| CSI 06-CSI 20 | -0.022 |
| CSI 06-CSI 21 | -0.029 |
| CSI 06-CSI 22 | -0.032 |
| CSI 06-CSI 23 | -0.055 |
| CSI 06-CSI 24 | -0.037 |
| CSI 06-CSI 25 | -0.055 |
| CSI 06-CSI 26 | -0.032 |
| CSI 06-CSI 27 | -0.002 |
| CSI 06-CSI 28 | -0.009 |
| CSI 06-CSI 29 | -0.047 |
| CSI 06-CSI 30 | -0.039 |
| CSI 06-CSI 31 | 0.007 |
| CSI 06-CSI 32 | 0.048 |
| CSI 06-CSI 33 | -0.059 |
| CSI 06-CSI 34 | -0.018 |
| CSI 06-CSI 35 | -0.043 |
| CSI 06-CSI 36 | -0.065 |
| CSI 06-CSI 37 | -0.018 |
| CSI 06-CSI 38 | -0.055 |
| CSI 06-CSI 39 | -0.018 |
| CSI 06-CSI 40 | -0.022 |
| CSI 06-CSI 41 | -0.047 |
| CSI 06-CSI 42 | -0.037 |
| CSI 06-CSI 43 | -0.057 |
| CSI 06-CSI 44 | -0.022 |
| CSI 06-CSI 45 | -0.021 |
| CSI 06-CSI 46 | -0.030 |
| CSI 06-CSI 47 | -0.031 |
| CSI 06-CSI 48 | -0.016 |
| CSI 06-CSI 49 | -0.024 |
| CSI 06-CSI 50 | -0.045 |
| CSI 06-CSI 51 | -0.034 |
| CSI 06-CSI 52 | -0.036 |
| CSI 06-CPO 01 | 0.028 |
| CSI 06-CPO 02 | 0.028 |
| CSI 06-CPO 03 | -0.026 |
| CSI 06-CPO 04 | -0.022 |
| CSI 06-CPO 05 | 0.092 |
| CSI 06-CPO 06 | 0.095 |
| CSI 06-CPO 07 | 0.071 |
| CSI 06-CPO 08 | 0.050 |
| CSI 06-CPO 09 | -0.021 |
| CSI 06-CPO 10 | -0.021 |
| CSI 06-CPO 11 | -0.016 |
| CSI 06-CPO 12 | 0.040 |
| CSI 06-CPO 13 | 0.005 |
| CSI 06-CPO 14 | 0.145 |
| CSI 06-CPO 15 | 0.104 |
| CSI 06-CPO 16 | 0.139 |
| CSI 06-CPO 17 | 0.045 |
| CSI 07-CSI 07 | 0.000 |
| CSI 07-CSI 08 | 0.126 |
| CSI 07-CSI 09 | 0.191 |
| CSI 07-CSI 10 | 0.011 |
| CSI 07-CSI 11 | 0.060 |
| CSI 07-CSI 12 | -0.009 |
| CSI 07-CSI 13 | 0.011 |
| CSI 07-CSI 14 | -0.017 |
| CSI 07-CSI 15 | -0.011 |
| CSI 07-CSI 16 | 0.032 |
| CSI 07-CSI 17 | -0.028 |
| CSI 07-CSI 18 | -0.030 |
| CSI 07-CSI 19 | -0.012 |
| CSI 07-CSI 20 | 0.033 |
| CSI 07-CSI 21 | 0.052 |
| CSI 07-CSI 22 | -0.006 |
| CSI 07-CSI 23 | -0.007 |
| CSI 07-CSI 24 | 0.018 |
| CSI 07-CSI 25 | -0.005 |
| CSI 07-CSI 26 | 0.000 |
| CSI 07-CSI 27 | 0.012 |
| CSI 07-CSI 28 | 0.019 |
| CSI 07-CSI 29 | 0.046 |
| CSI 07-CSI 30 | 0.057 |
| CSI 07-CSI 31 | 0.006 |
| CSI 07-CSI 32 | 0.011 |
| CSI 07-CSI 33 | -0.007 |
| CSI 07-CSI 34 | -0.002 |
| CSI 07-CSI 35 | -0.002 |
| CSI 07-CSI 36 | -0.015 |
| CSI 07-CSI 37 | -0.005 |
| CSI 07-CSI 38 | 0.032 |
| CSI 07-CSI 39 | -0.010 |
| CSI 07-CSI 40 | 0.003 |
| CSI 07-CSI 41 | -0.001 |
| CSI 07-CSI 42 | -0.005 |
| CSI 07-CSI 43 | 0.021 |
| CSI 07-CSI 44 | -0.008 |
| CSI 07-CSI 45 | 0.018 |
| CSI 07-CSI 46 | 0.013 |
| CSI 07-CSI 47 | 0.010 |
| CSI 07-CSI 48 | 0.009 |
| CSI 07-CSI 49 | 0.014 |
| CSI 07-CSI 50 | 0.025 |
| CSI 07-CSI 51 | 0.020 |
| CSI 07-CSI 52 | 0.020 |
| CSI 07-CPO 01 | -0.064 |
| CSI 07-CPO 02 | -0.064 |
| CSI 07-CPO 03 | -0.044 |
| CSI 07-CPO 04 | -0.041 |
| CSI 07-CPO 05 | -0.069 |
| CSI 07-CPO 06 | -0.073 |
| CSI 07-CPO 07 | -0.062 |
| CSI 07-CPO 08 | -0.062 |
| CSI 07-CPO 09 | -0.060 |
| CSI 07-CPO 10 | -0.061 |
| CSI 07-CPO 11 | -0.070 |
| CSI 07-CPO 12 | -0.071 |
| CSI 07-CPO 13 | -0.057 |
| CSI 07-CPO 14 | -0.057 |
| CSI 07-CPO 15 | -0.057 |
| CSI 07-CPO 16 | -0.059 |
| CSI 07-CPO 17 | -0.049 |
| CSI 08-CSI 08 | 0.000 |
| CSI 08-CSI 09 | 0.121 |
| CSI 08-CSI 10 | -0.004 |
| CSI 08-CSI 11 | 0.028 |
| CSI 08-CSI 12 | 0.020 |
| CSI 08-CSI 13 | -0.007 |
| CSI 08-CSI 14 | -0.007 |
| CSI 08-CSI 15 | -0.014 |
| CSI 08-CSI 16 | 0.053 |
| CSI 08-CSI 17 | 0.000 |
| CSI 08-CSI 18 | -0.003 |
| CSI 08-CSI 19 | -0.028 |
| CSI 08-CSI 20 | 0.002 |
| CSI 08-CSI 21 | -0.001 |
| CSI 08-CSI 22 | -0.017 |
| CSI 08-CSI 23 | 0.021 |
| CSI 08-CSI 24 | -0.013 |
| CSI 08-CSI 25 | -0.024 |
| CSI 08-CSI 26 | -0.009 |
| CSI 08-CSI 27 | -0.021 |
| CSI 08-CSI 28 | 0.001 |
| CSI 08-CSI 29 | -0.029 |
| CSI 08-CSI 30 | 0.015 |
| CSI 08-CSI 31 | 0.040 |
| CSI 08-CSI 32 | 0.005 |
| CSI 08-CSI 33 | 0.030 |
| CSI 08-CSI 34 | -0.012 |
| CSI 08-CSI 35 | -0.017 |
| CSI 08-CSI 36 | 0.043 |
| CSI 08-CSI 37 | 0.011 |
| CSI 08-CSI 38 | -0.012 |
| CSI 08-CSI 39 | 0.018 |
| CSI 08-CSI 40 | -0.003 |
| CSI 08-CSI 41 | -0.012 |
| CSI 08-CSI 42 | -0.021 |
| CSI 08-CSI 43 | -0.016 |
| CSI 08-CSI 44 | 0.019 |
| CSI 08-CSI 45 | -0.003 |
| CSI 08-CSI 46 | -0.009 |
| CSI 08-CSI 47 | 0.044 |
| CSI 08-CSI 48 | -0.023 |
| CSI 08-CSI 49 | -0.001 |
| CSI 08-CSI 50 | 0.013 |
| CSI 08-CSI 51 | -0.012 |
| CSI 08-CSI 52 | -0.011 |
| CSI 08-CPO 01 | -0.035 |
| CSI 08-CPO 02 | -0.035 |
| CSI 08-CPO 03 | -0.042 |
| CSI 08-CPO 04 | -0.045 |
| CSI 08-CPO 05 | -0.049 |
| CSI 08-CPO 06 | -0.049 |
| CSI 08-CPO 07 | -0.068 |
| CSI 08-CPO 08 | -0.058 |
| CSI 08-CPO 09 | -0.029 |
| CSI 08-CPO 10 | 0.007 |
| CSI 08-CPO 11 | -0.062 |
| CSI 08-CPO 12 | -0.067 |
| CSI 08-CPO 13 | 0.034 |
| CSI 08-CPO 14 | -0.017 |
| CSI 08-CPO 15 | -0.061 |
| CSI 08-CPO 16 | -0.061 |
| CSI 08-CPO 17 | 0.006 |
| CSI 09-CSI 09 | 0.000 |
| CSI 09-CSI 10 | -0.008 |
| CSI 09-CSI 11 | 0.043 |
| CSI 09-CSI 12 | -0.034 |
| CSI 09-CSI 13 | -0.010 |
| CSI 09-CSI 14 | -0.006 |
| CSI 09-CSI 15 | -0.022 |
| CSI 09-CSI 16 | -0.003 |
| CSI 09-CSI 17 | -0.027 |
| CSI 09-CSI 18 | -0.023 |
| CSI 09-CSI 19 | -0.024 |
| CSI 09-CSI 20 | -0.032 |
| CSI 09-CSI 21 | 0.017 |
| CSI 09-CSI 22 | 0.011 |
| CSI 09-CSI 23 | -0.017 |
| CSI 09-CSI 24 | 0.028 |
| CSI 09-CSI 25 | -0.020 |
| CSI 09-CSI 26 | -0.015 |
| CSI 09-CSI 27 | -0.013 |
| CSI 09-CSI 28 | 0.008 |
| CSI 09-CSI 29 | -0.016 |
| CSI 09-CSI 30 | -0.005 |
| CSI 09-CSI 31 | -0.008 |
| CSI 09-CSI 32 | -0.008 |
| CSI 09-CSI 33 | -0.015 |
| CSI 09-CSI 34 | 0.011 |
| CSI 09-CSI 35 | -0.011 |
| CSI 09-CSI 36 | -0.011 |
| CSI 09-CSI 37 | 0.026 |
| CSI 09-CSI 38 | 0.007 |
| CSI 09-CSI 39 | 0.026 |
| CSI 09-CSI 40 | 0.018 |
| CSI 09-CSI 41 | -0.022 |
| CSI 09-CSI 42 | 0.010 |
| CSI 09-CSI 43 | 0.024 |
| CSI 09-CSI 44 | -0.006 |
| CSI 09-CSI 45 | 0.007 |
| CSI 09-CSI 46 | 0.046 |
| CSI 09-CSI 47 | 0.022 |
| CSI 09-CSI 48 | 0.003 |
| CSI 09-CSI 49 | 0.025 |
| CSI 09-CSI 50 | 0.009 |
| CSI 09-CSI 51 | 0.018 |
| CSI 09-CSI 52 | 0.017 |
| CSI 09-CPO 01 | -0.037 |
| CSI 09-CPO 02 | -0.037 |
| CSI 09-CPO 03 | -0.047 |
| CSI 09-CPO 04 | -0.048 |
| CSI 09-CPO 05 | -0.050 |
| CSI 09-CPO 06 | -0.054 |
| CSI 09-CPO 07 | -0.066 |
| CSI 09-CPO 08 | -0.066 |
| CSI 09-CPO 09 | -0.035 |
| CSI 09-CPO 10 | -0.051 |
| CSI 09-CPO 11 | -0.070 |
| CSI 09-CPO 12 | -0.067 |
| CSI 09-CPO 13 | -0.045 |
| CSI 09-CPO 14 | -0.023 |
| CSI 09-CPO 15 | -0.033 |
| CSI 09-CPO 16 | -0.050 |
| CSI 09-CPO 17 | -0.032 |
| CSI 10-CSI 10 | 0.000 |
| CSI 10-CSI 11 | 0.044 |
| CSI 10-CSI 12 | 0.038 |
| CSI 10-CSI 13 | 0.303 |
| CSI 10-CSI 14 | 0.010 |
| CSI 10-CSI 15 | -0.002 |
| CSI 10-CSI 16 | 0.014 |
| CSI 10-CSI 17 | 0.019 |
| CSI 10-CSI 18 | -0.020 |
| CSI 10-CSI 19 | -0.019 |
| CSI 10-CSI 20 | -0.003 |
| CSI 10-CSI 21 | -0.017 |
| CSI 10-CSI 22 | -0.017 |
| CSI 10-CSI 23 | 0.008 |
| CSI 10-CSI 24 | -0.003 |
| CSI 10-CSI 25 | 0.001 |
| CSI 10-CSI 26 | -0.005 |
| CSI 10-CSI 27 | -0.006 |
| CSI 10-CSI 28 | -0.022 |
| CSI 10-CSI 29 | 0.040 |
| CSI 10-CSI 30 | 0.044 |
| CSI 10-CSI 31 | 0.008 |
| CSI 10-CSI 32 | 0.016 |
| CSI 10-CSI 33 | 0.008 |
| CSI 10-CSI 34 | -0.016 |
| CSI 10-CSI 35 | 0.005 |
| CSI 10-CSI 36 | -0.008 |
| CSI 10-CSI 37 | -0.009 |
| CSI 10-CSI 38 | 0.005 |
| CSI 10-CSI 39 | -0.009 |
| CSI 10-CSI 40 | 0.016 |
| CSI 10-CSI 41 | -0.009 |
| CSI 10-CSI 42 | 0.014 |
| CSI 10-CSI 43 | -0.021 |
| CSI 10-CSI 44 | -0.005 |
| CSI 10-CSI 45 | -0.016 |
| CSI 10-CSI 46 | -0.001 |
| CSI 10-CSI 47 | 0.019 |
| CSI 10-CSI 48 | -0.005 |
| CSI 10-CSI 49 | 0.013 |
| CSI 10-CSI 50 | -0.010 |
| CSI 10-CSI 51 | 0.003 |
| CSI 10-CSI 52 | 0.004 |
| CSI 10-CPO 01 | -0.062 |
| CSI 10-CPO 02 | -0.062 |
| CSI 10-CPO 03 | -0.063 |
| CSI 10-CPO 04 | -0.066 |
| CSI 10-CPO 05 | -0.064 |
| CSI 10-CPO 06 | -0.068 |
| CSI 10-CPO 07 | -0.058 |
| CSI 10-CPO 08 | -0.058 |
| CSI 10-CPO 09 | -0.030 |
| CSI 10-CPO 10 | -0.062 |
| CSI 10-CPO 11 | -0.066 |
| CSI 10-CPO 12 | -0.061 |
| CSI 10-CPO 13 | -0.062 |
| CSI 10-CPO 14 | -0.058 |
| CSI 10-CPO 15 | -0.058 |
| CSI 10-CPO 16 | -0.052 |
| CSI 10-CPO 17 | -0.055 |
| CSI 11-CSI 11 | 0.000 |
| CSI 11-CSI 12 | 0.139 |
| CSI 11-CSI 13 | 0.024 |
| CSI 11-CSI 14 | 0.030 |
| CSI 11-CSI 15 | 0.004 |
| CSI 11-CSI 16 | 0.011 |
| CSI 11-CSI 17 | 0.004 |
| CSI 11-CSI 18 | 0.005 |
| CSI 11-CSI 19 | 0.076 |
| CSI 11-CSI 20 | 0.015 |
| CSI 11-CSI 21 | -0.033 |
| CSI 11-CSI 22 | -0.018 |
| CSI 11-CSI 23 | 0.046 |
| CSI 11-CSI 24 | -0.029 |
| CSI 11-CSI 25 | -0.001 |
| CSI 11-CSI 26 | -0.027 |
| CSI 11-CSI 27 | -0.026 |
| CSI 11-CSI 28 | -0.018 |
| CSI 11-CSI 29 | 0.004 |
| CSI 11-CSI 30 | 0.008 |
| CSI 11-CSI 31 | 0.005 |
| CSI 11-CSI 32 | 0.005 |
| CSI 11-CSI 33 | 0.005 |
| CSI 11-CSI 34 | 0.001 |
| CSI 11-CSI 35 | -0.010 |
| CSI 11-CSI 36 | -0.009 |
| CSI 11-CSI 37 | -0.015 |
| CSI 11-CSI 38 | -0.006 |
| CSI 11-CSI 39 | -0.015 |
| CSI 11-CSI 40 | -0.003 |
| CSI 11-CSI 41 | -0.034 |
| CSI 11-CSI 42 | -0.008 |
| CSI 11-CSI 43 | -0.039 |
| CSI 11-CSI 44 | 0.001 |
| CSI 11-CSI 45 | 0.013 |
| CSI 11-CSI 46 | 0.006 |
| CSI 11-CSI 47 | 0.015 |
| CSI 11-CSI 48 | 0.005 |
| CSI 11-CSI 49 | -0.010 |
| CSI 11-CSI 50 | 0.013 |
| CSI 11-CSI 51 | -0.015 |
| CSI 11-CSI 52 | -0.017 |
| CSI 11-CPO 01 | -0.054 |
| CSI 11-CPO 02 | -0.054 |
| CSI 11-CPO 03 | -0.040 |
| CSI 11-CPO 04 | -0.040 |
| CSI 11-CPO 05 | -0.073 |
| CSI 11-CPO 06 | -0.073 |
| CSI 11-CPO 07 | -0.073 |
| CSI 11-CPO 08 | -0.073 |
| CSI 11-CPO 09 | -0.026 |
| CSI 11-CPO 10 | -0.075 |
| CSI 11-CPO 11 | -0.073 |
| CSI 11-CPO 12 | -0.071 |
| CSI 11-CPO 13 | -0.073 |
| CSI 11-CPO 14 | -0.062 |
| CSI 11-CPO 15 | -0.073 |
| CSI 11-CPO 16 | -0.071 |
| CSI 11-CPO 17 | -0.048 |
| CSI 12-CSI 12 | 0.000 |
| CSI 12-CSI 13 | 0.036 |
| CSI 12-CSI 14 | 0.027 |
| CSI 12-CSI 15 | 0.028 |
| CSI 12-CSI 16 | -0.027 |
| CSI 12-CSI 17 | -0.001 |
| CSI 12-CSI 18 | 0.043 |
| CSI 12-CSI 19 | 0.119 |
| CSI 12-CSI 20 | 0.054 |
| CSI 12-CSI 21 | 0.005 |
| CSI 12-CSI 22 | -0.006 |
| CSI 12-CSI 23 | 0.040 |
| CSI 12-CSI 24 | -0.015 |
| CSI 12-CSI 25 | 0.016 |
| CSI 12-CSI 26 | -0.024 |
| CSI 12-CSI 27 | 0.004 |
| CSI 12-CSI 28 | 0.016 |
| CSI 12-CSI 29 | 0.037 |
| CSI 12-CSI 30 | 0.031 |
| CSI 12-CSI 31 | 0.011 |
| CSI 12-CSI 32 | -0.013 |
| CSI 12-CSI 33 | -0.025 |
| CSI 12-CSI 34 | 0.025 |
| CSI 12-CSI 35 | -0.004 |
| CSI 12-CSI 36 | 0.005 |
| CSI 12-CSI 37 | -0.022 |
| CSI 12-CSI 38 | -0.027 |
| CSI 12-CSI 39 | -0.022 |
| CSI 12-CSI 40 | -0.022 |
| CSI 12-CSI 41 | -0.027 |
| CSI 12-CSI 42 | -0.003 |
| CSI 12-CSI 43 | -0.035 |
| CSI 12-CSI 44 | 0.000 |
| CSI 12-CSI 45 | 0.023 |
| CSI 12-CSI 46 | 0.036 |
| CSI 12-CSI 47 | 0.014 |
| CSI 12-CSI 48 | 0.014 |
| CSI 12-CSI 49 | -0.019 |
| CSI 12-CSI 50 | 0.039 |
| CSI 12-CSI 51 | -0.040 |
| CSI 12-CSI 52 | -0.021 |
| CSI 12-CPO 01 | -0.073 |
| CSI 12-CPO 02 | -0.073 |
| CSI 12-CPO 03 | -0.065 |
| CSI 12-CPO 04 | -0.073 |
| CSI 12-CPO 05 | -0.071 |
| CSI 12-CPO 06 | -0.071 |
| CSI 12-CPO 07 | -0.075 |
| CSI 12-CPO 08 | -0.075 |
| CSI 12-CPO 09 | -0.033 |
| CSI 12-CPO 10 | -0.079 |
| CSI 12-CPO 11 | -0.075 |
| CSI 12-CPO 12 | -0.064 |
| CSI 12-CPO 13 | -0.067 |
| CSI 12-CPO 14 | -0.048 |
| CSI 12-CPO 15 | -0.075 |
| CSI 12-CPO 16 | -0.067 |
| CSI 12-CPO 17 | -0.062 |
| CSI 13-CSI 13 | 0.000 |
| CSI 13-CSI 14 | 0.072 |
| CSI 13-CSI 15 | -0.006 |
| CSI 13-CSI 16 | 0.015 |
| CSI 13-CSI 17 | 0.015 |
| CSI 13-CSI 18 | -0.020 |
| CSI 13-CSI 19 | 0.015 |
| CSI 13-CSI 20 | 0.030 |
| CSI 13-CSI 21 | -0.022 |
| CSI 13-CSI 22 | 0.017 |
| CSI 13-CSI 23 | -0.013 |
| CSI 13-CSI 24 | -0.001 |
| CSI 13-CSI 25 | -0.001 |
| CSI 13-CSI 26 | -0.009 |
| CSI 13-CSI 27 | -0.013 |
| CSI 13-CSI 28 | -0.017 |
| CSI 13-CSI 29 | 0.042 |
| CSI 13-CSI 30 | 0.042 |
| CSI 13-CSI 31 | 0.003 |
| CSI 13-CSI 32 | 0.016 |
| CSI 13-CSI 33 | -0.009 |
| CSI 13-CSI 34 | -0.019 |
| CSI 13-CSI 35 | 0.012 |
| CSI 13-CSI 36 | -0.003 |
| CSI 13-CSI 37 | -0.008 |
| CSI 13-CSI 38 | 0.003 |
| CSI 13-CSI 39 | -0.013 |
| CSI 13-CSI 40 | 0.012 |
| CSI 13-CSI 41 | -0.016 |
| CSI 13-CSI 42 | 0.010 |
| CSI 13-CSI 43 | -0.017 |
| CSI 13-CSI 44 | 0.005 |
| CSI 13-CSI 45 | -0.018 |
| CSI 13-CSI 46 | 0.000 |
| CSI 13-CSI 47 | 0.017 |
| CSI 13-CSI 48 | -0.007 |
| CSI 13-CSI 49 | 0.011 |
| CSI 13-CSI 50 | -0.016 |
| CSI 13-CSI 51 | 0.003 |
| CSI 13-CSI 52 | 0.002 |
| CSI 13-CPO 01 | -0.059 |
| CSI 13-CPO 02 | -0.059 |
| CSI 13-CPO 03 | -0.055 |
| CSI 13-CPO 04 | -0.057 |
| CSI 13-CPO 05 | -0.072 |
| CSI 13-CPO 06 | -0.076 |
| CSI 13-CPO 07 | -0.066 |
| CSI 13-CPO 08 | -0.066 |
| CSI 13-CPO 09 | -0.028 |
| CSI 13-CPO 10 | -0.072 |
| CSI 13-CPO 11 | -0.074 |
| CSI 13-CPO 12 | -0.069 |
| CSI 13-CPO 13 | -0.067 |
| CSI 13-CPO 14 | -0.066 |
| CSI 13-CPO 15 | -0.061 |
| CSI 13-CPO 16 | -0.059 |
| CSI 13-CPO 17 | -0.063 |
| CSI 14-CSI 14 | 0.000 |
| CSI 14-CSI 15 | 0.080 |
| CSI 14-CSI 16 | 0.117 |
| CSI 14-CSI 17 | 0.035 |
| CSI 14-CSI 18 | 0.018 |
| CSI 14-CSI 19 | 0.048 |
| CSI 14-CSI 20 | -0.024 |
| CSI 14-CSI 21 | -0.008 |
| CSI 14-CSI 22 | 0.023 |
| CSI 14-CSI 23 | -0.024 |
| CSI 14-CSI 24 | -0.028 |
| CSI 14-CSI 25 | -0.021 |
| CSI 14-CSI 26 | -0.034 |
| CSI 14-CSI 27 | -0.022 |
| CSI 14-CSI 28 | -0.013 |
| CSI 14-CSI 29 | 0.044 |
| CSI 14-CSI 30 | 0.023 |
| CSI 14-CSI 31 | -0.017 |
| CSI 14-CSI 32 | -0.027 |
| CSI 14-CSI 33 | 0.047 |
| CSI 14-CSI 34 | 0.054 |
| CSI 14-CSI 35 | -0.011 |
| CSI 14-CSI 36 | 0.051 |
| CSI 14-CSI 37 | -0.013 |
| CSI 14-CSI 38 | -0.021 |
| CSI 14-CSI 39 | -0.023 |
| CSI 14-CSI 40 | -0.031 |
| CSI 14-CSI 41 | -0.026 |
| CSI 14-CSI 42 | -0.017 |
| CSI 14-CSI 43 | -0.034 |
| CSI 14-CSI 44 | 0.003 |
| CSI 14-CSI 45 | -0.002 |
| CSI 14-CSI 46 | 0.005 |
| CSI 14-CSI 47 | -0.002 |
| CSI 14-CSI 48 | 0.087 |
| CSI 14-CSI 49 | 0.035 |
| CSI 14-CSI 50 | 0.015 |
| CSI 14-CSI 51 | -0.003 |
| CSI 14-CSI 52 | 0.005 |
| CSI 14-CPO 01 | -0.067 |
| CSI 14-CPO 02 | -0.067 |
| CSI 14-CPO 03 | -0.065 |
| CSI 14-CPO 04 | -0.065 |
| CSI 14-CPO 05 | -0.071 |
| CSI 14-CPO 06 | -0.071 |
| CSI 14-CPO 07 | -0.071 |
| CSI 14-CPO 08 | -0.061 |
| CSI 14-CPO 09 | -0.035 |
| CSI 14-CPO 10 | -0.079 |
| CSI 14-CPO 11 | -0.061 |
| CSI 14-CPO 12 | -0.069 |
| CSI 14-CPO 13 | -0.067 |
| CSI 14-CPO 14 | -0.053 |
| CSI 14-CPO 15 | -0.071 |
| CSI 14-CPO 16 | -0.069 |
| CSI 14-CPO 17 | -0.066 |
| CSI 15-CSI 15 | 0.000 |
| CSI 15-CSI 16 | 0.074 |
| CSI 15-CSI 17 | -0.004 |
| CSI 15-CSI 18 | 0.065 |
| CSI 15-CSI 19 | 0.036 |
| CSI 15-CSI 20 | -0.003 |
| CSI 15-CSI 21 | 0.046 |
| CSI 15-CSI 22 | -0.017 |
| CSI 15-CSI 23 | -0.021 |
| CSI 15-CSI 24 | -0.014 |
| CSI 15-CSI 25 | 0.060 |
| CSI 15-CSI 26 | -0.030 |
| CSI 15-CSI 27 | -0.010 |
| CSI 15-CSI 28 | -0.011 |
| CSI 15-CSI 29 | 0.043 |
| CSI 15-CSI 30 | 0.013 |
| CSI 15-CSI 31 | -0.017 |
| CSI 15-CSI 32 | -0.028 |
| CSI 15-CSI 33 | -0.007 |
| CSI 15-CSI 34 | -0.029 |
| CSI 15-CSI 35 | 0.046 |
| CSI 15-CSI 36 | 0.009 |
| CSI 15-CSI 37 | 0.007 |
| CSI 15-CSI 38 | 0.020 |
| CSI 15-CSI 39 | -0.003 |
| CSI 15-CSI 40 | 0.034 |
| CSI 15-CSI 41 | 0.030 |
| CSI 15-CSI 42 | 0.014 |
| CSI 15-CSI 43 | 0.021 |
| CSI 15-CSI 44 | -0.026 |
| CSI 15-CSI 45 | -0.014 |
| CSI 15-CSI 46 | -0.021 |
| CSI 15-CSI 47 | -0.014 |
| CSI 15-CSI 48 | -0.021 |
| CSI 15-CSI 49 | 0.037 |
| CSI 15-CSI 50 | 0.056 |
| CSI 15-CSI 51 | 0.020 |
| CSI 15-CSI 52 | -0.019 |
| CSI 15-CPO 01 | -0.050 |
| CSI 15-CPO 02 | -0.050 |
| CSI 15-CPO 03 | -0.037 |
| CSI 15-CPO 04 | -0.043 |
| CSI 15-CPO 05 | -0.051 |
| CSI 15-CPO 06 | -0.051 |
| CSI 15-CPO 07 | -0.066 |
| CSI 15-CPO 08 | -0.056 |
| CSI 15-CPO 09 | -0.035 |
| CSI 15-CPO 10 | -0.079 |
| CSI 15-CPO 11 | -0.064 |
| CSI 15-CPO 12 | -0.066 |
| CSI 15-CPO 13 | -0.067 |
| CSI 15-CPO 14 | -0.072 |
| CSI 15-CPO 15 | -0.075 |
| CSI 15-CPO 16 | -0.066 |
| CSI 15-CPO 17 | -0.051 |
| CSI 16-CSI 16 | 0.000 |
| CSI 16-CSI 17 | 0.022 |
| CSI 16-CSI 18 | 0.034 |
| CSI 16-CSI 19 | 0.029 |
| CSI 16-CSI 20 | -0.015 |
| CSI 16-CSI 21 | 0.000 |
| CSI 16-CSI 22 | 0.000 |
| CSI 16-CSI 23 | 0.022 |
| CSI 16-CSI 24 | 0.007 |
| CSI 16-CSI 25 | -0.018 |
| CSI 16-CSI 26 | -0.027 |
| CSI 16-CSI 27 | 0.014 |
| CSI 16-CSI 28 | 0.008 |
| CSI 16-CSI 29 | 0.017 |
| CSI 16-CSI 30 | 0.004 |
| CSI 16-CSI 31 | 0.038 |
| CSI 16-CSI 32 | 0.004 |
| CSI 16-CSI 33 | 0.007 |
| CSI 16-CSI 34 | 0.022 |
| CSI 16-CSI 35 | 0.016 |
| CSI 16-CSI 36 | 0.022 |
| CSI 16-CSI 37 | 0.024 |
| CSI 16-CSI 38 | 0.010 |
| CSI 16-CSI 39 | 0.009 |
| CSI 16-CSI 40 | 0.009 |
| CSI 16-CSI 41 | -0.015 |
| CSI 16-CSI 42 | -0.017 |
| CSI 16-CSI 43 | 0.017 |
| CSI 16-CSI 44 | -0.006 |
| CSI 16-CSI 45 | 0.013 |
| CSI 16-CSI 46 | -0.007 |
| CSI 16-CSI 47 | 0.021 |
| CSI 16-CSI 48 | 0.016 |
| CSI 16-CSI 49 | 0.020 |
| CSI 16-CSI 50 | 0.049 |
| CSI 16-CSI 51 | 0.009 |
| CSI 16-CSI 52 | 0.040 |
| CSI 16-CPO 01 | -0.050 |
| CSI 16-CPO 02 | -0.050 |
| CSI 16-CPO 03 | -0.041 |
| CSI 16-CPO 04 | -0.047 |
| CSI 16-CPO 05 | -0.054 |
| CSI 16-CPO 06 | -0.058 |
| CSI 16-CPO 07 | -0.065 |
| CSI 16-CPO 08 | -0.054 |
| CSI 16-CPO 09 | -0.012 |
| CSI 16-CPO 10 | -0.075 |
| CSI 16-CPO 11 | -0.035 |
| CSI 16-CPO 12 | -0.051 |
| CSI 16-CPO 13 | -0.058 |
| CSI 16-CPO 14 | -0.057 |
| CSI 16-CPO 15 | -0.068 |
| CSI 16-CPO 16 | -0.060 |
| CSI 16-CPO 17 | -0.040 |
| CSI 17-CSI 17 | 0.000 |
| CSI 17-CSI 18 | 0.029 |
| CSI 17-CSI 19 | 0.012 |
| CSI 17-CSI 20 | 0.055 |
| CSI 17-CSI 21 | -0.006 |
| CSI 17-CSI 22 | -0.012 |
| CSI 17-CSI 23 | -0.025 |
| CSI 17-CSI 24 | -0.019 |
| CSI 17-CSI 25 | -0.029 |
| CSI 17-CSI 26 | -0.030 |
| CSI 17-CSI 27 | -0.004 |
| CSI 17-CSI 28 | -0.017 |
| CSI 17-CSI 29 | 0.018 |
| CSI 17-CSI 30 | 0.018 |
| CSI 17-CSI 31 | -0.025 |
| CSI 17-CSI 32 | -0.015 |
| CSI 17-CSI 33 | -0.015 |
| CSI 17-CSI 34 | 0.009 |
| CSI 17-CSI 35 | -0.010 |
| CSI 17-CSI 36 | 0.004 |
| CSI 17-CSI 37 | -0.018 |
| CSI 17-CSI 38 | -0.001 |
| CSI 17-CSI 39 | 0.007 |
| CSI 17-CSI 40 | -0.018 |
| CSI 17-CSI 41 | -0.008 |
| CSI 17-CSI 42 | -0.035 |
| CSI 17-CSI 43 | -0.021 |
| CSI 17-CSI 44 | -0.014 |
| CSI 17-CSI 45 | -0.031 |
| CSI 17-CSI 46 | -0.022 |
| CSI 17-CSI 47 | 0.013 |
| CSI 17-CSI 48 | 0.024 |
| CSI 17-CSI 49 | -0.001 |
| CSI 17-CSI 50 | -0.005 |
| CSI 17-CSI 51 | 0.005 |
| CSI 17-CSI 52 | 0.027 |
| CSI 17-CPO 01 | -0.041 |
| CSI 17-CPO 02 | -0.041 |
| CSI 17-CPO 03 | -0.033 |
| CSI 17-CPO 04 | -0.039 |
| CSI 17-CPO 05 | -0.051 |
| CSI 17-CPO 06 | -0.051 |
| CSI 17-CPO 07 | -0.057 |
| CSI 17-CPO 08 | -0.057 |
| CSI 17-CPO 09 | -0.021 |
| CSI 17-CPO 10 | -0.075 |
| CSI 17-CPO 11 | -0.075 |
| CSI 17-CPO 12 | -0.066 |
| CSI 17-CPO 13 | -0.073 |
| CSI 17-CPO 14 | -0.031 |
| CSI 17-CPO 15 | -0.075 |
| CSI 17-CPO 16 | -0.064 |
| CSI 17-CPO 17 | -0.028 |
| CSI 18-CSI 18 | 0.000 |
| CSI 18-CSI 19 | 0.154 |
| CSI 18-CSI 20 | 0.157 |
| CSI 18-CSI 21 | 0.011 |
| CSI 18-CSI 22 | 0.030 |
| CSI 18-CSI 23 | 0.055 |
| CSI 18-CSI 24 | 0.045 |
| CSI 18-CSI 25 | -0.026 |
| CSI 18-CSI 26 | -0.011 |
| CSI 18-CSI 27 | -0.017 |
| CSI 18-CSI 28 | -0.014 |
| CSI 18-CSI 29 | -0.004 |
| CSI 18-CSI 30 | 0.007 |
| CSI 18-CSI 31 | -0.031 |
| CSI 18-CSI 32 | -0.023 |
| CSI 18-CSI 33 | -0.033 |
| CSI 18-CSI 34 | -0.038 |
| CSI 18-CSI 35 | -0.024 |
| CSI 18-CSI 36 | -0.021 |
| CSI 18-CSI 37 | -0.022 |
| CSI 18-CSI 38 | -0.012 |
| CSI 18-CSI 39 | -0.022 |
| CSI 18-CSI 40 | -0.022 |
| CSI 18-CSI 41 | -0.026 |
| CSI 18-CSI 42 | 0.016 |
| CSI 18-CSI 43 | -0.027 |
| CSI 18-CSI 44 | -0.021 |
| CSI 18-CSI 45 | -0.020 |
| CSI 18-CSI 46 | -0.023 |
| CSI 18-CSI 47 | -0.020 |
| CSI 18-CSI 48 | 0.026 |
| CSI 18-CSI 49 | -0.016 |
| CSI 18-CSI 50 | 0.006 |
| CSI 18-CSI 51 | 0.015 |
| CSI 18-CSI 52 | 0.025 |
| CSI 18-CPO 01 | -0.036 |
| CSI 18-CPO 02 | -0.036 |
| CSI 18-CPO 03 | -0.044 |
| CSI 18-CPO 04 | -0.051 |
| CSI 18-CPO 05 | -0.064 |
| CSI 18-CPO 06 | -0.064 |
| CSI 18-CPO 07 | -0.032 |
| CSI 18-CPO 08 | -0.032 |
| CSI 18-CPO 09 | 0.068 |
| CSI 18-CPO 10 | -0.053 |
| CSI 18-CPO 11 | -0.070 |
| CSI 18-CPO 12 | -0.067 |
| CSI 18-CPO 13 | -0.040 |
| CSI 18-CPO 14 | -0.060 |
| CSI 18-CPO 15 | -0.037 |
| CSI 18-CPO 16 | -0.046 |
| CSI 18-CPO 17 | -0.048 |
| CSI 19-CSI 19 | 0.000 |
| CSI 19-CSI 20 | 0.056 |
| CSI 19-CSI 21 | -0.012 |
| CSI 19-CSI 22 | 0.039 |
| CSI 19-CSI 23 | 0.064 |
| CSI 19-CSI 24 | -0.019 |
| CSI 19-CSI 25 | -0.027 |
| CSI 19-CSI 26 | -0.024 |
| CSI 19-CSI 27 | -0.011 |
| CSI 19-CSI 28 | -0.001 |
| CSI 19-CSI 29 | -0.006 |
| CSI 19-CSI 30 | -0.016 |
| CSI 19-CSI 31 | -0.016 |
| CSI 19-CSI 32 | -0.003 |
| CSI 19-CSI 33 | -0.021 |
| CSI 19-CSI 34 | -0.018 |
| CSI 19-CSI 35 | -0.023 |
| CSI 19-CSI 36 | -0.023 |
| CSI 19-CSI 37 | -0.007 |
| CSI 19-CSI 38 | -0.006 |
| CSI 19-CSI 39 | -0.011 |
| CSI 19-CSI 40 | -0.003 |
| CSI 19-CSI 41 | -0.011 |
| CSI 19-CSI 42 | 0.042 |
| CSI 19-CSI 43 | -0.005 |
| CSI 19-CSI 44 | 0.009 |
| CSI 19-CSI 45 | 0.002 |
| CSI 19-CSI 46 | 0.017 |
| CSI 19-CSI 47 | 0.024 |
| CSI 19-CSI 48 | 0.045 |
| CSI 19-CSI 49 | 0.016 |
| CSI 19-CSI 50 | 0.016 |
| CSI 19-CSI 51 | 0.009 |
| CSI 19-CSI 52 | 0.012 |
| CSI 19-CPO 01 | -0.063 |
| CSI 19-CPO 02 | -0.063 |
| CSI 19-CPO 03 | -0.051 |
| CSI 19-CPO 04 | -0.055 |
| CSI 19-CPO 05 | -0.056 |
| CSI 19-CPO 06 | -0.060 |
| CSI 19-CPO 07 | -0.069 |
| CSI 19-CPO 08 | -0.069 |
| CSI 19-CPO 09 | -0.023 |
| CSI 19-CPO 10 | -0.079 |
| CSI 19-CPO 11 | -0.077 |
| CSI 19-CPO 12 | -0.071 |
| CSI 19-CPO 13 | -0.072 |
| CSI 19-CPO 14 | -0.067 |
| CSI 19-CPO 15 | -0.072 |
| CSI 19-CPO 16 | -0.064 |
| CSI 19-CPO 17 | -0.056 |
| CSI 20-CSI 20 | 0.000 |
| CSI 20-CSI 21 | 0.057 |
| CSI 20-CSI 22 | 0.008 |
| CSI 20-CSI 23 | 0.007 |
| CSI 20-CSI 24 | 0.006 |
| CSI 20-CSI 25 | 0.035 |
| CSI 20-CSI 26 | 0.013 |
| CSI 20-CSI 27 | -0.001 |
| CSI 20-CSI 28 | -0.008 |
| CSI 20-CSI 29 | 0.038 |
| CSI 20-CSI 30 | 0.038 |
| CSI 20-CSI 31 | 0.004 |
| CSI 20-CSI 32 | 0.022 |
| CSI 20-CSI 33 | -0.019 |
| CSI 20-CSI 34 | 0.003 |
| CSI 20-CSI 35 | 0.000 |
| CSI 20-CSI 36 | 0.003 |
| CSI 20-CSI 37 | -0.017 |
| CSI 20-CSI 38 | -0.017 |
| CSI 20-CSI 39 | -0.007 |
| CSI 20-CSI 40 | -0.021 |
| CSI 20-CSI 41 | -0.018 |
| CSI 20-CSI 42 | -0.031 |
| CSI 20-CSI 43 | -0.027 |
| CSI 20-CSI 44 | 0.012 |
| CSI 20-CSI 45 | 0.031 |
| CSI 20-CSI 46 | -0.014 |
| CSI 20-CSI 47 | 0.000 |
| CSI 20-CSI 48 | 0.039 |
| CSI 20-CSI 49 | -0.022 |
| CSI 20-CSI 50 | -0.015 |
| CSI 20-CSI 51 | 0.030 |
| CSI 20-CSI 52 | 0.049 |
| CSI 20-CPO 01 | -0.070 |
| CSI 20-CPO 02 | -0.070 |
| CSI 20-CPO 03 | -0.068 |
| CSI 20-CPO 04 | -0.070 |
| CSI 20-CPO 05 | -0.076 |
| CSI 20-CPO 06 | -0.076 |
| CSI 20-CPO 07 | -0.074 |
| CSI 20-CPO 08 | -0.074 |
| CSI 20-CPO 09 | -0.041 |
| CSI 20-CPO 10 | -0.071 |
| CSI 20-CPO 11 | -0.074 |
| CSI 20-CPO 12 | -0.069 |
| CSI 20-CPO 13 | -0.059 |
| CSI 20-CPO 14 | -0.047 |
| CSI 20-CPO 15 | -0.074 |
| CSI 20-CPO 16 | -0.051 |
| CSI 20-CPO 17 | 0.022 |
| CSI 21-CSI 21 | 0.000 |
| CSI 21-CSI 22 | -0.018 |
| CSI 21-CSI 23 | 0.005 |
| CSI 21-CSI 24 | -0.002 |
| CSI 21-CSI 25 | -0.016 |
| CSI 21-CSI 26 | 0.002 |
| CSI 21-CSI 27 | -0.002 |
| CSI 21-CSI 28 | -0.007 |
| CSI 21-CSI 29 | -0.022 |
| CSI 21-CSI 30 | -0.022 |
| CSI 21-CSI 31 | -0.002 |
| CSI 21-CSI 32 | -0.002 |
| CSI 21-CSI 33 | -0.009 |
| CSI 21-CSI 34 | -0.002 |
| CSI 21-CSI 35 | 0.019 |
| CSI 21-CSI 36 | 0.037 |
| CSI 21-CSI 37 | 0.005 |
| CSI 21-CSI 38 | 0.007 |
| CSI 21-CSI 39 | 0.005 |
| CSI 21-CSI 40 | -0.002 |
| CSI 21-CSI 41 | 0.010 |
| CSI 21-CSI 42 | 0.078 |
| CSI 21-CSI 43 | 0.039 |
| CSI 21-CSI 44 | 0.043 |
| CSI 21-CSI 45 | 0.042 |
| CSI 21-CSI 46 | 0.018 |
| CSI 21-CSI 47 | -0.006 |
| CSI 21-CSI 48 | -0.005 |
| CSI 21-CSI 49 | 0.013 |
| CSI 21-CSI 50 | 0.016 |
| CSI 21-CSI 51 | 0.020 |
| CSI 21-CSI 52 | 0.027 |
| CSI 21-CPO 01 | -0.034 |
| CSI 21-CPO 02 | -0.034 |
| CSI 21-CPO 03 | -0.071 |
| CSI 21-CPO 04 | -0.062 |
| CSI 21-CPO 05 | -0.033 |
| CSI 21-CPO 06 | -0.037 |
| CSI 21-CPO 07 | -0.010 |
| CSI 21-CPO 08 | -0.010 |
| CSI 21-CPO 09 | -0.019 |
| CSI 21-CPO 10 | -0.054 |
| CSI 21-CPO 11 | -0.069 |
| CSI 21-CPO 12 | -0.056 |
| CSI 21-CPO 13 | -0.045 |
| CSI 21-CPO 14 | -0.051 |
| CSI 21-CPO 15 | -0.069 |
| CSI 21-CPO 16 | -0.069 |
| CSI 21-CPO 17 | -0.067 |
| CSI 22-CSI 22 | 0.000 |
| CSI 22-CSI 23 | 0.058 |
| CSI 22-CSI 24 | 0.221 |
| CSI 22-CSI 25 | 0.032 |
| CSI 22-CSI 26 | -0.017 |
| CSI 22-CSI 27 | 0.056 |
| CSI 22-CSI 28 | 0.025 |
| CSI 22-CSI 29 | -0.015 |
| CSI 22-CSI 30 | -0.030 |
| CSI 22-CSI 31 | -0.024 |
| CSI 22-CSI 32 | -0.019 |
| CSI 22-CSI 33 | 0.034 |
| CSI 22-CSI 34 | -0.002 |
| CSI 22-CSI 35 | -0.013 |
| CSI 22-CSI 36 | 0.045 |
| CSI 22-CSI 37 | 0.011 |
| CSI 22-CSI 38 | -0.007 |
| CSI 22-CSI 39 | 0.006 |
| CSI 22-CSI 40 | 0.006 |
| CSI 22-CSI 41 | -0.001 |
| CSI 22-CSI 42 | -0.014 |
| CSI 22-CSI 43 | 0.006 |
| CSI 22-CSI 44 | -0.014 |
| CSI 22-CSI 45 | -0.016 |
| CSI 22-CSI 46 | -0.005 |
| CSI 22-CSI 47 | -0.009 |
| CSI 22-CSI 48 | 0.021 |
| CSI 22-CSI 49 | 0.019 |
| CSI 22-CSI 50 | 0.024 |
| CSI 22-CSI 51 | 0.014 |
| CSI 22-CSI 52 | 0.034 |
| CSI 22-CPO 01 | -0.055 |
| CSI 22-CPO 02 | -0.055 |
| CSI 22-CPO 03 | -0.050 |
| CSI 22-CPO 04 | -0.054 |
| CSI 22-CPO 05 | -0.072 |
| CSI 22-CPO 06 | -0.072 |
| CSI 22-CPO 07 | -0.073 |
| CSI 22-CPO 08 | -0.073 |
| CSI 22-CPO 09 | -0.015 |
| CSI 22-CPO 10 | -0.072 |
| CSI 22-CPO 11 | -0.077 |
| CSI 22-CPO 12 | -0.065 |
| CSI 22-CPO 13 | -0.052 |
| CSI 22-CPO 14 | -0.062 |
| CSI 22-CPO 15 | -0.040 |
| CSI 22-CPO 16 | -0.062 |
| CSI 22-CPO 17 | -0.059 |
| CSI 23-CSI 23 | 0.000 |
| CSI 23-CSI 24 | 0.101 |
| CSI 23-CSI 25 | -0.001 |
| CSI 23-CSI 26 | -0.013 |
| CSI 23-CSI 27 | -0.004 |
| CSI 23-CSI 28 | 0.015 |
| CSI 23-CSI 29 | -0.034 |
| CSI 23-CSI 30 | -0.030 |
| CSI 23-CSI 31 | 0.009 |
| CSI 23-CSI 32 | 0.009 |
| CSI 23-CSI 33 | 0.033 |
| CSI 23-CSI 34 | -0.005 |
| CSI 23-CSI 35 | 0.004 |
| CSI 23-CSI 36 | 0.024 |
| CSI 23-CSI 37 | 0.016 |
| CSI 23-CSI 38 | 0.017 |
| CSI 23-CSI 39 | 0.033 |
| CSI 23-CSI 40 | 0.016 |
| CSI 23-CSI 41 | 0.011 |
| CSI 23-CSI 42 | 0.003 |
| CSI 23-CSI 43 | 0.002 |
| CSI 23-CSI 44 | -0.008 |
| CSI 23-CSI 45 | 0.060 |
| CSI 23-CSI 46 | 0.042 |
| CSI 23-CSI 47 | 0.002 |
| CSI 23-CSI 48 | 0.006 |
| CSI 23-CSI 49 | 0.001 |
| CSI 23-CSI 50 | 0.043 |
| CSI 23-CSI 51 | 0.000 |
| CSI 23-CSI 52 | 0.017 |
| CSI 23-CPO 01 | -0.043 |
| CSI 23-CPO 02 | -0.043 |
| CSI 23-CPO 03 | -0.067 |
| CSI 23-CPO 04 | -0.050 |
| CSI 23-CPO 05 | -0.079 |
| CSI 23-CPO 06 | -0.079 |
| CSI 23-CPO 07 | -0.073 |
| CSI 23-CPO 08 | -0.073 |
| CSI 23-CPO 09 | -0.038 |
| CSI 23-CPO 10 | -0.061 |
| CSI 23-CPO 11 | -0.073 |
| CSI 23-CPO 12 | -0.077 |
| CSI 23-CPO 13 | -0.055 |
| CSI 23-CPO 14 | -0.052 |
| CSI 23-CPO 15 | -0.073 |
| CSI 23-CPO 16 | -0.077 |
| CSI 23-CPO 17 | -0.052 |
| CSI 24-CSI 24 | 0.000 |
| CSI 24-CSI 25 | -0.002 |
| CSI 24-CSI 26 | 0.015 |
| CSI 24-CSI 27 | 0.018 |
| CSI 24-CSI 28 | 0.025 |
| CSI 24-CSI 29 | -0.013 |
| CSI 24-CSI 30 | -0.023 |
| CSI 24-CSI 31 | -0.017 |
| CSI 24-CSI 32 | -0.005 |
| CSI 24-CSI 33 | -0.002 |
| CSI 24-CSI 34 | 0.001 |
| CSI 24-CSI 35 | -0.011 |
| CSI 24-CSI 36 | 0.006 |
| CSI 24-CSI 37 | 0.022 |
| CSI 24-CSI 38 | 0.022 |
| CSI 24-CSI 39 | 0.018 |
| CSI 24-CSI 40 | 0.018 |
| CSI 24-CSI 41 | 0.012 |
| CSI 24-CSI 42 | 0.005 |
| CSI 24-CSI 43 | 0.030 |
| CSI 24-CSI 44 | 0.007 |
| CSI 24-CSI 45 | 0.003 |
| CSI 24-CSI 46 | 0.024 |
| CSI 24-CSI 47 | 0.020 |
| CSI 24-CSI 48 | 0.037 |
| CSI 24-CSI 49 | 0.021 |
| CSI 24-CSI 50 | 0.033 |
| CSI 24-CSI 51 | 0.048 |
| CSI 24-CSI 52 | 0.062 |
| CSI 24-CPO 01 | -0.060 |
| CSI 24-CPO 02 | -0.060 |
| CSI 24-CPO 03 | -0.047 |
| CSI 24-CPO 04 | -0.049 |
| CSI 24-CPO 05 | -0.067 |
| CSI 24-CPO 06 | -0.067 |
| CSI 24-CPO 07 | -0.040 |
| CSI 24-CPO 08 | -0.040 |
| CSI 24-CPO 09 | -0.046 |
| CSI 24-CPO 10 | -0.072 |
| CSI 24-CPO 11 | -0.075 |
| CSI 24-CPO 12 | -0.065 |
| CSI 24-CPO 13 | -0.050 |
| CSI 24-CPO 14 | -0.055 |
| CSI 24-CPO 15 | -0.037 |
| CSI 24-CPO 16 | -0.060 |
| CSI 24-CPO 17 | -0.059 |
| CSI 25-CSI 25 | 0.000 |
| CSI 25-CSI 26 | 0.012 |
| CSI 25-CSI 27 | 0.072 |
| CSI 25-CSI 28 | 0.017 |
| CSI 25-CSI 29 | -0.003 |
| CSI 25-CSI 30 | 0.018 |
| CSI 25-CSI 31 | -0.017 |
| CSI 25-CSI 32 | -0.017 |
| CSI 25-CSI 33 | 0.027 |
| CSI 25-CSI 34 | 0.050 |
| CSI 25-CSI 35 | 0.000 |
| CSI 25-CSI 36 | 0.128 |
| CSI 25-CSI 37 | -0.014 |
| CSI 25-CSI 38 | 0.002 |
| CSI 25-CSI 39 | -0.014 |
| CSI 25-CSI 40 | 0.017 |
| CSI 25-CSI 41 | 0.095 |
| CSI 25-CSI 42 | 0.011 |
| CSI 25-CSI 43 | 0.016 |
| CSI 25-CSI 44 | -0.001 |
| CSI 25-CSI 45 | -0.011 |
| CSI 25-CSI 46 | -0.007 |
| CSI 25-CSI 47 | 0.002 |
| CSI 25-CSI 48 | -0.027 |
| CSI 25-CSI 49 | -0.006 |
| CSI 25-CSI 50 | -0.006 |
| CSI 25-CSI 51 | -0.007 |
| CSI 25-CSI 52 | 0.003 |
| CSI 25-CPO 01 | -0.064 |
| CSI 25-CPO 02 | -0.064 |
| CSI 25-CPO 03 | -0.041 |
| CSI 25-CPO 04 | -0.044 |
| CSI 25-CPO 05 | -0.047 |
| CSI 25-CPO 06 | -0.047 |
| CSI 25-CPO 07 | -0.068 |
| CSI 25-CPO 08 | -0.068 |
| CSI 25-CPO 09 | -0.018 |
| CSI 25-CPO 10 | -0.076 |
| CSI 25-CPO 11 | -0.050 |
| CSI 25-CPO 12 | -0.063 |
| CSI 25-CPO 13 | -0.077 |
| CSI 25-CPO 14 | -0.066 |
| CSI 25-CPO 15 | -0.068 |
| CSI 25-CPO 16 | -0.063 |
| CSI 25-CPO 17 | -0.057 |
| CSI 26-CSI 26 | 0.000 |
| CSI 26-CSI 27 | 0.037 |
| CSI 26-CSI 28 | -0.004 |
| CSI 26-CSI 29 | -0.009 |
| CSI 26-CSI 30 | -0.016 |
| CSI 26-CSI 31 | 0.004 |
| CSI 26-CSI 32 | 0.004 |
| CSI 26-CSI 33 | -0.016 |
| CSI 26-CSI 34 | 0.011 |
| CSI 26-CSI 35 | -0.013 |
| CSI 26-CSI 36 | -0.025 |
| CSI 26-CSI 37 | 0.009 |
| CSI 26-CSI 38 | 0.010 |
| CSI 26-CSI 39 | 0.009 |
| CSI 26-CSI 40 | 0.009 |
| CSI 26-CSI 41 | 0.045 |
| CSI 26-CSI 42 | -0.002 |
| CSI 26-CSI 43 | 0.008 |
| CSI 26-CSI 44 | 0.007 |
| CSI 26-CSI 45 | -0.019 |
| CSI 26-CSI 46 | 0.003 |
| CSI 26-CSI 47 | -0.002 |
| CSI 26-CSI 48 | -0.026 |
| CSI 26-CSI 49 | 0.003 |
| CSI 26-CSI 50 | -0.010 |
| CSI 26-CSI 51 | 0.020 |
| CSI 26-CSI 52 | 0.021 |
| CSI 26-CPO 01 | -0.033 |
| CSI 26-CPO 02 | -0.033 |
| CSI 26-CPO 03 | -0.035 |
| CSI 26-CPO 04 | -0.023 |
| CSI 26-CPO 05 | -0.047 |
| CSI 26-CPO 06 | -0.051 |
| CSI 26-CPO 07 | -0.048 |
| CSI 26-CPO 08 | -0.048 |
| CSI 26-CPO 09 | -0.052 |
| CSI 26-CPO 10 | -0.044 |
| CSI 26-CPO 11 | -0.055 |
| CSI 26-CPO 12 | 0.024 |
| CSI 26-CPO 13 | -0.048 |
| CSI 26-CPO 14 | -0.029 |
| CSI 26-CPO 15 | -0.034 |
| CSI 26-CPO 16 | -0.024 |
| CSI 26-CPO 17 | -0.017 |
| CSI 27-CSI 27 | 0.000 |
| CSI 27-CSI 28 | 0.188 |
| CSI 27-CSI 29 | 0.009 |
| CSI 27-CSI 30 | -0.005 |
| CSI 27-CSI 31 | 0.014 |
| CSI 27-CSI 32 | 0.056 |
| CSI 27-CSI 33 | 0.011 |
| CSI 27-CSI 34 | 0.014 |
| CSI 27-CSI 35 | -0.006 |
| CSI 27-CSI 36 | -0.003 |
| CSI 27-CSI 37 | 0.004 |
| CSI 27-CSI 38 | 0.011 |
| CSI 27-CSI 39 | 0.004 |
| CSI 27-CSI 40 | 0.004 |
| CSI 27-CSI 41 | 0.039 |
| CSI 27-CSI 42 | 0.011 |
| CSI 27-CSI 43 | 0.034 |
| CSI 27-CSI 44 | 0.019 |
| CSI 27-CSI 45 | 0.008 |
| CSI 27-CSI 46 | 0.011 |
| CSI 27-CSI 47 | 0.008 |
| CSI 27-CSI 48 | -0.008 |
| CSI 27-CSI 49 | 0.010 |
| CSI 27-CSI 50 | 0.024 |
| CSI 27-CSI 51 | 0.016 |
| CSI 27-CSI 52 | 0.025 |
| CSI 27-CPO 01 | -0.071 |
| CSI 27-CPO 02 | -0.071 |
| CSI 27-CPO 03 | -0.053 |
| CSI 27-CPO 04 | -0.069 |
| CSI 27-CPO 05 | -0.042 |
| CSI 27-CPO 06 | -0.046 |
| CSI 27-CPO 07 | -0.062 |
| CSI 27-CPO 08 | -0.062 |
| CSI 27-CPO 09 | -0.062 |
| CSI 27-CPO 10 | -0.068 |
| CSI 27-CPO 11 | -0.073 |
| CSI 27-CPO 12 | -0.055 |
| CSI 27-CPO 13 | -0.066 |
| CSI 27-CPO 14 | -0.051 |
| CSI 27-CPO 15 | -0.058 |
| CSI 27-CPO 16 | -0.039 |
| CSI 27-CPO 17 | -0.033 |
| CSI 28-CSI 28 | 0.000 |
| CSI 28-CSI 29 | 0.026 |
| CSI 28-CSI 30 | 0.022 |
| CSI 28-CSI 31 | 0.030 |
| CSI 28-CSI 32 | 0.035 |
| CSI 28-CSI 33 | 0.008 |
| CSI 28-CSI 34 | 0.006 |
| CSI 28-CSI 35 | 0.020 |
| CSI 28-CSI 36 | 0.032 |
| CSI 28-CSI 37 | 0.019 |
| CSI 28-CSI 38 | 0.012 |
| CSI 28-CSI 39 | 0.014 |
| CSI 28-CSI 40 | 0.014 |
| CSI 28-CSI 41 | 0.018 |
| CSI 28-CSI 42 | 0.018 |
| CSI 28-CSI 43 | 0.028 |
| CSI 28-CSI 44 | 0.014 |
| CSI 28-CSI 45 | -0.003 |
| CSI 28-CSI 46 | 0.008 |
| CSI 28-CSI 47 | 0.001 |
| CSI 28-CSI 48 | 0.004 |
| CSI 28-CSI 49 | 0.002 |
| CSI 28-CSI 50 | 0.022 |
| CSI 28-CSI 51 | 0.003 |
| CSI 28-CSI 52 | 0.006 |
| CSI 28-CPO 01 | -0.063 |
| CSI 28-CPO 02 | -0.063 |
| CSI 28-CPO 03 | -0.047 |
| CSI 28-CPO 04 | -0.060 |
| CSI 28-CPO 05 | -0.031 |
| CSI 28-CPO 06 | -0.035 |
| CSI 28-CPO 07 | -0.058 |
| CSI 28-CPO 08 | -0.058 |
| CSI 28-CPO 09 | -0.056 |
| CSI 28-CPO 10 | -0.054 |
| CSI 28-CPO 11 | -0.073 |
| CSI 28-CPO 12 | -0.057 |
| CSI 28-CPO 13 | -0.034 |
| CSI 28-CPO 14 | -0.052 |
| CSI 28-CPO 15 | -0.054 |
| CSI 28-CPO 16 | -0.042 |
| CSI 28-CPO 17 | -0.036 |
| CSI 29-CSI 29 | 0.000 |
| CSI 29-CSI 30 | 0.221 |
| CSI 29-CSI 31 | 0.080 |
| CSI 29-CSI 32 | 0.015 |
| CSI 29-CSI 33 | 0.005 |
| CSI 29-CSI 34 | -0.004 |
| CSI 29-CSI 35 | 0.041 |
| CSI 29-CSI 36 | -0.013 |
| CSI 29-CSI 37 | -0.005 |
| CSI 29-CSI 38 | 0.004 |
| CSI 29-CSI 39 | -0.010 |
| CSI 29-CSI 40 | 0.015 |
| CSI 29-CSI 41 | 0.026 |
| CSI 29-CSI 42 | 0.015 |
| CSI 29-CSI 43 | 0.004 |
| CSI 29-CSI 44 | 0.008 |
| CSI 29-CSI 45 | 0.000 |
| CSI 29-CSI 46 | -0.013 |
| CSI 29-CSI 47 | -0.014 |
| CSI 29-CSI 48 | -0.016 |
| CSI 29-CSI 49 | -0.005 |
| CSI 29-CSI 50 | -0.014 |
| CSI 29-CSI 51 | -0.031 |
| CSI 29-CSI 52 | 0.023 |
| CSI 29-CPO 01 | -0.063 |
| CSI 29-CPO 02 | -0.063 |
| CSI 29-CPO 03 | -0.053 |
| CSI 29-CPO 04 | -0.060 |
| CSI 29-CPO 05 | -0.048 |
| CSI 29-CPO 06 | -0.048 |
| CSI 29-CPO 07 | -0.053 |
| CSI 29-CPO 08 | -0.053 |
| CSI 29-CPO 09 | -0.056 |
| CSI 29-CPO 10 | -0.033 |
| CSI 29-CPO 11 | -0.044 |
| CSI 29-CPO 12 | -0.048 |
| CSI 29-CPO 13 | -0.026 |
| CSI 29-CPO 14 | -0.062 |
| CSI 29-CPO 15 | -0.052 |
| CSI 29-CPO 16 | -0.052 |
| CSI 29-CPO 17 | -0.028 |
| CSI 30-CSI 30 | 0.000 |
| CSI 30-CSI 31 | 0.099 |
| CSI 30-CSI 32 | 0.022 |
| CSI 30-CSI 33 | -0.004 |
| CSI 30-CSI 34 | 0.000 |
| CSI 30-CSI 35 | 0.029 |
| CSI 30-CSI 36 | -0.022 |
| CSI 30-CSI 37 | 0.007 |
| CSI 30-CSI 38 | 0.016 |
| CSI 30-CSI 39 | -0.003 |
| CSI 30-CSI 40 | 0.053 |
| CSI 30-CSI 41 | 0.004 |
| CSI 30-CSI 42 | 0.015 |
| CSI 30-CSI 43 | -0.005 |
| CSI 30-CSI 44 | 0.002 |
| CSI 30-CSI 45 | -0.001 |
| CSI 30-CSI 46 | -0.014 |
| CSI 30-CSI 47 | -0.015 |
| CSI 30-CSI 48 | -0.016 |
| CSI 30-CSI 49 | -0.004 |
| CSI 30-CSI 50 | -0.008 |
| CSI 30-CSI 51 | -0.023 |
| CSI 30-CSI 52 | 0.009 |
| CSI 30-CPO 01 | -0.064 |
| CSI 30-CPO 02 | -0.064 |
| CSI 30-CPO 03 | -0.050 |
| CSI 30-CPO 04 | -0.057 |
| CSI 30-CPO 05 | -0.057 |
| CSI 30-CPO 06 | -0.057 |
| CSI 30-CPO 07 | -0.053 |
| CSI 30-CPO 08 | -0.043 |
| CSI 30-CPO 09 | -0.052 |
| CSI 30-CPO 10 | -0.018 |
| CSI 30-CPO 11 | -0.061 |
| CSI 30-CPO 12 | -0.062 |
| CSI 30-CPO 13 | -0.005 |
| CSI 30-CPO 14 | -0.037 |
| CSI 30-CPO 15 | -0.049 |
| CSI 30-CPO 16 | -0.044 |
| CSI 30-CPO 17 | -0.038 |
| CSI 31-CSI 31 | 0.000 |
| CSI 31-CSI 32 | 0.204 |
| CSI 31-CSI 33 | 0.032 |
| CSI 31-CSI 34 | 0.012 |
| CSI 31-CSI 35 | 0.036 |
| CSI 31-CSI 36 | 0.006 |
| CSI 31-CSI 37 | 0.023 |
| CSI 31-CSI 38 | 0.007 |
| CSI 31-CSI 39 | 0.029 |
| CSI 31-CSI 40 | 0.013 |
| CSI 31-CSI 41 | 0.019 |
| CSI 31-CSI 42 | 0.015 |
| CSI 31-CSI 43 | 0.004 |
| CSI 31-CSI 44 | -0.006 |
| CSI 31-CSI 45 | -0.001 |
| CSI 31-CSI 46 | -0.010 |
| CSI 31-CSI 47 | -0.012 |
| CSI 31-CSI 48 | -0.003 |
| CSI 31-CSI 49 | 0.000 |
| CSI 31-CSI 50 | 0.006 |
| CSI 31-CSI 51 | -0.013 |
| CSI 31-CSI 52 | -0.007 |
| CSI 31-CPO 01 | -0.054 |
| CSI 31-CPO 02 | -0.054 |
| CSI 31-CPO 03 | -0.064 |
| CSI 31-CPO 04 | -0.052 |
| CSI 31-CPO 05 | -0.069 |
| CSI 31-CPO 06 | -0.073 |
| CSI 31-CPO 07 | -0.063 |
| CSI 31-CPO 08 | -0.052 |
| CSI 31-CPO 09 | -0.056 |
| CSI 31-CPO 10 | -0.049 |
| CSI 31-CPO 11 | -0.001 |
| CSI 31-CPO 12 | -0.043 |
| CSI 31-CPO 13 | -0.043 |
| CSI 31-CPO 14 | -0.018 |
| CSI 31-CPO 15 | -0.067 |
| CSI 31-CPO 16 | -0.067 |
| CSI 31-CPO 17 | -0.003 |
| CSI 32-CSI 32 | 0.000 |
| CSI 32-CSI 33 | 0.037 |
| CSI 32-CSI 34 | 0.022 |
| CSI 32-CSI 35 | 0.023 |
| CSI 32-CSI 36 | 0.020 |
| CSI 32-CSI 37 | 0.018 |
| CSI 32-CSI 38 | 0.016 |
| CSI 32-CSI 39 | 0.039 |
| CSI 32-CSI 40 | 0.013 |
| CSI 32-CSI 41 | 0.019 |
| CSI 32-CSI 42 | 0.023 |
| CSI 32-CSI 43 | 0.008 |
| CSI 32-CSI 44 | 0.011 |
| CSI 32-CSI 45 | 0.012 |
| CSI 32-CSI 46 | 0.002 |
| CSI 32-CSI 47 | 0.010 |
| CSI 32-CSI 48 | 0.015 |
| CSI 32-CSI 49 | -0.006 |
| CSI 32-CSI 50 | -0.004 |
| CSI 32-CSI 51 | 0.004 |
| CSI 32-CSI 52 | -0.002 |
| CSI 32-CPO 01 | -0.054 |
| CSI 32-CPO 02 | -0.054 |
| CSI 32-CPO 03 | -0.059 |
| CSI 32-CPO 04 | -0.047 |
| CSI 32-CPO 05 | -0.069 |
| CSI 32-CPO 06 | -0.073 |
| CSI 32-CPO 07 | -0.063 |
| CSI 32-CPO 08 | -0.063 |
| CSI 32-CPO 09 | -0.046 |
| CSI 32-CPO 10 | -0.049 |
| CSI 32-CPO 11 | -0.067 |
| CSI 32-CPO 12 | -0.071 |
| CSI 32-CPO 13 | -0.048 |
| CSI 32-CPO 14 | -0.043 |
| CSI 32-CPO 15 | -0.062 |
| CSI 32-CPO 16 | -0.067 |
| CSI 32-CPO 17 | -0.021 |
| CSI 33-CSI 33 | 0.000 |
| CSI 33-CSI 34 | 0.035 |
| CSI 33-CSI 35 | 0.044 |
| CSI 33-CSI 36 | 0.148 |
| CSI 33-CSI 37 | 0.017 |
| CSI 33-CSI 38 | 0.000 |
| CSI 33-CSI 39 | 0.045 |
| CSI 33-CSI 40 | 0.012 |
| CSI 33-CSI 41 | 0.026 |
| CSI 33-CSI 42 | 0.029 |
| CSI 33-CSI 43 | 0.026 |
| CSI 33-CSI 44 | 0.037 |
| CSI 33-CSI 45 | 0.018 |
| CSI 33-CSI 46 | -0.002 |
| CSI 33-CSI 47 | 0.052 |
| CSI 33-CSI 48 | -0.007 |
| CSI 33-CSI 49 | 0.024 |
| CSI 33-CSI 50 | 0.005 |
| CSI 33-CSI 51 | -0.003 |
| CSI 33-CSI 52 | 0.004 |
| CSI 33-CPO 01 | -0.049 |
| CSI 33-CPO 02 | -0.049 |
| CSI 33-CPO 03 | -0.052 |
| CSI 33-CPO 04 | -0.050 |
| CSI 33-CPO 05 | -0.045 |
| CSI 33-CPO 06 | -0.045 |
| CSI 33-CPO 07 | -0.054 |
| CSI 33-CPO 08 | -0.054 |
| CSI 33-CPO 09 | -0.056 |
| CSI 33-CPO 10 | -0.048 |
| CSI 33-CPO 11 | -0.070 |
| CSI 33-CPO 12 | -0.064 |
| CSI 33-CPO 13 | -0.037 |
| CSI 33-CPO 14 | -0.047 |
| CSI 33-CPO 15 | -0.054 |
| CSI 33-CPO 16 | -0.060 |
| CSI 33-CPO 17 | 0.022 |
| CSI 34-CSI 34 | 0.000 |
| CSI 34-CSI 35 | 0.036 |
| CSI 34-CSI 36 | 0.012 |
| CSI 34-CSI 37 | 0.022 |
| CSI 34-CSI 38 | 0.029 |
| CSI 34-CSI 39 | 0.047 |
| CSI 34-CSI 40 | 0.014 |
| CSI 34-CSI 41 | 0.033 |
| CSI 34-CSI 42 | 0.035 |
| CSI 34-CSI 43 | 0.004 |
| CSI 34-CSI 44 | 0.017 |
| CSI 34-CSI 45 | 0.010 |
| CSI 34-CSI 46 | 0.033 |
| CSI 34-CSI 47 | 0.051 |
| CSI 34-CSI 48 | 0.020 |
| CSI 34-CSI 49 | 0.027 |
| CSI 34-CSI 50 | 0.006 |
| CSI 34-CSI 51 | 0.086 |
| CSI 34-CSI 52 | 0.035 |
| CSI 34-CPO 01 | -0.054 |
| CSI 34-CPO 02 | -0.054 |
| CSI 34-CPO 03 | -0.056 |
| CSI 34-CPO 04 | -0.052 |
| CSI 34-CPO 05 | -0.048 |
| CSI 34-CPO 06 | -0.052 |
| CSI 34-CPO 07 | -0.056 |
| CSI 34-CPO 08 | -0.056 |
| CSI 34-CPO 09 | -0.009 |
| CSI 34-CPO 10 | -0.068 |
| CSI 34-CPO 11 | -0.051 |
| CSI 34-CPO 12 | -0.057 |
| CSI 34-CPO 13 | -0.061 |
| CSI 34-CPO 14 | -0.018 |
| CSI 34-CPO 15 | -0.049 |
| CSI 34-CPO 16 | -0.045 |
| CSI 34-CPO 17 | -0.039 |
| CSI 35-CSI 35 | 0.000 |
| CSI 35-CSI 36 | 0.065 |
| CSI 35-CSI 37 | 0.043 |
| CSI 35-CSI 38 | 0.088 |
| CSI 35-CSI 39 | 0.048 |
| CSI 35-CSI 40 | 0.038 |
| CSI 35-CSI 41 | 0.002 |
| CSI 35-CSI 42 | 0.012 |
| CSI 35-CSI 43 | 0.009 |
| CSI 35-CSI 44 | 0.036 |
| CSI 35-CSI 45 | -0.002 |
| CSI 35-CSI 46 | -0.002 |
| CSI 35-CSI 47 | 0.016 |
| CSI 35-CSI 48 | 0.031 |
| CSI 35-CSI 49 | 0.009 |
| CSI 35-CSI 50 | -0.011 |
| CSI 35-CSI 51 | -0.006 |
| CSI 35-CSI 52 | 0.005 |
| CSI 35-CPO 01 | -0.045 |
| CSI 35-CPO 02 | -0.045 |
| CSI 35-CPO 03 | -0.054 |
| CSI 35-CPO 04 | -0.051 |
| CSI 35-CPO 05 | -0.053 |
| CSI 35-CPO 06 | -0.053 |
| CSI 35-CPO 07 | -0.059 |
| CSI 35-CPO 08 | -0.059 |
| CSI 35-CPO 09 | -0.027 |
| CSI 35-CPO 10 | -0.058 |
| CSI 35-CPO 11 | -0.043 |
| CSI 35-CPO 12 | -0.020 |
| CSI 35-CPO 13 | -0.025 |
| CSI 35-CPO 14 | -0.043 |
| CSI 35-CPO 15 | -0.051 |
| CSI 35-CPO 16 | -0.054 |
| CSI 35-CPO 17 | -0.024 |
| CSI 36-CSI 36 | 0.000 |
| CSI 36-CSI 37 | 0.037 |
| CSI 36-CSI 38 | -0.005 |
| CSI 36-CSI 39 | 0.041 |
| CSI 36-CSI 40 | 0.032 |
| CSI 36-CSI 41 | -0.001 |
| CSI 36-CSI 42 | -0.002 |
| CSI 36-CSI 43 | 0.045 |
| CSI 36-CSI 44 | 0.037 |
| CSI 36-CSI 45 | 0.014 |
| CSI 36-CSI 46 | -0.008 |
| CSI 36-CSI 47 | 0.045 |
| CSI 36-CSI 48 | -0.007 |
| CSI 36-CSI 49 | -0.008 |
| CSI 36-CSI 50 | 0.013 |
| CSI 36-CSI 51 | -0.003 |
| CSI 36-CSI 52 | -0.012 |
| CSI 36-CPO 01 | -0.059 |
| CSI 36-CPO 02 | -0.059 |
| CSI 36-CPO 03 | -0.066 |
| CSI 36-CPO 04 | -0.062 |
| CSI 36-CPO 05 | -0.060 |
| CSI 36-CPO 06 | -0.060 |
| CSI 36-CPO 07 | -0.061 |
| CSI 36-CPO 08 | -0.061 |
| CSI 36-CPO 09 | -0.038 |
| CSI 36-CPO 10 | -0.069 |
| CSI 36-CPO 11 | -0.069 |
| CSI 36-CPO 12 | -0.071 |
| CSI 36-CPO 13 | -0.058 |
| CSI 36-CPO 14 | -0.058 |
| CSI 36-CPO 15 | -0.064 |
| CSI 36-CPO 16 | -0.073 |
| CSI 36-CPO 17 | -0.044 |
| CSI 37-CSI 37 | 0.000 |
| CSI 37-CSI 38 | 0.003 |
| CSI 37-CSI 39 | 0.113 |
| CSI 37-CSI 40 | 0.109 |
| CSI 37-CSI 41 | 0.011 |
| CSI 37-CSI 42 | 0.037 |
| CSI 37-CSI 43 | 0.021 |
| CSI 37-CSI 44 | 0.005 |
| CSI 37-CSI 45 | 0.027 |
| CSI 37-CSI 46 | 0.047 |
| CSI 37-CSI 47 | 0.028 |
| CSI 37-CSI 48 | -0.003 |
| CSI 37-CSI 49 | 0.033 |
| CSI 37-CSI 50 | 0.018 |
| CSI 37-CSI 51 | -0.001 |
| CSI 37-CSI 52 | 0.016 |
| CSI 37-CPO 01 | -0.057 |
| CSI 37-CPO 02 | -0.057 |
| CSI 37-CPO 03 | -0.059 |
| CSI 37-CPO 04 | -0.054 |
| CSI 37-CPO 05 | -0.047 |
| CSI 37-CPO 06 | -0.047 |
| CSI 37-CPO 07 | -0.067 |
| CSI 37-CPO 08 | -0.057 |
| CSI 37-CPO 09 | -0.035 |
| CSI 37-CPO 10 | -0.065 |
| CSI 37-CPO 11 | -0.061 |
| CSI 37-CPO 12 | -0.069 |
| CSI 37-CPO 13 | -0.043 |
| CSI 37-CPO 14 | -0.029 |
| CSI 37-CPO 15 | -0.048 |
| CSI 37-CPO 16 | -0.051 |
| CSI 37-CPO 17 | -0.037 |
| CSI 38-CSI 38 | 0.000 |
| CSI 38-CSI 39 | 0.028 |
| CSI 38-CSI 40 | 0.003 |
| CSI 38-CSI 41 | 0.024 |
| CSI 38-CSI 42 | 0.027 |
| CSI 38-CSI 43 | 0.041 |
| CSI 38-CSI 44 | 0.031 |
| CSI 38-CSI 45 | 0.045 |
| CSI 38-CSI 46 | 0.021 |
| CSI 38-CSI 47 | 0.027 |
| CSI 38-CSI 48 | 0.065 |
| CSI 38-CSI 49 | 0.019 |
| CSI 38-CSI 50 | 0.043 |
| CSI 38-CSI 51 | 0.023 |
| CSI 38-CSI 52 | 0.034 |
| CSI 38-CPO 01 | -0.060 |
| CSI 38-CPO 02 | -0.060 |
| CSI 38-CPO 03 | -0.042 |
| CSI 38-CPO 04 | -0.050 |
| CSI 38-CPO 05 | -0.055 |
| CSI 38-CPO 06 | -0.055 |
| CSI 38-CPO 07 | -0.038 |
| CSI 38-CPO 08 | -0.038 |
| CSI 38-CPO 09 | -0.051 |
| CSI 38-CPO 10 | -0.037 |
| CSI 38-CPO 11 | -0.070 |
| CSI 38-CPO 12 | -0.035 |
| CSI 38-CPO 13 | -0.015 |
| CSI 38-CPO 14 | -0.031 |
| CSI 38-CPO 15 | -0.036 |
| CSI 38-CPO 16 | -0.035 |
| CSI 38-CPO 17 | -0.048 |
| CSI 39-CSI 39 | 0.000 |
| CSI 39-CSI 40 | 0.109 |
| CSI 39-CSI 41 | 0.043 |
| CSI 39-CSI 42 | 0.037 |
| CSI 39-CSI 43 | 0.016 |
| CSI 39-CSI 44 | -0.005 |
| CSI 39-CSI 45 | 0.022 |
| CSI 39-CSI 46 | 0.042 |
| CSI 39-CSI 47 | 0.032 |
| CSI 39-CSI 48 | 0.007 |
| CSI 39-CSI 49 | 0.034 |
| CSI 39-CSI 50 | 0.008 |
| CSI 39-CSI 51 | 0.008 |
| CSI 39-CSI 52 | 0.011 |
| CSI 39-CPO 01 | -0.057 |
| CSI 39-CPO 02 | -0.057 |
| CSI 39-CPO 03 | -0.064 |
| CSI 39-CPO 04 | -0.059 |
| CSI 39-CPO 05 | -0.047 |
| CSI 39-CPO 06 | -0.047 |
| CSI 39-CPO 07 | -0.067 |
| CSI 39-CPO 08 | -0.067 |
| CSI 39-CPO 09 | -0.025 |
| CSI 39-CPO 10 | -0.048 |
| CSI 39-CPO 11 | -0.072 |
| CSI 39-CPO 12 | -0.069 |
| CSI 39-CPO 13 | -0.042 |
| CSI 39-CPO 14 | -0.014 |
| CSI 39-CPO 15 | -0.053 |
| CSI 39-CPO 16 | -0.051 |
| CSI 39-CPO 17 | -0.011 |
| CSI 40-CSI 40 | 0.000 |
| CSI 40-CSI 41 | 0.035 |
| CSI 40-CSI 42 | 0.083 |
| CSI 40-CSI 43 | 0.016 |
| CSI 40-CSI 44 | -0.013 |
| CSI 40-CSI 45 | 0.014 |
| CSI 40-CSI 46 | 0.034 |
| CSI 40-CSI 47 | 0.015 |
| CSI 40-CSI 48 | -0.003 |
| CSI 40-CSI 49 | 0.014 |
| CSI 40-CSI 50 | 0.016 |
| CSI 40-CSI 51 | 0.007 |
| CSI 40-CSI 52 | 0.003 |
| CSI 40-CPO 01 | -0.057 |
| CSI 40-CPO 02 | -0.057 |
| CSI 40-CPO 03 | -0.051 |
| CSI 40-CPO 04 | -0.046 |
| CSI 40-CPO 05 | -0.047 |
| CSI 40-CPO 06 | -0.047 |
| CSI 40-CPO 07 | -0.067 |
| CSI 40-CPO 08 | -0.067 |
| CSI 40-CPO 09 | -0.039 |
| CSI 40-CPO 10 | -0.065 |
| CSI 40-CPO 11 | -0.072 |
| CSI 40-CPO 12 | -0.069 |
| CSI 40-CPO 13 | -0.058 |
| CSI 40-CPO 14 | -0.037 |
| CSI 40-CPO 15 | -0.053 |
| CSI 40-CPO 16 | -0.051 |
| CSI 40-CPO 17 | -0.024 |
| CSI 41-CSI 41 | 0.000 |
| CSI 41-CSI 42 | 0.057 |
| CSI 41-CSI 43 | 0.089 |
| CSI 41-CSI 44 | 0.014 |
| CSI 41-CSI 45 | -0.007 |
| CSI 41-CSI 46 | 0.011 |
| CSI 41-CSI 47 | 0.003 |
| CSI 41-CSI 48 | -0.029 |
| CSI 41-CSI 49 | 0.001 |
| CSI 41-CSI 50 | 0.026 |
| CSI 41-CSI 51 | -0.004 |
| CSI 41-CSI 52 | 0.027 |
| CSI 41-CPO 01 | -0.016 |
| CSI 41-CPO 02 | -0.016 |
| CSI 41-CPO 03 | -0.034 |
| CSI 41-CPO 04 | -0.022 |
| CSI 41-CPO 05 | -0.047 |
| CSI 41-CPO 06 | -0.047 |
| CSI 41-CPO 07 | -0.049 |
| CSI 41-CPO 08 | -0.049 |
| CSI 41-CPO 09 | -0.033 |
| CSI 41-CPO 10 | -0.065 |
| CSI 41-CPO 11 | -0.037 |
| CSI 41-CPO 12 | -0.062 |
| CSI 41-CPO 13 | -0.069 |
| CSI 41-CPO 14 | -0.044 |
| CSI 41-CPO 15 | -0.067 |
| CSI 41-CPO 16 | -0.062 |
| CSI 41-CPO 17 | -0.038 |
| CSI 42-CSI 42 | 0.000 |
| CSI 42-CSI 43 | 0.041 |
| CSI 42-CSI 44 | 0.029 |
| CSI 42-CSI 45 | 0.037 |
| CSI 42-CSI 46 | 0.118 |
| CSI 42-CSI 47 | 0.084 |
| CSI 42-CSI 48 | 0.008 |
| CSI 42-CSI 49 | 0.004 |
| CSI 42-CSI 50 | 0.013 |
| CSI 42-CSI 51 | -0.003 |
| CSI 42-CSI 52 | 0.009 |
| CSI 42-CPO 01 | -0.025 |
| CSI 42-CPO 02 | -0.025 |
| CSI 42-CPO 03 | -0.064 |
| CSI 42-CPO 04 | -0.058 |
| CSI 42-CPO 05 | -0.060 |
| CSI 42-CPO 06 | -0.064 |
| CSI 42-CPO 07 | -0.065 |
| CSI 42-CPO 08 | -0.065 |
| CSI 42-CPO 09 | -0.056 |
| CSI 42-CPO 10 | -0.079 |
| CSI 42-CPO 11 | -0.073 |
| CSI 42-CPO 12 | -0.071 |
| CSI 42-CPO 13 | -0.077 |
| CSI 42-CPO 14 | -0.058 |
| CSI 42-CPO 15 | -0.073 |
| CSI 42-CPO 16 | -0.067 |
| CSI 42-CPO 17 | -0.061 |
| CSI 43-CSI 43 | 0.000 |
| CSI 43-CSI 44 | 0.054 |
| CSI 43-CSI 45 | 0.031 |
| CSI 43-CSI 46 | 0.041 |
| CSI 43-CSI 47 | 0.017 |
| CSI 43-CSI 48 | -0.006 |
| CSI 43-CSI 49 | 0.001 |
| CSI 43-CSI 50 | 0.050 |
| CSI 43-CSI 51 | 0.023 |
| CSI 43-CSI 52 | 0.028 |
| CSI 43-CPO 01 | -0.057 |
| CSI 43-CPO 02 | -0.057 |
| CSI 43-CPO 03 | -0.058 |
| CSI 43-CPO 04 | -0.059 |
| CSI 43-CPO 05 | -0.037 |
| CSI 43-CPO 06 | -0.041 |
| CSI 43-CPO 07 | -0.040 |
| CSI 43-CPO 08 | -0.040 |
| CSI 43-CPO 09 | -0.039 |
| CSI 43-CPO 10 | -0.059 |
| CSI 43-CPO 11 | -0.067 |
| CSI 43-CPO 12 | -0.064 |
| CSI 43-CPO 13 | -0.058 |
| CSI 43-CPO 14 | -0.047 |
| CSI 43-CPO 15 | -0.048 |
| CSI 43-CPO 16 | -0.055 |
| CSI 43-CPO 17 | -0.062 |
| CSI 44-CSI 44 | 0.000 |
| CSI 44-CSI 45 | 0.148 |
| CSI 44-CSI 46 | 0.063 |
| CSI 44-CSI 47 | 0.087 |
| CSI 44-CSI 48 | 0.018 |
| CSI 44-CSI 49 | -0.001 |
| CSI 44-CSI 50 | 0.000 |
| CSI 44-CSI 51 | 0.022 |
| CSI 44-CSI 52 | 0.040 |
| CSI 44-CPO 01 | -0.064 |
| CSI 44-CPO 02 | -0.064 |
| CSI 44-CPO 03 | -0.048 |
| CSI 44-CPO 04 | -0.052 |
| CSI 44-CPO 05 | -0.057 |
| CSI 44-CPO 06 | -0.057 |
| CSI 44-CPO 07 | -0.044 |
| CSI 44-CPO 08 | -0.044 |
| CSI 44-CPO 09 | -0.044 |
| CSI 44-CPO 10 | -0.052 |
| CSI 44-CPO 11 | -0.068 |
| CSI 44-CPO 12 | -0.037 |
| CSI 44-CPO 13 | -0.028 |
| CSI 44-CPO 14 | -0.043 |
| CSI 44-CPO 15 | -0.013 |
| CSI 44-CPO 16 | -0.023 |
| CSI 44-CPO 17 | -0.063 |
| CSI 45-CSI 45 | 0.000 |
| CSI 45-CSI 46 | 0.131 |
| CSI 45-CSI 47 | 0.044 |
| CSI 45-CSI 48 | 0.008 |
| CSI 45-CSI 49 | 0.005 |
| CSI 45-CSI 50 | 0.025 |
| CSI 45-CSI 51 | -0.007 |
| CSI 45-CSI 52 | 0.031 |
| CSI 45-CPO 01 | -0.072 |
| CSI 45-CPO 02 | -0.072 |
| CSI 45-CPO 03 | -0.061 |
| CSI 45-CPO 04 | -0.064 |
| CSI 45-CPO 05 | -0.060 |
| CSI 45-CPO 06 | -0.060 |
| CSI 45-CPO 07 | -0.048 |
| CSI 45-CPO 08 | -0.048 |
| CSI 45-CPO 09 | -0.052 |
| CSI 45-CPO 10 | -0.052 |
| CSI 45-CPO 11 | -0.072 |
| CSI 45-CPO 12 | -0.056 |
| CSI 45-CPO 13 | -0.048 |
| CSI 45-CPO 14 | -0.047 |
| CSI 45-CPO 15 | -0.022 |
| CSI 45-CPO 16 | -0.027 |
| CSI 45-CPO 17 | -0.067 |
| CSI 46-CSI 46 | 0.000 |
| CSI 46-CSI 47 | 0.123 |
| CSI 46-CSI 48 | 0.011 |
| CSI 46-CSI 49 | 0.026 |
| CSI 46-CSI 50 | 0.014 |
| CSI 46-CSI 51 | 0.019 |
| CSI 46-CSI 52 | 0.034 |
| CSI 46-CPO 01 | -0.061 |
| CSI 46-CPO 02 | -0.061 |
| CSI 46-CPO 03 | -0.059 |
| CSI 46-CPO 04 | -0.054 |
| CSI 46-CPO 05 | -0.065 |
| CSI 46-CPO 06 | -0.065 |
| CSI 46-CPO 07 | -0.073 |
| CSI 46-CPO 08 | -0.073 |
| CSI 46-CPO 09 | -0.052 |
| CSI 46-CPO 10 | -0.079 |
| CSI 46-CPO 11 | -0.073 |
| CSI 46-CPO 12 | -0.071 |
| CSI 46-CPO 13 | -0.072 |
| CSI 46-CPO 14 | -0.032 |
| CSI 46-CPO 15 | -0.061 |
| CSI 46-CPO 16 | -0.063 |
| CSI 46-CPO 17 | -0.054 |
| CSI 47-CSI 47 | 0.000 |
| CSI 47-CSI 48 | 0.029 |
| CSI 47-CSI 49 | 0.023 |
| CSI 47-CSI 50 | 0.015 |
| CSI 47-CSI 51 | 0.026 |
| CSI 47-CSI 52 | 0.072 |
| CSI 47-CPO 01 | -0.075 |
| CSI 47-CPO 02 | -0.075 |
| CSI 47-CPO 03 | -0.068 |
| CSI 47-CPO 04 | -0.068 |
| CSI 47-CPO 05 | -0.067 |
| CSI 47-CPO 06 | -0.067 |
| CSI 47-CPO 07 | -0.075 |
| CSI 47-CPO 08 | -0.075 |
| CSI 47-CPO 09 | -0.011 |
| CSI 47-CPO 10 | -0.079 |
| CSI 47-CPO 11 | -0.075 |
| CSI 47-CPO 12 | -0.073 |
| CSI 47-CPO 13 | -0.072 |
| CSI 47-CPO 14 | -0.039 |
| CSI 47-CPO 15 | -0.063 |
| CSI 47-CPO 16 | -0.065 |
| CSI 47-CPO 17 | -0.046 |
| CSI 48-CSI 48 | 0.000 |
| CSI 48-CSI 49 | 0.042 |
| CSI 48-CSI 50 | 0.032 |
| CSI 48-CSI 51 | 0.052 |
| CSI 48-CSI 52 | 0.058 |
| CSI 48-CPO 01 | -0.071 |
| CSI 48-CPO 02 | -0.071 |
| CSI 48-CPO 03 | -0.062 |
| CSI 48-CPO 04 | -0.069 |
| CSI 48-CPO 05 | -0.062 |
| CSI 48-CPO 06 | -0.066 |
| CSI 48-CPO 07 | -0.059 |
| CSI 48-CPO 08 | -0.059 |
| CSI 48-CPO 09 | 0.027 |
| CSI 48-CPO 10 | -0.070 |
| CSI 48-CPO 11 | -0.073 |
| CSI 48-CPO 12 | -0.038 |
| CSI 48-CPO 13 | -0.037 |
| CSI 48-CPO 14 | -0.053 |
| CSI 48-CPO 15 | -0.066 |
| CSI 48-CPO 16 | -0.041 |
| CSI 48-CPO 17 | -0.059 |
| CSI 49-CSI 49 | 0.000 |
| CSI 49-CSI 50 | 0.064 |
| CSI 49-CSI 51 | 0.056 |
| CSI 49-CSI 52 | 0.056 |
| CSI 49-CPO 01 | -0.062 |
| CSI 49-CPO 02 | -0.062 |
| CSI 49-CPO 03 | -0.059 |
| CSI 49-CPO 04 | -0.069 |
| CSI 49-CPO 05 | -0.043 |
| CSI 49-CPO 06 | -0.047 |
| CSI 49-CPO 07 | -0.067 |
| CSI 49-CPO 08 | -0.057 |
| CSI 49-CPO 09 | -0.044 |
| CSI 49-CPO 10 | -0.071 |
| CSI 49-CPO 11 | -0.065 |
| CSI 49-CPO 12 | -0.063 |
| CSI 49-CPO 13 | -0.041 |
| CSI 49-CPO 14 | -0.038 |
| CSI 49-CPO 15 | -0.048 |
| CSI 49-CPO 16 | -0.046 |
| CSI 49-CPO 17 | -0.045 |
| CSI 50-CSI 50 | 0.000 |
| CSI 50-CSI 51 | 0.080 |
| CSI 50-CSI 52 | 0.054 |
| CSI 50-CPO 01 | -0.070 |
| CSI 50-CPO 02 | -0.070 |
| CSI 50-CPO 03 | -0.053 |
| CSI 50-CPO 04 | -0.060 |
| CSI 50-CPO 05 | -0.055 |
| CSI 50-CPO 06 | -0.059 |
| CSI 50-CPO 07 | -0.067 |
| CSI 50-CPO 08 | -0.056 |
| CSI 50-CPO 09 | -0.062 |
| CSI 50-CPO 10 | -0.075 |
| CSI 50-CPO 11 | -0.064 |
| CSI 50-CPO 12 | -0.065 |
| CSI 50-CPO 13 | -0.048 |
| CSI 50-CPO 14 | -0.044 |
| CSI 50-CPO 15 | -0.068 |
| CSI 50-CPO 16 | -0.064 |
| CSI 50-CPO 17 | -0.049 |
| CSI 51-CSI 51 | 0.000 |
| CSI 51-CSI 52 | 0.063 |
| CSI 51-CPO 01 | -0.067 |
| CSI 51-CPO 02 | -0.067 |
| CSI 51-CPO 03 | -0.041 |
| CSI 51-CPO 04 | -0.048 |
| CSI 51-CPO 05 | -0.050 |
| CSI 51-CPO 06 | -0.058 |
| CSI 51-CPO 07 | -0.061 |
| CSI 51-CPO 08 | -0.061 |
| CSI 51-CPO 09 | -0.047 |
| CSI 51-CPO 10 | -0.067 |
| CSI 51-CPO 11 | -0.073 |
| CSI 51-CPO 12 | -0.063 |
| CSI 51-CPO 13 | -0.050 |
| CSI 51-CPO 14 | -0.034 |
| CSI 51-CPO 15 | -0.050 |
| CSI 51-CPO 16 | -0.042 |
| CSI 51-CPO 17 | -0.027 |
| CSI 52-CSI 52 | 0.000 |
| CSI 52-CPO 01 | -0.070 |
| CSI 52-CPO 02 | -0.070 |
| CSI 52-CPO 03 | -0.063 |
| CSI 52-CPO 04 | -0.072 |
| CSI 52-CPO 05 | -0.068 |
| CSI 52-CPO 06 | -0.072 |
| CSI 52-CPO 07 | -0.064 |
| CSI 52-CPO 08 | -0.064 |
| CSI 52-CPO 09 | -0.016 |
| CSI 52-CPO 10 | -0.070 |
| CSI 52-CPO 11 | -0.077 |
| CSI 52-CPO 12 | -0.056 |
| CSI 52-CPO 13 | -0.050 |
| CSI 52-CPO 14 | -0.047 |
| CSI 52-CPO 15 | -0.065 |
| CSI 52-CPO 16 | -0.063 |
| CSI 52-CPO 17 | -0.070 |
| CPO 01-CPO 01 | 0.000 |
| CPO 01-CPO 02 | 0.643 |
| CPO 01-CPO 03 | 0.398 |
| CPO 01-CPO 04 | 0.426 |
| CPO 01-CPO 05 | 0.122 |
| CPO 01-CPO 06 | 0.129 |
| CPO 01-CPO 07 | 0.073 |
| CPO 01-CPO 08 | 0.053 |
| CPO 01-CPO 09 | 0.004 |
| CPO 01-CPO 10 | 0.002 |
| CPO 01-CPO 11 | 0.075 |
| CPO 01-CPO 12 | 0.015 |
| CPO 01-CPO 13 | 0.001 |
| CPO 01-CPO 14 | 0.106 |
| CPO 01-CPO 15 | -0.018 |
| CPO 01-CPO 16 | 0.057 |
| CPO 01-CPO 17 | 0.057 |
| CPO 02-CPO 02 | 0.000 |
| CPO 02-CPO 03 | 0.398 |
| CPO 02-CPO 04 | 0.426 |
| CPO 02-CPO 05 | 0.122 |
| CPO 02-CPO 06 | 0.129 |
| CPO 02-CPO 07 | 0.073 |
| CPO 02-CPO 08 | 0.053 |
| CPO 02-CPO 09 | 0.004 |
| CPO 02-CPO 10 | 0.002 |
| CPO 02-CPO 11 | 0.075 |
| CPO 02-CPO 12 | 0.015 |
| CPO 02-CPO 13 | 0.001 |
| CPO 02-CPO 14 | 0.106 |
| CPO 02-CPO 15 | -0.018 |
| CPO 02-CPO 16 | 0.057 |
| CPO 02-CPO 17 | 0.057 |
| CPO 03-CPO 03 | 0.000 |
| CPO 03-CPO 04 | 0.508 |
| CPO 03-CPO 05 | 0.097 |
| CPO 03-CPO 06 | 0.104 |
| CPO 03-CPO 07 | 0.047 |
| CPO 03-CPO 08 | 0.047 |
| CPO 03-CPO 09 | 0.036 |
| CPO 03-CPO 10 | 0.065 |
| CPO 03-CPO 11 | 0.097 |
| CPO 03-CPO 12 | 0.059 |
| CPO 03-CPO 13 | 0.058 |
| CPO 03-CPO 14 | 0.050 |
| CPO 03-CPO 15 | 0.018 |
| CPO 03-CPO 16 | 0.130 |
| CPO 03-CPO 17 | 0.081 |
| CPO 04-CPO 04 | 0.000 |
| CPO 04-CPO 05 | 0.075 |
| CPO 04-CPO 06 | 0.082 |
| CPO 04-CPO 07 | 0.055 |
| CPO 04-CPO 08 | 0.055 |
| CPO 04-CPO 09 | 0.017 |
| CPO 04-CPO 10 | 0.040 |
| CPO 04-CPO 11 | 0.100 |
| CPO 04-CPO 12 | 0.020 |
| CPO 04-CPO 13 | 0.046 |
| CPO 04-CPO 14 | 0.051 |
| CPO 04-CPO 15 | 0.022 |
| CPO 04-CPO 16 | 0.113 |
| CPO 04-CPO 17 | 0.096 |
| CPO 05-CPO 05 | 0.000 |
| CPO 05-CPO 06 | 0.668 |
| CPO 05-CPO 07 | 0.226 |
| CPO 05-CPO 08 | 0.205 |
| CPO 05-CPO 09 | 0.065 |
| CPO 05-CPO 10 | 0.045 |
| CPO 05-CPO 11 | 0.120 |
| CPO 05-CPO 12 | 0.101 |
| CPO 05-CPO 13 | 0.036 |
| CPO 05-CPO 14 | 0.052 |
| CPO 05-CPO 15 | 0.049 |
| CPO 05-CPO 16 | 0.100 |
| CPO 05-CPO 17 | 0.069 |
| CPO 06-CPO 06 | 0.000 |
| CPO 06-CPO 07 | 0.229 |
| CPO 06-CPO 08 | 0.208 |
| CPO 06-CPO 09 | 0.065 |
| CPO 06-CPO 10 | 0.045 |
| CPO 06-CPO 11 | 0.135 |
| CPO 06-CPO 12 | 0.108 |
| CPO 06-CPO 13 | 0.036 |
| CPO 06-CPO 14 | 0.067 |
| CPO 06-CPO 15 | 0.049 |
| CPO 06-CPO 16 | 0.096 |
| CPO 06-CPO 17 | 0.077 |
| CPO 07-CPO 07 | 0.000 |
| CPO 07-CPO 08 | 0.784 |
| CPO 07-CPO 09 | -0.001 |
| CPO 07-CPO 10 | 0.102 |
| CPO 07-CPO 11 | 0.160 |
| CPO 07-CPO 12 | 0.104 |
| CPO 07-CPO 13 | 0.070 |
| CPO 07-CPO 14 | 0.097 |
| CPO 07-CPO 15 | 0.041 |
| CPO 07-CPO 16 | 0.049 |
| CPO 07-CPO 17 | 0.028 |
| CPO 08-CPO 08 | 0.000 |
| CPO 08-CPO 09 | -0.001 |
| CPO 08-CPO 10 | 0.102 |
| CPO 08-CPO 11 | 0.171 |
| CPO 08-CPO 12 | 0.104 |
| CPO 08-CPO 13 | 0.081 |
| CPO 08-CPO 14 | 0.097 |
| CPO 08-CPO 15 | 0.041 |
| CPO 08-CPO 16 | 0.049 |
| CPO 08-CPO 17 | 0.028 |
| CPO 09-CPO 09 | 0.000 |
| CPO 09-CPO 10 | -0.026 |
| CPO 09-CPO 11 | 0.003 |
| CPO 09-CPO 12 | -0.011 |
| CPO 09-CPO 13 | -0.044 |
| CPO 09-CPO 14 | -0.054 |
| CPO 09-CPO 15 | -0.032 |
| CPO 09-CPO 16 | -0.032 |
| CPO 09-CPO 17 | -0.018 |
| CPO 10-CPO 10 | 0.000 |
| CPO 10-CPO 11 | 0.140 |
| CPO 10-CPO 12 | 0.155 |
| CPO 10-CPO 13 | 0.797 |
| CPO 10-CPO 14 | 0.032 |
| CPO 10-CPO 15 | 0.149 |
| CPO 10-CPO 16 | 0.132 |
| CPO 10-CPO 17 | 0.120 |
| CPO 11-CPO 11 | 0.000 |
| CPO 11-CPO 12 | 0.461 |
| CPO 11-CPO 13 | 0.119 |
| CPO 11-CPO 14 | 0.033 |
| CPO 11-CPO 15 | 0.020 |
| CPO 11-CPO 16 | 0.144 |
| CPO 11-CPO 17 | 0.115 |
| CPO 12-CPO 12 | 0.000 |
| CPO 12-CPO 13 | 0.157 |
| CPO 12-CPO 14 | 0.111 |
| CPO 12-CPO 15 | 0.045 |
| CPO 12-CPO 16 | 0.106 |
| CPO 12-CPO 17 | 0.049 |
| CPO 13-CPO 13 | 0.000 |
| CPO 13-CPO 14 | 0.069 |
| CPO 13-CPO 15 | 0.114 |
| CPO 13-CPO 16 | 0.095 |
| CPO 13-CPO 17 | 0.086 |
| CPO 14-CPO 14 | 0.000 |
| CPO 14-CPO 15 | 0.172 |
| CPO 14-CPO 16 | 0.070 |
| CPO 14-CPO 17 | 0.078 |
| CPO 15-CPO 15 | 0.000 |
| CPO 15-CPO 16 | 0.448 |
| CPO 15-CPO 17 | 0.262 |
| CPO 16-CPO 16 | 0.000 |
| CPO 16-CPO 17 | 0.048 |
| CPO 17-CPO 17 | 0.000 |
