# Supplementary material for: High genetic diversity and demographic history of captive Siamese and Saltwater crocodiles suggest the first step toward the establishment of a breeding and reintroduction program in Thailand
Source: PLoS One. 2017 Sep 27;12(9):e0184526. doi: 10.1371/journal.pone.0184526 (PMC5617146; doi:10.1371/journal.pone.0184526)
Supplement: S13 Table — Detailed information for all crocodile individuals is presented in S1 Table. (DOCX) [file pone.0184526.s014.docx]

**S13 Table. Pairwise inbreeding coefficients for all 69 crocodile individuals.** Detailed information for all crocodile individuals is presented in S1 Table.

| Individuals | Inbreeding coefficients |
| --- | --- |
| CSI 01 | 0.132 |
| CSI 02 | 0.149 |
| CSI 03 | 0.295 |
| CSI 04 | 0.250 |
| CSI 05 | 0.824 |
| CSI 06 | 0.200 |
| CSI 07 | 0.166 |
| CSI 08 | 0.136 |
| CSI 09 | 0.437 |
| CSI 10 | 0.072 |
| CSI 11 | 0.380 |
| CSI 12 | 0.168 |
| CSI 13 | 0.063 |
| CSI 14 | 0.212 |
| CSI 15 | 0.116 |
| CSI 16 | 0.022 |
| CSI 17 | 0.310 |
| CSI 18 | 0.117 |
| CSI 19 | 0.081 |
| CSI 20 | 0.164 |
| CSI 21 | 0.176 |
| CSI 22 | 0.026 |
| CSI 23 | 0.036 |
| CSI 24 | 0.012 |
| CSI 25 | 0.231 |
| CSI 26 | 0.469 |
| CSI 27 | 0.007 |
| CSI 28 | 0.035 |
| CSI 29 | 0.026 |
| CSI 30 | 0.056 |
| CSI 31 | 0.074 |
| CSI 32 | 0.102 |
| CSI 33 | 0.158 |
| CSI 34 | -0.016 |
| CSI 35 | 0.077 |
| CSI 36 | 0.214 |
| CSI 37 | 0.145 |
| CSI 38 | 0.019 |
| CSI 39 | 0.054 |
| CSI 40 | 0.070 |
| CSI 41 | 0.041 |
| CSI 42 | 0.182 |
| CSI 43 | 0.098 |
| CSI 44 | 0.022 |
| CSI 45 | -0.001 |
| CSI 46 | 0.090 |
| CSI 47 | 0.101 |
| CSI 48 | 0.052 |
| CSI 49 | 0.012 |
| CSI 50 | 0.025 |
| CSI 51 | 0.253 |
| CSI 52 | 0.007 |
| CPO 01 | 0.151 |
| CPO 02 | 0.151 |
| CPO 03 | 0.115 |
| CPO 04 | 0.145 |
| CPO 05 | 0.263 |
| CPO 06 | 0.293 |
| CPO 07 | 0.149 |
| CPO 08 | 0.149 |
| CPO 09 | 0.666 |
| CPO 10 | 0.579 |
| CPO 11 | 0.567 |
| CPO 12 | 0.573 |
| CPO 13 | 0.157 |
| CPO 14 | 0.617 |
| CPO 15 | 0.645 |
| CPO 16 | 0.324 |
| CPO 17 | 0.145 |
